# The changing patterns of cardiovascular diseases and their risk factors in the states of India: the Global Burden of Disease Study 1990–2016

**DOI:** 10.1016/S2214-109X(18)30407-8

**Published:** 2018-09-12

**Authors:** India State-Level Disease Burden Initiative CVD Collaborators, Dorairaj Prabhakaran, Panniyammakal Jeemon, Meenakshi Sharma, Gregory A Roth, Catherine Johnson, Sivadasanpillai Harikrishnan, Rajeev Gupta, Jeyaraj D Pandian, Nitish Naik, Ambuj Roy, R S Dhaliwal, Denis Xavier, Raman K Kumar, Nikhil Tandon, Prashant Mathur, D K Shukla, Ravi Mehrotra, K Venugopal, G Anil Kumar, Chris M Varghese, Melissa Furtado, Pallavi Muraleedharan, Rizwan S Abdulkader, Tahiya Alam, Ranjit M Anjana, Monika Arora, Anil Bhansali, Deeksha Bhardwaj, Eesh Bhatia, Joy K Chakma, Pankaj Chaturvedi, Eliza Dutta, Scott Glenn, Prakash C Gupta, Sarah C Johnson, Tanvir Kaur, Sanjay Kinra, Anand Krishnan, Michael Kutz, Manu R Mathur, Viswanathan Mohan, Satinath Mukhopadhyay, Minh Nguyen, Christopher M Odell, Anu M Oommen, Sanghamitra Pati, Martin Pletcher, Kameshwar Prasad, Paturi V Rao, Chander Shekhar, Dhirendra N Sinha, P N Sylaja, J S Thakur, Kavumpurathu R Thankappan, Nihal Thomas, Simon Yadgir, Chittaranjan S Yajnik, Geevar Zachariah, Ben Zipkin, Stephen S Lim, Mohsen Naghavi, Rakhi Dandona, Theo Vos, Christopher J L Murray, K Srinath Reddy, Soumya Swaminathan, Lalit Dandona

## Abstract

**Background:**

The burden of cardiovascular diseases is increasing in India, but a systematic understanding of its distribution and time trends across all the states is not readily available. In this report, we present a detailed analysis of how the patterns of cardiovascular diseases and major risk factors have changed across the states of India between 1990 and 2016.

**Methods:**

We analysed the prevalence and disability-adjusted life-years (DALYs) due to cardiovascular diseases and the major component causes in the states of India from 1990 to 2016, using all accessible data sources as part of the Global Burden of Diseases, Injuries, and Risk Factors Study 2016. We placed states into four groups based on epidemiological transition level (ETL), defined using the ratio of DALYs from communicable diseases to those from non-communicable diseases and injuries combined, with a low ratio denoting high ETL and vice versa. We assessed heterogeneity in the burden of major cardiovascular diseases across the states of India, and the contribution of risk factors to cardiovascular diseases. We calculated 95% uncertainty intervals (UIs) for the point estimates.

**Findings:**

Overall, cardiovascular diseases contributed 28·1% (95% UI 26·5–29·1) of the total deaths and 14·1% (12·9–15·3) of the total DALYs in India in 2016, compared with 15·2% (13·7–16·2) and 6·9% (6·3–7·4), respectively, in 1990. In 2016, there was a nine times difference between states in the DALY rate for ischaemic heart disease, a six times difference for stroke, and a four times difference for rheumatic heart disease. 23·8 million (95% UI 22·6–25·0) prevalent cases of ischaemic heart disease were estimated in India in 2016, and 6·5 million (6·3–6·8) prevalent cases of stroke, a 2·3 times increase in both disorders from 1990. The age-standardised prevalence of both ischaemic heart disease and stroke increased in all ETL state groups between 1990 and 2016, whereas that of rheumatic heart disease decreased; the increase for ischaemic heart disease was highest in the low ETL state group. 53·4% (95% UI 52·6–54·6) of crude deaths due to cardiovascular diseases in India in 2016 were among people younger than 70 years, with a higher proportion in the low ETL state group. The leading overlapping risk factors for cardiovascular diseases in 2016 included dietary risks (56·4% [95% CI 48·5–63·9] of cardiovascular disease DALYs), high systolic blood pressure (54·6% [49·0–59·8]), air pollution (31·1% [29·0–33·4]), high total cholesterol (29·4% [24·3–34·8]), tobacco use (18·9% [16·6–21·3]), high fasting plasma glucose (16·7% [11·4–23·5]), and high body-mass index (14·7% [8·3–22·0]). The prevalence of high systolic blood pressure, high total cholesterol, and high fasting plasma glucose increased generally across all ETL state groups from 1990 to 2016, but this increase was variable across the states; the prevalence of smoking decreased during this period in all ETL state groups.

**Interpretation:**

The burden from the leading cardiovascular diseases in India—ischaemic heart disease and stroke—varies widely between the states. Their increasing prevalence and that of several major risk factors in every part of India, especially the highest increase in the prevalence of ischaemic heart disease in the less developed low ETL states, indicates the need for urgent policy and health system response appropriate for the situation in each state.

**Funding:**

Bill & Melinda Gates Foundation; and Indian Council of Medical Research, Department of Health Research, Ministry of Health and Family Welfare, Government of India.

## Introduction

Cardiovascular diseases are the leading cause of disease burden and deaths globally.^1^, ^2^, ^3^ The UN, alarmed by the increasing burden of non-communicable diseases (NCDs) and high disease severity and case-fatality in low-income and middle-income countries compared with high-income countries, acknowledged in 2012 that the rising burden of NCDs was one of the major threats to sustainable development in the 21st century.^4^, ^5^, ^6^, ^7^, ^8^, ^9^ WHO subsequently developed targets for prevention and control of NCDs in 2013, which included 25% relative reduction in overall mortality from cardiovascular diseases, 25% relative reduction in prevalence of high blood pressure, halting the rise in diabetes and obesity, and ensuring that at least 50% of patients with cardiovascular diseases have access to relevant drugs and medical counselling by 2025.^10^, ^11^ The Sustainable Development Goals also include a target to reduce premature deaths due to NCDs to a third of total premature deaths by 2030, emphasising the need for multisectoral national policies to facilitate the prevention and control of burden from NCDs.^12^, ^13^ Since cardiovascular diseases make up a large part of NCDs, the National Health Policy 2017 of India aims to reduce 25% of premature deaths from cardiovascular diseases besides screening and treatment of 80% of hypertensive patients by 2025.^14^

**Research in context**

**Evidence before this study**

We searched PubMed and publicly available reports on March 29, 2018, without language or publication date restrictions, for estimates of the burden of cardiovascular diseases across the states of India, using the terms “burden”, “cardiovascular diseases”, “cause of death”, “cerebrovascular disorders”, “coronary heart disease”, “CVD”, “DALY”, “death”, “epidemiology”, “India”, “ischaemic heart disease”, “morbidity”, “mortality”, “prevalence”, “rheumatic heart disease”, “stroke”, and “trends”. Previous studies have noted the increasing burden of cardiovascular diseases and their risk factors over time in India, and attempts have been made to assess the variations of cardiovascular disease burden and their major risk factors for a number of states. However, we did not find any comprehensive report on the trends of prevalence, deaths, and disability-adjusted life-years (DALYs) from different cardiovascular diseases in every state of India over a long period, which is needed to inform effective policy making for the heterogeneous 1·3 billion population of India.

**Added value of this study**

This study provides comprehensive estimates of the burden due to cardiovascular diseases in every state of India from 1990 to 2016, based on all accessible data and using the standardised Global Burden of Diseases, Injuries, and Risk Factors Study methodology. These findings highlight that the prevalent cases of ischaemic heart disease and stroke have more than doubled in India from 1990 to 2016, with an increase in prevalence in every state of the country. Although the DALY rate due to ischaemic heart disease is currently higher in the more developed states of India, this study shows that the increase in age-standardised DALY rates from 1990 was higher in less developed states. It also reports that more than half of cardiovascular disease deaths in India in 2016 were in people younger than 70 years, with this proportion being higher in the relatively less developed states. The findings emphasise that, although the prevalence of cardiovascular disease risk factors varied considerably across the states of India, the prevalence of high systolic blood pressure, high total cholesterol, and high fasting plasma glucose increased across all state groups since 1990.

**Implications of all the available evidence**

Our systematic assessment of cardiovascular diseases in all states of India from 1990 to 2016 indicates an urgent need for controlling the increasing prevalence of ischaemic heart disease and stroke, and the adverse effect of these disorders, in all parts of the country, with particular attention to the less developed states of India where the increase in prevalence of ischaemic heart disease has been the highest. Further planning to control cardiovascular diseases in India could benefit from using these comprehensive state-specific trends as reference.

In a country of 1·3 billion people with cultural and lifestyle diversities, several previous attempts have been made to compile the burden of cardiovascular diseases in different parts of India.^15^, ^16^, ^17^, ^18^, ^19^, ^20^ However, there is no comprehensive analysis available that allows comparison of the trends in cardiovascular diseases and their risk factors across the states of India over time in a single framework. In the federal framework of Indian administration, health is mostly a state subject, with approximately two-thirds of health-care funding from the state exchequer.^21^, ^22^ The India State-Level Disease Burden Initiative has reported a varied epidemiological transition among the states of India from 1990 to 2016 as part of the Global Burden of Diseases, Injuries, and Risk Factors (GBD) Study 2016.^23^, ^24^ Here, we report time trends and heterogeneity among states for cardiovascular diseases from 1990 to 2016, and for the major risk factors for cardiovascular diseases.

## Methods

### Overview

The India State-Level Disease Burden Initiative has reported overall trends in diseases, injuries, and risk factors from 1990 to 2016 for every state of India.^23^, ^24^ This analysis was done as part of GBD 2016, which estimated disease burden due to 333 diseases and injuries and 84 risk factors using all available data sources that could be accessed and that met inclusion criteria. Disease grouping and risk grouping in GBD 2016 were organised into three broad categories and four levels, respectively. The India State-Level Disease Burden Initiative benefited from the efforts of several expert groups and a large network of collaborators in India to identify all available data sources that could be accessed, to assess their scope and quality for inclusion, and to participate in the analysis and interpretation of findings. The Health Ministry Screening Committee at the Indian Council of Medical Research and the ethics committee of the Public Health Foundation of India approved the work of this initiative. A detailed description of metrics and analytical approaches used in GBD 2016 has been reported elsewhere.^1^, ^2^, ^3^, ^25^, ^26^ Here, we report findings on cardiovascular diseases and the major component causes, and main risk factors for cardiovascular diseases, across the states of India from 1990 to 2016. GBD 2016 methods relevant to this Article are described in the appendix (pp 3–41), with the key points summarised here.

### Estimation of prevalence and years lived with disability

We estimated the prevalence of cardiovascular diseases and the major component causes by location, age, sex, and year using DisMod-MR, version 2.1, a disease modelling computational program that is the standard GBD modelling approach for non-fatal health outcomes.^3^ This approach entailed identification of all available data sources that could be accessed and their assessment for data extraction based on inclusion criteria, estimation of cause-specific prevalence using DisMod-MR modelling, ascertainment of severity distributions of sequelae, incorporation of disability weights to quantify severity, comorbidity adjustment of sequelae, and calculation of years lived with disability (YLDs) from prevalence and disability weights for each location, age, sex, and year.^3^

The major data inputs for prevalence estimation of cardiovascular diseases and the major component causes in India included population-representative surveys and cohort studies, disease registries, hospital-based data, and a wide array of published and unpublished studies (appendix pp 42–56). We estimated the prevalence of ischaemic heart disease and its sequelae from a combination of cause-specific death rates, prevalence, incidence, standardised mortality ratios, excess mortality rates, and relative risks.^3^, ^27^ We estimated stroke prevalence from incidence and excess mortality data for the first-ever acute stroke models, and survivor incidence, excess mortality, and prevalence data for the chronic stroke model.^3^, ^28^ Data inputs for prevalence and cause-specific mortality rates of rheumatic heart disease from an endemic and non-endemic location model were used to produce prevalence estimates of rheumatic heart disease by location, age, sex, and year.^3^, ^29^

### Estimation of deaths, years of life lost, and disability-adjusted life-years

Among the all-cause mortality rates, we estimated mortality from cardiovascular diseases and each of the major component causes with the GBD Cause of Death Ensemble modelling approach, which uses all available data sources that could be accessed along with covariates to develop a series of plausible models and, eventually, the best ensemble predictive model to produce estimates of deaths and years of life lost (YLLs) due to premature mortality by location, age, sex, and year.^1^, ^2^, ^27^ We calculated YLLs from age at death and GBD normative standard life expectancy at each age. We computed disability-adjusted life-years (DALYs), a summary measure of total health loss, by adding YLLs and YLDs for each cardiovascular disease subcause, age, and sex grouping by location. The main data inputs for estimation of mortality from cardiovascular diseases and the major component causes in India included Sample Registration System cause of death data, Medical Certification of Cause of Death data, and other verbal autopsy studies (appendix pp 42–56).

### Estimation of risk factor exposure and attributable disease burden

We used the GBD comparative risk assessment framework to estimate cardiovascular disease-related risk factor exposure and attributable disease burden, as explained elsewhere.^26^ We collated exposure data for risk factors with a categorical or continuous distribution from all available data sources that could be accessed, including survey and other data, adjusted using age–sex splitting, and strengthened with incorporation of covariates for modelling. The modelling approaches integrated multiple data inputs and borrowed information across age, time, and location to produce the best possible estimates of risk exposure by location, age, sex, and year. For each risk factor, the theoretical minimum risk exposure level was established as the lowest level of risk exposure below which its relation with a disease outcome is not supported by the available evidence. We used estimates of mean risk factor exposure, strengthened by covariates, to calculate summary exposure values for each risk—a metric ranging from 0% to 100%—to describe the risk-weighted exposure for a population or risk-weighted prevalence of exposure.

The estimation of attributable disease burden included ascertainment of the relative risk of disease outcomes for risk exposure–disease outcome pairs with sufficient evidence of a causal relation in randomised control trials, prospective cohorts, or case-control studies, as assessed using an approach similar to the World Cancer Research Fund grading system. We estimated population attributable fractions for diseases caused by each risk factor.^26^ Estimates of deaths, YLLs, YLDs, and DALYs attributable to each risk factor were produced by location, age, sex, and year. A detailed description of exposure and attributable disease burden estimation for the major risk factors associated with cardiovascular diseases, including GBD exposure definitions and statistical modelling, is in the appendix (pp 3–41) and published elsewhere.^26^

The major data inputs for the risk factors of cardio_vascular diseases in India included dietary and nutrition surveys by the National Nutrition Monitoring Bureau; national household surveys such as the National Family Health Survey, District Level Household Survey, and Annual Health Survey; air pollution monitoring and satellite data; youth and adult tobacco surveys; household consumer expenditure surveys of the National Sample Survey Organisation; and various other population-based surveys (appendix pp 42–56).

GBD uses covariates—explanatory variables that have a known association with the outcome of interest—to arrive at the best possible estimate of the outcome of interest when data for the outcome are scarce but data for covariates are available.^1^, ^2^, ^3^, ^25^, ^26^ This approach was part of the estimation process for the findings presented in this Article.

### Analysis presented in this paper

We report findings for 31 geographical units in India, comprising 29 states, the Union Territory of Delhi, and union territories other than Delhi (combining the six smaller union territories of Andaman and Nicobar Islands, Chandigarh, Dadra and Nagar Haveli, Daman and Diu, Lakshadweep, and Puducherry). The states of Chhattisgarh, Uttarakhand, and Jharkhand were created from existing larger states in 2000, and the state of Telangana was created in 2014. For trends from 1990 onward, we disaggregated data for these four new states from their parent states, based on data from the districts that now constitute these states. We also presented findings based on epidemiological transition level (ETL), as described previously.^23^ Briefly, we defined ETL state groups based on the ratio of DALYs from communicable, maternal, neonatal, and nutritional diseases to those from non-communicable diseases and injuries combined in 2016, with a relatively lower ratio indicating higher ETL: low ETL (ratio 0·56–0·75), lower-middle ETL (0·41–0·55), higher-middle ETL (0·31–0·40), and high ETL (<0·31). We have reported previously that epidemiological transition ratios of the states of India have a significant inverse relation with the Socio-demographic Index calculated by GBD 2016 based on income, education, and fertility levels, which indicates broad correspondence of ETL state groups with sociodemographic development levels.^23^

We present differences in the prevalence of, and deaths and DALYs due to cardiovascular diseases and the major component causes, ischaemic heart disease and stroke, in addition to rheumatic heart disease—a subcause that is particularly of interest in India—from 1990 to 2016. We report estimates of ischaemic heart disease and stroke DALYs attributable to major risk factors in 2016. Risk factors included dietary risks, high systolic blood pressure, high total blood cholesterol, tobacco use, high fasting plasma glucose, high body-mass index (BMI), and air pollution. Dietary risks consist of ten components that are protective (eg, intake of fruits, nuts and seeds, vegetables, and wholegrains) and five components that are harmful (eg, intake of sodium, trans-fatty acids, and red meat; appendix pp 33–36). We present changes in the prevalence of high systolic blood pressure (≥140 mm Hg) in adults aged 30 years or older, high total blood cholesterol (≥200 mg/dL [≥11·1 mmol/L]) in adults aged 30 years or older, high fasting plasma glucose (≥126 mg/dL [≥7·0 mmol/L]) in adults aged 20 years or older, and smoking (current use of any smoked tobacco product) in people aged 10 years or older for India and the four ETL state groups, between 1990 and 2016. We compared age-standardised DALY rates for cardiovascular diseases and the major component causes in 2016 in India with the global average.^30^

We present both crude and age-standardised estimates, as relevant. Crude estimates provide the actual situation in each state, which is useful for policy makers, and age-standardised estimates allow comparisons over time and between states after adjusting for differences in the age structure of the population. Age-standardised rates were based on the GBD global reference population.^1^ We report estimates with 95% uncertainty intervals (UIs) when relevant. UIs were based on 1000 runs of the models for each quantity of interest, with the mean regarded as the point estimate and the 2·5th and 97·5th percentiles considered the 95% UI (appendix p 41).^1^, ^2^, ^3^, ^25^, ^26^

### Role of the funding source

Some coauthors are employees of the Indian Council of Medical Research and contributed to various aspects of the study and this analysis. The other funder of the study had no role in study design, data collection, data analysis, data interpretation, or writing of the report. The corresponding author had full access to all data in the study and had final responsibility for the decision to submit for publication.

## Results

Cardiovascular diseases contributed to 28·1% (95% UI 26·5–29·1) of total deaths and 14·1% (12·9–15·3) of total DALYs in India in 2016 (table 1)—compared with 15·2% (13·7–16·2) and 6·9% (6·3–7·4), respectively, in 1990.^31^ Ischaemic heart disease and stroke were the predominant cardiovascular diseases, contributing to 61·4% and 24·9% of total DALYs from cardiovascular diseases, respectively, in 2016. Ischaemic heart disease was the leading cause of DALYs in India in 2016, and stroke the fifth leading cause.^23^, ^24^ Ischaemic heart disease contributed 17·8% (95% UI 16·8–18·5) of total deaths and 8·7% (7·9–9·5) of total DALYs, and stroke contributed 7·1% (6·6–7·5) of total deaths and 3·5% (3·2–3·9) of total DALYs (table 1). The proportion of deaths and DALYs from ischaemic heart disease was significantly higher in men than in women, but were similar in the two sexes for stroke (table 1).

**Table 1:** Percentage of total deaths and DALYs due to each cause under cardiovascular diseases by sex in India, 2016

		**Percentage of total deaths (95% UI)**			**Percentage of total DALYs (95% UI)**		
		Both sexes	Men	Women	Both sexes	Men	Women
Cardiovascular diseases		28·1% (26·5–29·1)	29·2% (27·5–30·3)	26·7% (23·8–28·3)	14·1% (12·9–15·3)	15·8% (14·5–17·1)	12·2% (10·9–13·4)
	Ischaemic heart disease	17·8% (16·8–18·5)	19·6% (18·5–20·4)	15·6% (13·9–16·6)	8·7% (7·9–9·5)	10·4% (9·5–11·3)	6·6% (5·9–7·4)
	Stroke	7·1% (6·6–7·5)	6·9% (6·4–7·3)	7·3% (6·5–7·9)	3·5% (3·2–3·9)	3·6% (3·3–4·0)	3·4% (3·0–3·8)
	Hypertensive heart disease	1·3% (1·1–1·5)	1·1% (0·9–1·4)	1·6% (1·2–1·9)	0·6% (0·5–0·7)	0·6% (0·5–0·7)	0·7% (0·5–0·8)
	Rheumatic heart disease	1·1% (1·0–1·2)	0·8% (0·7–0·9)	1·5% (1·3–1·7)	0·8% (0·7–0·9)	0·7% (0·6–0·7)	1·0% (0·9–1·1)
	Atrial fibrillation and flutter	0·21% (0·16–0·26)	0·17% (0·13–0·21)	0·25% (0·20–0·32)	0·13% (0·11–0·16)	0·13% (0·10–0·15)	0·15% (0·12–0·18)
	Aortic aneurysm	0·15% (0·14–0·17)	0·20% (0·18–0·21)	0·10% (0·09–0·11)	0·07% (0·07–0·08)	0·10% (0·09–0·11)	0·05% (0·04–0·06)
	Other cardiovascular and circulatory diseases	0·14% (0·09–0·17)	0·13% (0·07–0·18)	0·15% (0·08–0·18)	0·07% (0·05–0·08)	0·07% (0·05–0·09)	0·07% (0·05–0·08)
	Cardiomyopathy and myocarditis	0·12% (0·09–0·13)	0·12% (0·09–0·15)	0·11% (0·07–0·13)	0·11% (0·08–0·12)	0·11% (0·08–0·13)	0·10% (0·06–0·12)
	Endocarditis	0·12% (0·10–0·15)	0·11% (0·09–0·16)	0·14% (0·11–0·18)	0·07% (0·06–0·09)	0·07% (0·06–0·10)	0·08% (0·06–0·10)
	Peripheral artery disease	0·01% (0·07–0·03)	0·02% (0·01–0·04)	0·01% (0·01–0·02)	0·02% (0·01–0·03)	0·02% (0·01–0·03)	0·02% (0·01–0·03)

DALY=disability-adjusted life-year. UI=uncertainty interval.

**Figure 1:**
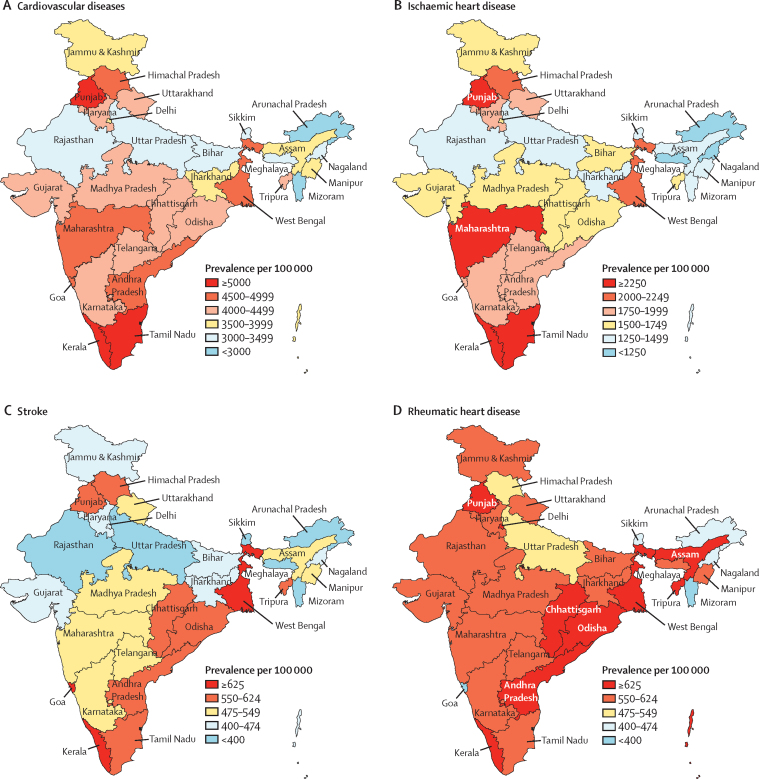
Crude prevalence of cardiovascular diseases and major component causes in the states of India, 2016

Prevalent cases of cardiovascular diseases increased in India from 25·7 million (95% UI 25·1–26·0) in 1990 to 54·5 million (53·7–55·3) in 2016. The prevalence of cardiovascular diseases in 2016 was highest in Kerala, Punjab, and Tamil Nadu (which are in the high ETL state group), followed by Andhra Pradesh, Himachal Pradesh, Maharashtra, Goa, and West Bengal (which are in the high and higher-middle ETL state groups; figure 1).

The number of cases of ischaemic heart disease increased from 10·2 million (95% UI 9·8–10·6) in 1990 to 23·8 million (22·6–25·0) in 2016, and cases of stroke from 2·8 million (95% UI 2·7–2·9) in 1990 to 6·5 million (6·3–6·8) in 2016.^31^ The crude prevalence of ischaemic heart disease and stroke increased substantially across all ETL state groups and all states from 1990 to 2016, and the age-standardised prevalence increased modestly (appendix pp 58, 59). The age-standardised prevalence of rheumatic heart disease decreased in India by 10·8% (95% UI 8·7–13·0) from 1990 to 2016; this prevalence decreased across all ETL state groups (appendix p 58).

The prevalence of ischaemic heart disease in 2016 had an increasing gradient from the low to the high ETL state groups, with the highest prevalence in Kerala, Punjab, Tamil Nadu (high ETL state group), and Maharashtra (higher-middle ETL state group; figure 1). The prevalence of stroke in 2016 did not have a clear gradient across the ETL state groups, with the highest prevalence in West Bengal (higher-middle ETL state group) and in Kerala and Goa (high ETL state group). The prevalence of rheumatic heart disease in 2016 was similar across the ETL state groups, with the highest prevalence in several states spread across the country (figure 1).

Deaths due to cardiovascular diseases in India increased from 1·3 million (95% UI 1·2–1·4) in 1990 to 2·8 million (2·6–2·9) in 2016. The crude death rate of cardiovascular diseases increased by 34·3% (95% UI 26·6–43·7) from 1990 to 2016 in India, but the age-standardised rate did not change significantly during this period (appendix p 57). The crude death rate from cardiovascular diseases increased in both sexes from 1990 to 2016 in all ETL state groups, and the age-standardised rate increased in men in the low ETL state group (appendix p 57). The death rate from cardiovascular diseases in India in 2016 was higher among adults aged 70 years or older than in those younger than 70 years (2777 per 100 000 [95% UI 2550–2922] *vs* 116 per 100 000 [110–120]), but the proportion of total deaths from cardiovascular diseases was higher among people younger than 70 years than in those aged 70 years or older (53·4% [95% UI 52·6–54·6] *vs* 46·6 [45·4–47·4]; table 2). The proportion of deaths from cardiovascular diseases in people younger than 70 years was highest in the low ETL state group (table 2).

**Table 2:** Deaths from cardiovascular diseases at age less than 70 years versus older age in the states of India grouped by ETL, 2016

	**Deaths per 100 000 at age <70 years (95% UI)**	**Percentage of total deaths at age <70 years (95% UI)**	**Deaths per 100 000 at age ≥70 years (95% UI)**	**Percentage of total deaths at age ≥70 years (95% UI)**
Low ETL (626 million)*	97 (91–104)	55·6% (54·4–57·4)	2442 (2139–2650)	44·4% (42·6–45·6)
Lower-middle ETL (92 million)	111 (101–121)	53·4% (51·9–54·9)	2808 (2575–3033)	46·6% (45·1–48·1)
Higher-middle ETL (446 million)	135 (126–143)	53·1% (52·3–54·2)	3072 (2846–3263)	46·9% (45·8–47·7)
High ETL (152 million)	141 (129–152)	48·4% (47·3–49·8)	2979 (2725–3176)	51·6% (50·2–52·7)
India (1316 million)	116 (110–120)	53·4% (52·6–54·6)	2777 (2550–2922)	46·6% (45·4–47·4)

ETL=epidemiological transition level. UI=uncertainty interval.

* Population in 2016 given in parentheses.

**Figure 2:**
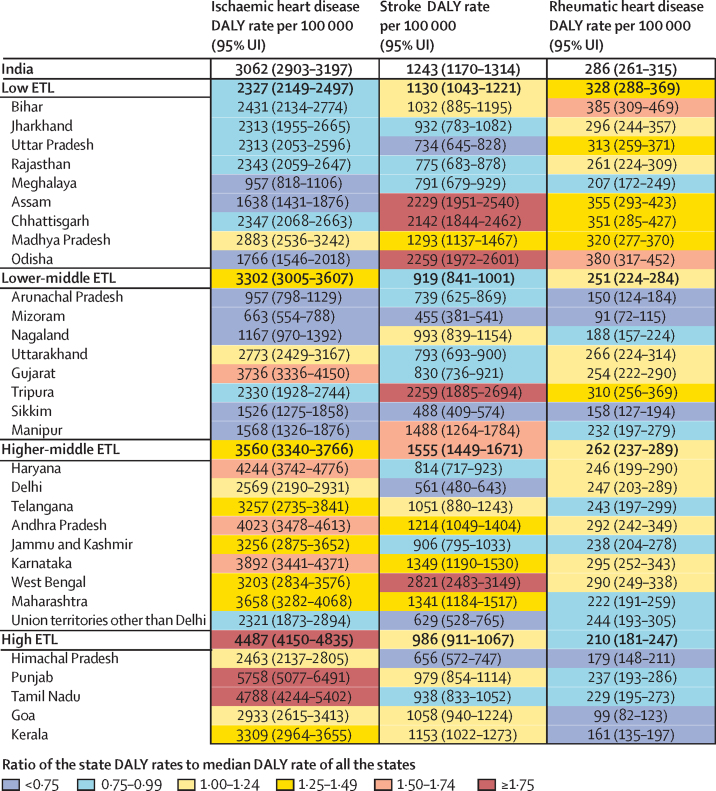
Crude DALY rates of ischaemic heart disease, stroke, and rheumatic heart disease in the states of India, 2016 DALY=disability-adjusted life-year. ETL=epidemiological transition level. UI=uncertainty interval.

The DALY rate of ischaemic heart disease varied 8·7 times between the states 2016 (figure 2). This rate was highest in the states Punjab and Tamil Nadu, which are in the high ETL state group, followed by Haryana, Andhra Pradesh, Karnataka (higher-middle ETL), and Gujarat (lower-middle ETL). The DALY rate of stroke varied 6·2 times between the states in 2016, and was highest in the higher-middle ETL state group (figure 2). The states with the highest rate of stroke DALYs were West Bengal and Odisha in the east, Tripura and Assam in the northeast, and Chhattisgarh in central India. The DALY rate of rheumatic heart disease varied 4·2 times between the states in 2016, and was highest in Bihar and Odisha, which are in the low ETL state group, and was lowest in the high ETL state group.

**Figure 3:**
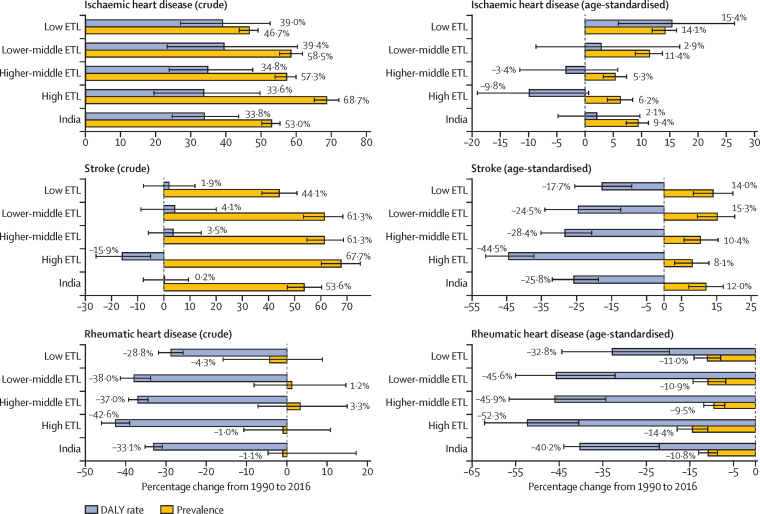
Comparison between the relative change from 1990 to 2016 in prevalence and DALY rates of ischaemic heart disease, stroke, and rheumatic heart disease in the states of India, grouped by ETL Error bars are 95% uncertainty intervals. DALY=disability-adjusted life-year. ETL=epidemiological transition level.

The crude DALY rate of ischaemic heart disease increased by 33·8% (95% UI 24·7–43·6) from 1990 to 2016 in India, compared with a 53·0% (50·2–55·4) increase in prevalence (figure 3). The age-standardised DALY rate due to ischaemic heart disease did not change significantly during this period (2·1%, 95% UI −4·8 to 9·7), but prevalence increased by 9·4% (7·3 to 11·2). The age-standardised DALY rate of ischaemic heart disease increased significantly by 15·4% (95% UI 5·9–26·4) in the low ETL state group, and age-standardised prevalence also increased significantly by 14·1% (11·9–16·2). However, the age-standardised DALY rate due to ischaemic heart disease decreased in the high ETL state group (9·8%, 95% UI −19·1 to 0·6), but the prevalence increased (6·2%, 3·9 to 8·4). The crude DALY rate due to stroke did not change from 1990 to 2016 in India (figure 3), but the age-standardised rate decreased by 25·8% (95% UI 18·8–32·0), with the highest decline in the high ETL state group (44·5%, 95% UI 37·3–51·1). This change contrasts with the increase in age-standardised prevalence of stroke in all ETL state groups. The age-standardised DALY rate of rheumatic heart disease decreased in all ETL state groups from 1990 to 2016; this percentage change was more than the decrease in age-standardised prevalence of rheumatic heart disease during this period (figure 3).

The age-standardised DALY rate due to cardiovascular diseases in India in 2016 was 1·3 times the global average, that of ischaemic heart disease was 1·6 times the global average, rheumatic heart disease was 2·4 times the global average, and the age-standardised DALY rate due to stroke were comparable to the global average.^30^ With 17·8% of the global population in 2016, India had 23·1%, 14·0%, and 37·6% of global DALYs due to ischaemic heart disease, stroke, and rheumatic heart disease, respectively.

**Figure 4:**
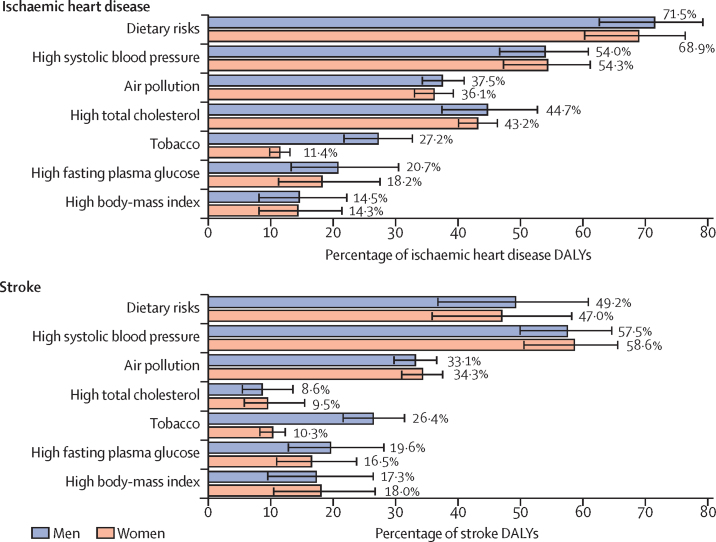
Percentage contribution of major risk factors to ischaemic heart disease and stroke DALYs in India by sex, 2016 Error bars represent 95% uncertainty intervals. DALY=disability-adjusted life-year.

Among the risk factors that contributed to DALYs due to cardiovascular diseases in India in 2016, the leading ones were dietary risks (56·4%, 95% UI 48·5–63·9), high systolic blood pressure (54·6%, 49·0–59·8), air pollution (31·1%, 29·0–33·4), high total cholesterol (29·4%, 24·3–34·8), tobacco use (18·9, 16·6–21·3), high fasting plasma glucose (16·7%, 11·4–23·5), and high BMI (14·7%, 8·3–22·0), for both sexes combined (appendix p 60). Of the cardiovascular disease DALYs attributable to tobacco use, most were due to smoking (83·4%). It is important to note that the cumulative effect of these risk factors is less than the simple addition of their individual contribution, because the risk factors overlap. Furthermore, population attributable fractions from components can add up to more than their sum, even if they are independent. Leading risk factors for DALYs attributable to ischaemic heart disease were dietary risks, high systolic blood pressure, high total cholesterol, and air pollution; for stroke DALYs, risk factors were high systolic blood pressure, dietary risks, and air pollution (figure 4). The proportion of ischaemic heart disease DALYs attributable to high total cholesterol was much greater than for stroke. The proportional contribution of tobacco use to ischaemic heart disease and stroke DALYs was larger in men than in women.

**Figure 5:**
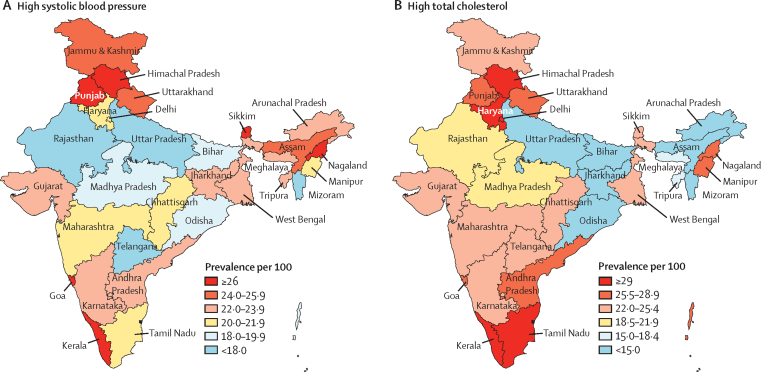
Prevalence of high systolic blood pressure and high total cholesterol in adults aged 30 years or older in the states of India, 2016

The crude prevalence of high systolic blood pressure, high total cholesterol, and high fasting plasma glucose generally increased in India and across all ETL state groups from 1990 to 2016, but the prevalence of smoking decreased (table 3). The prevalence of high systolic blood pressure, high total cholesterol, and high fasting plasma glucose in 2016 was highest in the high ETL state group. Among the states of India, the prevalence of high systolic blood pressure was highest in Punjab, Himachal Pradesh, Kerala, and Goa in the high ETL state group and in Sikkim and Nagaland in the lower-middle ETL state group, with two times variation across the states (figure 5). The prevalence of high total cholesterol was highest in Kerala, Himachal Pradesh, and Tamil Nadu in the high ETL state group, and Haryana and Delhi in the higher-middle ETL state group, with 6·5 times variation across the states. The age-standardised prevalence of high fasting plasma glucose increased in all ETL state groups from 1990, but the increase was highest in low and lower-middle ETL state groups (table 3). The prevalence of high BMI increased in every state of India from 1990 to 2016, as described by us in a companion paper in *The Lancet Global Health*.^32^ The exposure to ambient air pollution also increased in India to varying degrees in the different states from 1990 to 2016.^23^, ^24^, ^31^

**Table 3:** Change in prevalence of high systolic blood pressure, high total cholesterol, high fasting plasma glucose, and smoking in the states of India grouped by ETL, 1990 to 2016

	**Both sexes**			**Men**			**Women**		
	Crude prevalence per 100* (95% UI)		Age-standardised percentage change, 1990 to 2016 (95% UI)	Crude prevalence per 100* (95% UI)		Age-standardised percentage change, 1990 to 2016 (95% UI)	Crude prevalence per 100* (95% UI)		Age-standardised percentage change, 1990 to 2016 (95% UI)
	2016	Percentage change 1990 to 2016		2016	Percentage change 1990 to 2016		2016	Percentage change 1990 to 2016	
**Low ETL**									
High systolic blood pressure	18·8 (18·5 to 19·0)	0·1 (−2·6 to 2·7)	−4·1 (−6·4 to −1·9)	18·3 (18·0 to 18·7)	7·4 (3·6 to 11·6)	3·4 (0·2 to 7·1)	19·2 (18·9 to 19·6)	−6·4 (−10·0 to −2·7)	−10·7 (−13·7 to −7·5)
High total cholesterol	17·4 (16·8 to 18·1)	11·0 (5·0 to 17·8)	11·0 (5·4 to 17·6)	16·6 (15·7 to 17·5)	8·8 (0·0 to 18·7)	9·0 (0·9 to 18·1)	18·3 (17·3 to 19·2)	13·2 (4·1 to 22·9)	12·5 (4·2 to 21·7)
High fasting plasma glucose	6·6 (5·9 to 7·3)	42·5 (38·0 to 47·2)	35·8 (31·6 to 39·9)	7·3 (6·5 to 8·2)	46·3 (40·2 to 52·3)	40·4 (34·8 to 46·0)	5·9 (5·3 to 6·5)	37·8 (32·8 to 42·5)	30·4 (26·0 to 34·6)
Smoking	8·5 (8·1 to 9·0)	−33·8 (−40·0 to −27·3)	−36·2 (−42·1 to −30)	14·3 (13·5 to 15·2)	−33·9 (−39·8 to −27·4)	−35·8 (−41·6 to −29·6)	2·2 (1·8 to 2·8)	−32·7 (−53·8 to −4·4)	−37·2 (−57·1 to −10·8)
**Lower–middle ETL**									
High systolic blood pressure	22·7 (22·1 to 23·2)	12·2 (7·0 to 17·5)	5·6 (1·2 to 10·0)	23·2 (22·5 to 23·9)	20·8 (13·6 to 28·6)	14·5 (8·7 to 20·8)	22·2 (21·4 to 23·0)	4·1 (−2·6 to 11·2)	−2·2 (−8·1 to 4·3)
High total cholesterol	24·9 (23·4 to 26·1)	20·6 (9·2 to 32·5)	18·2 (8·4 to 28·5)	25·3 (23·2 to 27·7)	22·7 (6·1 to 41·7)	22·3 (8·2 to 39·7)	24·5 (22·7 to 26·4)	18·3 (3·2 to 35·1)	14·2 (0·5 to 29·1)
High fasting plasma glucose	7·0 (6·3 to 7·6)	43·9 (37·6 to 50·1)	33·6 (27·9 to 40·0)	7·4 (6·7 to 8·1)	47·0 (38·7 to 55·7)	37·1 (29·8 to 45·5)	6·5 (5·8 to 7·2)	40·4 (33·3 to 47·1)	29·8 (23·0 to 36·2)
Smoking	9·8 (9·1 to 10·5)	−16·6 (−25·3 to −6·5)	−24·9 (−33·1 to −15·7)	17·3 (16 to 18·7)	−16·6 (−25·5 to −6·1)	−24·3 (−32·9 to −14·1)	1·6 (1·2 to 2)	−23·9 (−49·2 to 12·0)	−30·5 (−53·3 to 1·0)
**Higher–middle ETL**									
High systolic blood pressure	21·6 (21·3 to 21·9)	16·4 (13·1 to 19·7)	7·3 (4·6 to 10·0)	21·3 (20·8 to 21·8)	28·7 (23·6 to 33·8)	19·4 (15·2 to 23·4)	21·9 (21·5 to 22·4)	5·8 (1·8 to 10·0)	−2·5 (−5·9 to 0·9)
High total cholesterol	25·2 (24·4 to 26·1)	21·6 (15·3 to 28·1)	18·8 (13·1 to 24·4)	23·3 (22·1 to 24·6)	21·3 (12·5 to 31·2)	19·9 (11·9 to 29·1)	27·2 (25·9 to 28·6)	21·6 (13·2 to 30·8)	17·5 (9·9 to 25·7)
High fasting plasma glucose	7·5 (6·8 to 8·3)	36·4 (32·5 to 40·5)	25·4 (21·8 to 28·8)	8·4 (7·6 to 9·2)	37·7 (32·8 to 42·5)	27·9 (23·4 to 32·2)	6·6 (5·9 to 7·4)	35·3 (30·6 to 40·0)	23·1 (19·1 to 29·8)
Smoking	9·2 (8·7 to 9·7)	−26·8 (−31·9 to −20·7)	−34·1 (−38·7 to −28·7)	16·5 (15·7 to 17·3)	−26·8 (−32·1 to −20·8)	−33·6 (−38·3 to −28·2)	1·4 (1·2 to 1·7)	−21·1 (−42·1 to 7·1)	−31·1 (−48·8 to −6·8)
**High ETL**									
High systolic blood pressure	26·0 (25·5 to 26·4)	22·2 (18·2 to 26·1)	12·5 (9·1 to 15·7)	27·1 (26·4 to 27·7)	34·7 (28·7 to 41·4)	25·5 (20·5 to 31·0)	24·9 (24·2 to 25·6)	11·1 (5·6 to 16·6)	1·2 (−3·3 to 5·6)
High total cholesterol	33·7 (32·6 to 34·8)	24·5 (16·8 to 32·2)	22·4 (15·8 to 29·5)	30·9 (29·2 to 32·6)	23·6 (12·9 to 34·8)	24·7 (14·9 to 35·2)	36·4 (34·9 to 38·0)	24·8 (14·7 to 35·3)	20·0 (11·0 to 29·3)
High fasting plasma glucose	11·8 (10·9 to 12·8)	43·6 (38·4 to 49·1)	26·9 (22·5 to 31·6)	12·1 (11·2 to 13·0)	47·5 (40·9 to 54·7)	31·5 (25·5 to 37·4)	11·6 (10·6 to 12·6)	39·7 (34·2 to 46·1)	22·6 (17·9 to 27·7)
Smoking	6·7 (6·2 to 7·2)	−43·9 (−50·1 to −37·2)	−49·9 (−55·1 to −44·1)	12·8 (11·7 to 13·7)	−43·7 (−50·2 to −36·8)	−49·3 (−54·8 to −43·2)	0·6 (0·5 to 0·8)	−43·1 (−63·9 to −12·4)	−47·4 (−64·8 to −21·1)
**India**									
High systolic blood pressure	21·1 (20·9 to 21·2)	10·0 (8·3 to 11·7)	3·3 (1·8 to 4·7)	20·9 (20·7 to 21·1)	19·8 (17·0 to 22·4)	13·3 (11·0 to 15·4)	21·2 (21·0 to 21·5)	1·4 (−1·0 to 3·6)	−5·2 (−7·1 to −3·4)
High total cholesterol	23·0 (22·6 to 23·6)	18·2 (14·9 to 22·2)	16·8 (13·6 to 20·3)	21·6 (20·9 to 22·2)	17·0 (11·9 to 23·0)	16·9 (12·2 to 22·3)	24·5 (23·8 to 25·2)	19·1 (14·0 to 24·4)	16·2 (11·5 to 21·1)
High fasting plasma glucose	7·7 (6·9 to 8·4)	39·4 (35·7 to 43·3)	29·7 (26·5 to 32·6)	8·3 (7·5 to 9·1)	42·1 (37·9 to 46·8)	33·5 (29·5 to 37·5)	7·0 (6·3 to 7·7)	36·2 (32·3 to 39·9)	25·6 (22·6 to 28·7)
Smoking	8·6 (8·3 to 8·9)	−31·3 (−35 to −27·6)	−36·3 (−39·7 to −33·0)	15·1 (14·6 to 15·6)	−31·5 (−34·8 to −27·8)	−36 (−39 to −32·6)	1·7 (1·5 to 2·0)	−28·3 (−43·6 to −10·1)	−35·8 (−50 to −19·9)

ETL=epidemiological transition level. UI=uncertainty interval.

* Prevalence of high systolic blood pressure and high total cholesterol among adults aged 30 years or older, prevalence of high fasting plasma glucose among adults aged 20 years or older, and prevalence of smoking among people aged 10 years or older.

## Discussion

The findings in this report provide a systematic understanding of the burden due to cardiovascular diseases and their risk factors in every state of India from 1990 to 2016, using all available data sources that could be accessed. The contribution of cardiovascular diseases to total deaths and disease burden in India has almost doubled since 1990. Ischaemic heart disease was the leading individual cause of disease burden in India in 2016, and stroke was the fifth leading cause.^23^, ^24^

The crude prevalence of ischaemic heart disease and stroke has increased in every state of India since 1990, and the age-standardised prevalence has increased in most of the states. Although the highest prevalence of ischaemic heart disease and stroke in 2016 was in the high ETL state groups, the increase in the age-standardised prevalence of ischaemic heart disease since 1990 was higher in the low and lower-middle ETL state groups, which include many less developed states that are home to 55% of India's population. More than half of the total cardiovascular disease deaths in India in 2016 were in people younger than 70 years. This proportion was highest in the low ETL state group, which is a major cause for concern with respect to the challenges posed to the health systems in these relatively less developed states. Reducing premature deaths from cardiovascular diseases in the economically productive age groups requires urgent action across all states of India.

The burden of cardiovascular diseases in 2016 varied strikingly between the states of India, with a nine times variation in the DALY rate of ischaemic heart disease, a six times variation in the DALY rate due to stroke, and a four times variation in the DALY rate due to rheumatic heart disease. The increase in crude prevalence and age-standardised prevalence of ischaemic heart disease and stroke across all ETL state groups since 1990 can be attributed to the ageing of the population and the increasing effect of risk factors. The age-standardised prevalence of ischaemic heart disease increased in all ETL state groups, with the highest increase in the less developed low ETL state group, while age-standardised DALY rate increased only in the low ETL state group. The age-standardised prevalence of stroke increased in all ETL state groups during this period, and age-standardised DALY rate declined in all ETL state groups, but the smallest decrease was in the low ETL state group. Although falling rates of age-standardised DALYs reflect improving health care in general, the increase in the DALY rate of ischaemic heart disease and the relatively lower improvements in rates of DALYs due to stroke in resource-poor low ETL states indicate larger gaps in the prevention and care of cardiovascular diseases in these states.^19^, ^33^, ^34^ The age-standardised prevalence of rheumatic heart disease decreased from 1990 to 2016 in all ETL state groups, and the age-standardised DALY rate due to rheumatic heart disease declined even more than the decrease in prevalence. The DALY rate of rheumatic heart disease in 2016 was highest in the relatively less developed low ETL state group. Variations in cardiovascular disease burden seen between states reflects the different stages of epidemiological transition of cardiovascular diseases. In 2016, the DALY rates of ischaemic heart disease were generally higher in the more advanced high ETL state group, the DALY rate of stroke had a mixed pattern across the ETL state groups, and the DALY rates of rheumatic heart disease were higher in the low ETL state group. The variable relation of these diseases with socioeconomic development has been noted previously.^7^, ^20^, ^35^, ^36^

The National Programme for Prevention and Control of Cancer, Diabetes, Cardiovascular Diseases and Stroke (NPCDCS), which was launched in 2010, aims to prevent and control disease burden through early screening, behavioural change, and capacity building for human resources and infrastructure.^37^ Although the NPCDCS has established NCD units in all states and union territories as of March, 2017, implementation of these and other efforts across the states of India needs more time to show progress towards achieving national and global targets for NCDs, including cardiovascular diseases and its risk factors.

The Indian Government announced in early 2018 Ayushman Bharat, the National Health Protection Mission.^38^ A major component of this programme is a health insurance scheme that aims to cover 500 million people from poor and vulnerable families in India.^38^ Since the increasing burden of NCDs places further economic pressure on individuals and households,^39^ Ayushman Bharat has the potential to reduce the financial burden from NCDs in India. Ayushman Bharat includes the establishment of 150 000 Health and Wellness Centres across India to provide comprehensive primary health-care services that are commensurate with the leading causes of disease burden, including from cardiovascular diseases and other NCDs.^40^ Each Indian state will need to contextualise prevention and management strategies for cardiovascular diseases according to the magnitude of its burden and trends. Such efforts can be informed by the findings for each state of India presented in this Article.

The prevalence of most major risk factors that contribute to cardiovascular disease burden—including high systolic blood pressure, ambient air pollution, high total cholesterol, high fasting plasma glucose, and high BMI—has increased across India since 1990, although the current prevalence varies greatly between ETL state groups and individual states. In some studies,^18^, ^41^ a higher prevalence of hypertension in India has been reported, compared with our estimates, which could be due to inclusion of diastolic blood pressure in the case-definition, or other study design differences. Underdiagnosis of high systolic blood pressure and high total cholesterol, and inadequate access to medications for these disorders, is common in India, which contributes to their increasing prevalence.^42^, ^43^ Metabolic risks—eg, high systolic blood pressure, high total cholesterol, high fasting plasma glucose, and high BMI—are related to dietary consumption. Efforts have been ongoing to address some of the dietary risks related to consumption of sodium, trans fatty acids, and sugar-sweetened beverages. The proposed fat tax and advertisement ban on foods high in fat, sugar, and salt by the Food Safety and Standards Authority of India in 2017 would be a step towards reducing some of these risks that contribute to the burden of cardiovascular diseases.^44^ Smoking was the only major cardiovascular disease risk factor that decreased in prevalence from 1990, suggesting that implementation of the Cigarettes and Other Tobacco Products Act in 2003 to discourage use of tobacco products, and the National Tobacco Control Programme launched in 2007, may be facilitating a reduction in tobacco use, but sustained efforts are needed for further progress.^45^, ^46^ Ongoing initiatives to tackle the burden of air pollution in India, which is a major contributor to ischaemic heart disease and stroke, and the challenges in controlling this risk factor have been noted in earlier publications by the India State-Level Disease Burden Initiative.^23^, ^24^ The increasing prevalence of various lifestyle risk factors and environmental risks contributing to cardiovascular diseases across India is ominous, and this situation has to be addressed through systematic policies and action in various sectors.^5^, ^47^

General limitations of the GBD 2016 methodology, and those for estimation of cardiovascular diseases and related risk factors, are described elsewhere.^1^, ^2^, ^3^, ^25^, ^26^, ^27^, ^29^, ^42^ A specific limitation for India is an incomplete medically certified cause of death system, which currently covers only a small proportion of deaths in India. Verbal autopsy cause of death data from the Sample Registration System were a useful alternative for the findings presented in this report.^23^ However, India needs to improve the coverage and quality of its cause of death assignment system. Population-based data for morbidity due to cardiovascular diseases and their sequelae are generally not available for many parts of India, although several ongoing studies are attempting to address this gap. A particular challenge is scarce data to distinguish between the distribution of ischaemic and haemorrhagic stroke at the population level. Because we report the prevalence of high systolic blood pressure, and not diastolic blood pressure, this prevalence would be lower than that for hypertension defined using both systolic and diastolic blood pressure levels. Data for some risk factors (eg, dietary risks) in urban populations across the states of India that had been sparse previously have recently become available, which would strengthen the estimates further. Additional population-level data for the burden of cardiovascular diseases attributable to risk factors in different parts of India would also make the estimates more robust. Adequate population-level disease registries and surveillance systems to monitor morbidity trends of the major cardiovascular diseases and their risk factors are needed in India.

The strengths of our study include the use of all available data sources in India that could be accessed to estimate trends in the burden of cardiovascular diseases and their risk factors in every state of India over a period of 26 years. Nationwide health surveys over the past few years in India have provided useful data for many cardiovascular disease risk factors in all states of India. When data are scarce for a particular variable, GBD uses covariates and other techniques that borrow strength over space and time to arrive at the best possible estimates. Other strengths include use of the standardised GBD methodology for comparison across locations and years, and a comprehensive effort that benefited from the inputs of a network of leading experts in India.

This Article documents cardiovascular disease trends in each state of India over a quarter of a century, highlighting that cardiovascular diseases are the largest contributor to disease burden of any disease group and are a major public health problem leading to premature deaths and morbidity across all states of India. The increasing burden of cardiovascular diseases and its risk factors needs to be addressed urgently. Although there have been impressive advances in the capacity for preventing and treating cardiovascular disease globally, these need much enhancement across all of India, with particular attention to the relatively less developed states where the rate of increase in the cardiovascular disease burden is among the highest in the country. The state-specific findings in this report can serve as a useful reference for informing policies and programmes to plan more effectively the prevention and treatment of cardiovascular diseases in each state of India, which will facilitate progress towards achieving national and global targets for cardiovascular disease reduction.
