# Supplementary material for: The changing patterns of cardiovascular diseases and their risk factors in the states of India: the Global Burden of Disease Study 1990–2016
Source: Lancet Glob Health. 2018 Sep 12;6(12):e1339–51. doi: 10.1016/S2214-109X(18)30407-8 (PMC6227386; doi:10.1016/S2214-109X(18)30407-8)
Supplement: Supplementary appendix [file mmc1.pdf]

THE LANCET
Global Health

Supplementary appendix

This appendix formed part of the original submission and has been peer reviewed. We post it as supplied by the authors.

Supplement to: India State-Level Disease Burden Initiative CVD Collaborators. The changing patterns of cardiovascular diseases and their risk factors in the states of India: the Global Burden of Disease Study 1990–2016. *Lancet Glob Health* 2018; published online Sept 12. http://dx.doi.org/10.1016/S2214-109X(18)30407-8.

The changing patterns of cardiovascular diseases and their risk factors in the states of India: the Global Burden of Disease Study 1990–2016

India State-Level Disease Burden Initiative CVD Collaborators

Web Appendix

Correspondence to: Prof. Lalit Dandona, lalit.dandona@phfi.org

Table of Contents

1. GBD 2016 cardiovascular diseases burden estimation methods 3
2. GBD 2016 India data inputs for cardiovascular diseases mortality, morbidity, risk factors, and covariates .. 42
3. Change in death and DALY rates due to cardiovascular diseases by sex in the states of India grouped by epidemiological transition level, 1990 to 2016 57
4. Change in prevalence of IHD, stroke, and RHD in the states of India grouped by epidemiological transition level, 1990 to 2016 58
5. Change in prevalence of IHD and stroke in the states of India, 1990 to 2016 59
6. Percent contribution of major risk factors to cardiovascular diseases DALYs in India by sex, 2016 60

1. GBD 2016 cardiovascular diseases burden estimation methods

The material presented here is adapted from the following sources:

- GBD 2016 Disease and Injury Incidence and Prevalence Collaborators. Global, regional, and national incidence, prevalence, and years lived with disability for 328 diseases and injuries for 195 countries, 1990–2016: a systematic analysis for the Global Burden of Disease Study 2016. Lancet 2017; 390: 1211–59.
- GBD 2016 Causes of Death Collaborators. Global, regional, and national age-sex specific mortality for 264 causes of death, 1980–2016: a systematic analysis for the Global Burden of Disease Study 2016. Lancet 2017; 390: 1151–210.
- GBD 2016 Risk Factors Collaborators. Global, regional, and national comparative risk assessment of 84 behavioural, environmental and occupational, and metabolic risks or clusters of risks, 1990–2016: a systematic analysis for the Global Burden of Disease Study 2016. Lancet 2017; 390: 1345–422.
- Cohen AJ, Brauer M, Burnett R, et al. Estimates and 25-year trends of the global burden of disease attributable to ambient air pollution: an analysis of data from the Global Burden of Diseases Study 2015. Lancet 2017; 389: 1907–18.

The GBD cause list is organised hierarchically into four levels. At each level of the hierarchy, the set of causes is mutually exclusive and collectively exhaustive. Levels 1 and 2 represent general groupings. The broad group "cardiovascular diseases" is at level 2 under the level 1 group "non-communicable diseases". Level 3 includes ten cardiovascular diseases (CVDs) groups which are: rheumatic heart disease, ischaemic heart disease, stroke, hypertensive heart disease, cardiomyopathy and myocarditis, atrial fibrillation and flutter, aortic aneurysm, peripheral arterial disease, endocarditis, and other cardiovascular and circulatory diseases. Level 4 includes five groups, under the parent level 3 "stroke" group: ischaemic stroke and haemorrhagic stroke, and level 3 "cardiomyopathy and myocarditis" group: myocarditis, alcoholic cardiomyopathy, and other cardiomyopathy.

A. GBD case definitions of cardiovascular diseases and inclusions

The GBD case definitions and diagnostic criteria for the cardiovascular diseases are presented below.

Ischaemic heart disease (IHD)

1. *Acute myocardial infarction (AMI)* was defined according to the third universal definition of myocardial infarction:
   - When there is clinical evidence of myocardial necrosis in a clinical setting consistent with myocardial ischaemia, or
     Detection of a rise and/or fall of cardiac biomarker values and with at least one of the following: i) symptoms of ischaemia, ii) new or presumed new ST-segment-T wave changes or new left bundle branch block, iii) development of pathological Q waves in the ECG, iv) imaging evidence of new loss of viable myocardium or new regional wall motion abnormality, or v) identification of an intracoronary thrombus by angiography or autopsy.
   - Sudden (abrupt) unexplained cardiac death, involving cardiac arrest or no evidence of a non-coronary cause of death
   - Prevalent AMI is considered to last from the onset of the event to 28 days after the event and is divided into an acute phase (0-2 days) and subacute (3-28 days).
2. *Chronic ischaemic heart disease*
   - Chronic ischaemic heart disease was estimated for survivors of myocardial infarction or those with chronic stable angina. The case definition is that of individuals with current or past history of clinically diagnosed obstructive (hemodynamically significant) coronary artery disease with evidence for myocardial ischaemia (history of myocardial infarction, physician diagnosis of chronic stable angina, evidence for inducible ischaemia on formal stress testing, or obstructive CAD by angiography), including individuals with a history of coronary revascularization
   - Chronic stable angina is symptomatic stable exertional angina pectoris or definite angina pectoris according to the Rose Angina Questionnaire (RAQ), a physician's clinical diagnosis, or taking nitrate medication for the relief of chest pain.

     The GBD study does not use estimates based on ECG evidence for prior MI, due to its limited specificity and sensitivity.

Stroke, including ischaemic stroke and haemorrhagic stroke
Stroke was defined according to WHO criteria – rapidly developing clinical signs of focal (at times generalized) disturbance of cerebral function lasting more than 24 hours or leading to death with no apparent cause other than that of vascular origin. Data on transient ischaemic attack (TIA) were not included.

1. *Acute stroke:* Stroke cases were considered acute from the data of incidence of a first ever stroke through day 28 following the event.
2. *Chronic stroke*: Stroke cases were considered chronic beginning 28 days following the occurrence of an event. Chronic stroke includes the sequelae of an acute stroke and all recurrent stroke events.
3. *Ischaemic stroke*: Incident ischaemic stroke was defined as the occurrence of first-ever ischaemic stroke, based on clinical diagnosis by a physician using diagnostic imaging. Ischaemic strokes are considered to include all vascular events leading to limited blood flow to brain tissue, with resulting infarction, including atherosclerotic and thromboembolic strokes but excluding strokes in which the underlying cause is intracranial haemorrhage.
4. *Haemorrhagic or other strokes*: This cause included all non-ischaemic strokes of a vascular cause including subarachnoid and stroke due to intracranial haemorrhage.

Rheumatic heart disease (RHD)
RHD was defined as a clinical diagnosis by a physician with or without confirmation using echocardiography. This case definition for echocardiographic confirmation of RHD follows the World Heart Federation criteria for definite echocardiographic diagnosis of rheumatic heart disease (Reményi, B. et al. Nat. Rev. Cardiol. 2012; 9, 297–309). Individuals with borderline echocardiographic evidence of RHD were not included.

Hypertensive heart disease
Hypertensive heart disease was defined as symptomatic (at least class 2 of the New York Heart Association (NYHA) Functional Classification) heart failure in which the cause of myocardial dysfunction is directly due to systemic hypertension causing left ventricular adverse remodelling. This health state may exist at several different pathologic stages during which symptomatic heart failure is present, including heart failure with preserved left ventricular ejection fraction due to left ventricular hypertrophy with diastolic dysfunction as well as heart failure with reduced left ventricular ejection fraction due to advanced adverse remodelling.

Atrial fibrillation and flutter
Atrial fibrillation was defined as a diagnosis with atrial fibrillation or atrial flutter by electrocardiography findings.

Aortic aneurysm
Death due to aortic aneurysm was defined as death due to a nondramatic thoracic and/or abdominal aneurysm, including due to the dissection or rupture of such an aneurysm.

Other cardiovascular and circulatory diseases
Cardiovascular and circulatory diseases was a residual category composed of a range of conditions, including pulmonary embolism and non-rheumatic valvular disease.

Cardiomyopathy and myocarditis
Myocarditis referred to a heterogeneous group of diseases with variable clinical and pathological features. It consists of three subcauses:

- *Acute myocarditis* was defined as the acute and time-limited symptoms of myocarditis, a condition of myocyte inflammation and dysfunction due to infectious, autoimmune, or other causes. Myocarditis was nonspecific and included a flu-like or gastrointestinal syndrome, followed by angina type chest pain, arrhythmias, syncope, or heart failure.
- *Alcoholic cardiomyopathy* refered to cardiac dysfunction that was caused by chronic alcohol intake. The nonfatal component of disease burden due to alcoholic cardiomyopathy was captured as part of the heart failure modelling process.
- *Other cardiomyopathy* was a residual category of all other types of cardiomyopathy not including alcoholic cardiomyopathy and myocarditis. The category of other cardiomyopathy included all primary myocardial diseases not due to myocarditis or alcohol exposure.

Endocarditis
The case definition for acute endocarditis was based on the clinical diagnosis of infective endocarditis.

Peripheral arterial disease
Peripheral arterial disease was defined as having an ankle-brachial index (ABI) <0.9. Intermittent claudication is defined clinically.

Heart failure and other cardiovascular disease impairment
Heart failure was diagnosed clinically following the Framingham criteria for an episode of acute congestive or right heart failure. ACC/AHA Stage C and above were used to capture both persons who were currently symptomatic and those who had been diagnosed with heart failure, but were currently asymptomatic.
Framingham criteria:

- Major criteria: Paroxysmal nocturnal dyspnea, neck vein distention, rales, radiographic cardiomegaly (increasing heart size on chest radiography), acute pulmonary edema, S3 gallop, Increased central venous pressure (>16 cm H_2O at right atrium), hepatojugular reflux; weight loss >4.5 kg in 5 days in response to treatment.
- Minor criteria: Bilateral ankle edema, nocturnal cough, dyspnea on ordinary exertion, hepatomegaly, pleural effusion, decrease in vital capacity by one third from maximum recorded, tachycardia (heart rate>120 beats/min).

B. List of ICD codes mapped to the GBD cause list

The codes used by GBD Study 2016 from the 9th and 10th revisions of the International Statistical Classification of Diseases and Related Health Problems (ICD) are listed below:

Cause	ICD10	ICD9
Cardiovascular diseases	B33.2, G45-G46.8, I01-I01.9, I02.0, I05-I09.9, I11-I11.9, I20-I25.9, I28-I28.8, I30-I31.1, I31.8-I41.9, I42.1-I42.8, I43-I43.9, I47-I48.9, I51.0-I51.4, I60-I63.9, I65-I66.9, I67.0-I67.3, I67.5-I67.6, I68.0-I68.2, I69.0-I69.3, I70.2-I70.8, I71-I73.9, I77-I83.9, I86-I89.0, I89.9, I98, K75.1	036.4, 074.2, 391-391.9, 392.0, 393-398.9, 402-402.9, 410-414.9, 417-417.9, 420-423, 423.1-424.9, 425.0-425.3, 425.5, 425.7-425.8, 427-427.3, 427.6-427.8, 429.0, 430-435.9, 437.0-437.2, 437.5-437.8, 440.2, 440.4, 441-443.9, 447-454.9, 456, 456.3-457, 457.1, 457.8-457.9, 459, 459.1-459.3
Ischaemic heart disease	I20-I25.9	410-414.9
Stroke	G45-G46.8, I60-I61.9, I62.0-I62.03, I63-I63.9, I65-I66.9, I67.0-I67.3, I67.5-I67.6, I68.1-I68.2, I69.0-I69.398	430-435.9, 437.0-437.2, 437.5-437.8
Rheumatic heart disease	I01-I01.9, I02.0, I05-I09.9	391-391.9, 392.0, 393-398.99
Hypertensive heart disease	I11-I11.9	402-402.91
Atrial fibrillation and flutter	I48-I48.92	427.3-427.32
Aortic aneurysm	I71-I71.9	441-441.9
Other cardiovascular and circulatory diseases	A39.5-A39.50, A39.53, I28-I28.8, I30-I31.1, I31.8-I32.8, I34-I37.9, I47-I47.9, I51.0-I51.3, I68.0, I72-I72.9, I77-I83.93, I86-I89.9, I91.9, I98	036.41, 074.2, 074.21, 417-417.9, 420-420.99, 423, 423.1-424.8, 424.99, 427-427.2, 427.6-427.89, 442-443, 447-454.9, 456, 456.3-457.9, 459, 459.1-459.39
Cardiomyopathy and myocarditis	A39.52, B33.2-B33.24, D86.85, I40-I43.9, I51.4-I51.5	036.43, 036.6, 074.23, 422-422.99, 425-425.9, 429.0-429.1
Endocarditis	A39.51, I33-I33.9, I38-I39.9	036.42, 074.22, 421-421.9, 424.9-424.91
Peripheral arterial disease	I70.2-I70.92, I73-I73.9	440.2-440.29, 440.4-440.9, 443-443.9

C. GBD data and analysis framework

The overview of data inputs and analysis framework for GBD is shown in the following flowchart:

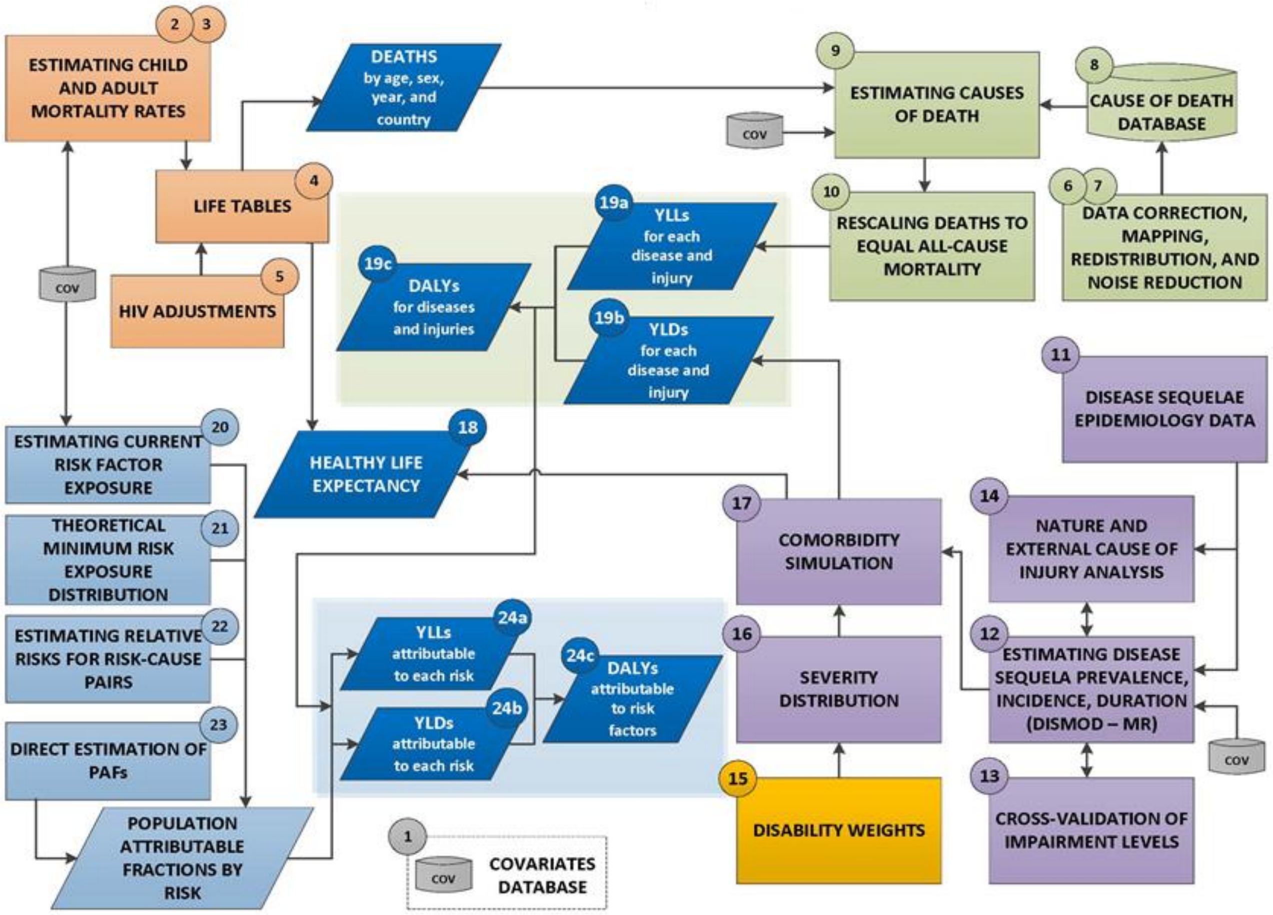

YLLs is years of life lost. YLDs is years lived with disability. DALYs is disability-adjusted life-years. PAFs is population attributable fractions. Rectangular boxes represent analytical steps, cylinders represent databases, and parallelograms represent intermediate and final results.

The flowchart above illustrates the flow of the key components of the GBD estimation process, including:

1. Incorporation of appropriate covariates (step 1)
2. All-cause mortality estimation (steps 2-5): the data come from sources such as censuses, surveys and vital registrations. The all-cause mortality estimation process (steps 2-4) can be divided into four distinct but interconnected areas: child mortality and adult mortality between ages 15 and 60, estimation of a complete set of age-specific death rates, estimation of HIV mortality and final estimates of age-specific mortality including HIV and fatal discontinuities (also known as mortality shocks) (step 5).
3. Causes of death estimation (steps 6-9): cause of death data are derived from vital registrations, verbal autopsy studies, mortality surveillance and, for selected causes, police records, crime reports and data collection systems for deaths due to conflict and natural disasters (step 7). Extensive data corrections and redistributions of ill-defined causes are made to correct for measurement bias between data sources. Cause of death ensemble modelling (CODEm), an ensemble model, is a systematized approach to analysing cause of death data for all but a few causes (step 9). CODEm explores a wide range of modelling approaches and varying predictive covariates to find an ensemble of best-performing models based on statistical tests. To do so, 30% of the data are withheld from each model and the model fit is evaluated by how well it covers the data that were left out. By repeating this process many times over the best performing models are selected. As all results in GBD are estimated 1,000 times over to propagate all sources of uncertainty, we end up with an ensemble of up to 100 or more different types of models and covariates that are selected among the 1,000 runs.
4. Rescaling deaths to equal all-cause mortality (step 10): as all these estimates are made separately for each disease and injury, the sum of these could exceed or fall below the all-cause mortality estimated from the demographic analyses of steps 2 to 5. Therefore, we rescale all deaths by age, sex, geography, year and cause to match the all-cause death estimates (this process is called CoDCorrect).
5. Estimation of disease sequelae prevalence, incidence, and duration (steps 11-12): population surveys, cohort studies, administrative records of hospitalisations and other health service encounters, disease registries, notifications, surveillance systems are the main data sources for non-fatal estimation (step 11). Extensive corrections of data to deal with measurement bias arising from study design or case definitions are applied. DisMod-MR 2.1 is the main analytical tool for non-fatal estimation (step 12). It is a Bayesian meta-regression software program that uses a lognormal model. The meta-regression component allows corrections for known sources of measurement error. Its core function is to make estimates of prevalence and incidence of disease that are consistent with data on mortality risk and remission (defined in GBD as the 'cure rate'). For a select number of causes that do not fit well in the three state model (alive without disease, prevalent case of disease and death) of DisMod-MR 2.1 we use alternative modelling strategies.
6. Cross-validation of impairment levels (step 13): for a number of impairments in GBD terminology, such as anaemia, heart failure, hearing and vision loss, we first estimate the total levels of prevalence and incidence and then ensure that all sequelae of diseases that lead to this impairment add up to the total.
7. Analysis of the nature and external cause of injury is done separately (step 14).
8. Assignment of severity distributions for the main disabling conditions (step 15): in GBD terminology sequelae are the disabling consequences for which we make estimates. All sequelae are defined to be mutually exclusive and collectively exhaustive. Many diseases have sequelae with a gradation by severity such as mild, moderate and severe dementia. Often the epidemiological data on severity distribution is sparse. Therefore, we first model the epidemiology of all cases of disease and then apply a severity distribution from the sparser data.
9. Assignment of disability weights for health states (step 16): each sequela is matched with a health state or combination of health states for which we have a disability weight which quantifies the relative severity. Disability weights were derived from population and internet surveys of over 60,000 respondents answering pair-wise comparison question of random combinations of health states. Each pair of health states was described with brief lay descriptions highlighting the main symptoms and impairments. Respondents were asked to nominate the 'healthier' of each presented pair. Analytical methods exist to formalise the intuition that if the majority of respondents nominate one health state in a pair as the healthier these lie farther apart on a severity scale than pairs assigned similar proportions as the healthier. In order to anchor estimates on a 0-1 scale of severity, a subset of respondents was asked additional population health equivalence questions on a selection of health states. These questions ask for a choice of the greater amount of health produce by two health programs; one that prevented sudden death in 1,000 persons and another that prevented the onset of a GBD health state for the rest of 2,000, 5,000 or 10,000 persons' lives.
10. Simulation of comorbidity (step 17): the last step of non-fatal estimation is a microsimulation ('COMO") to deal with comorbidity. For every age, sex, geography and year, 40,000 hypothetical persons are generated who have none, one or more of the GBD sequelae. In those with multiple sequelae their combined level of disability is estimated multiplicatively. That means we assume the disability from having two health states is

less than the sum of the corresponding disability weights. This avoids assigning disability greater than one to any individual which would indicate that person is worse off than being dead.

11. Estimation of healthy life expectancy (step 18): health life expectancy is estimated from the life tables generated in step 4 and the all-cause YLD rates from step 19b.
12. Computation of YLLs, YLDs, and DALYs from diseases and injuries with uncertainty (steps 19a-19c): YLLs (step 19a) are estimated as the product of counts of death by ages, sex, geography, year and cause and a normative life expectancy at the age of the death. The GBD standard life expectancy used as this norm is a compilation of the lowest observed mortality rates by age in all mortality data collections of populations greater than 5 million. The standard life table reflects a life expectancy at birth of 86.59 years. YLDs are the output from COMO (step 19b). DALYs are the simple addition of YLLs and YLDs (step 19c).
13. Risk factor estimation (steps 20-24): GBD 2016 also makes estimates for individual and combined risk factors. This involves estimation of risk factor exposure (step 20); the formulation of a minimum level of exposure to each risk that is associated with the least amount of health loss (step 21); derivation of relative risks of disease outcomes for each pair of a risk factor and a disease or injury for which there is judged to be sufficient evidence of a causal relationship (step 22); and the estimation of population attributable fractions of disease caused by each risk factor. For a few risk-outcome pairs it is hard to define exposure and a corresponding risk while directly observed proportions of disease are available, such as for the proportion of HIV/AIDS due to unsafe sex or injecting drug use (step 23). For combinations of risks we assess how much of the risk is mediated through other risks (step 24). For instance, all of the effect of high salt intake is mediated through elevated blood pressure and part of the risk of increased body mass index is through elevated blood pressure, cholesterol or fasting plasma glucose.
14. Computation of YLLs, YLDs, and DALYs attributable to risk factors (steps 25a-25c): YLLs, YLDs and DALYs attributable to each risk factor are generated by multiplying population attributable fractions with disease estimates (steps 25a-c).

D. Cardiovascular diseases morbidity estimation

Cardiovascular disease morbidity was modelled using the DisMod-MR 2.1 platform. Morbidity estimation and modelling methods for the major cardiovascular diseases (ischaemic heart disease, stroke and rheumatic heart disease) presented in this paper are described below. The modelling methods for the remaining cardiovascular diseases are available in the GBD 2016 non-fatal capstone paper (Lancet 2017; 390: 1211–59).

D.1. Ischaemic heart disease

The steps in the estimation of non-fatal ischaemic heart disease burden or morbidity are shown in the following flowchart:

Ischemic Heart Disease

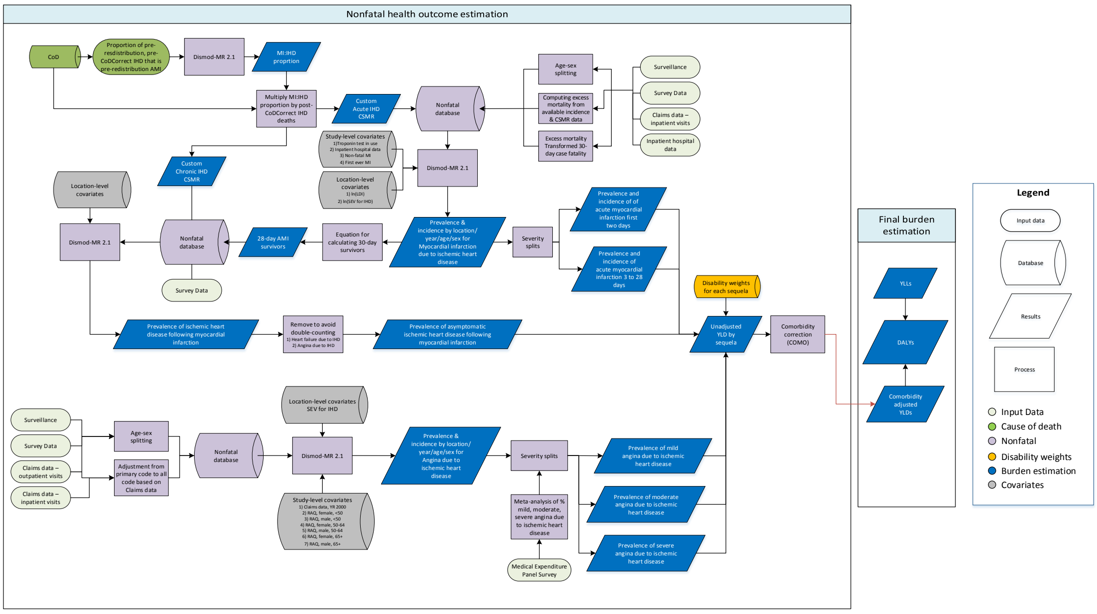

Data

Myocardial infarction

We included data from sources in the literature for myocardial infarction (MI), inpatient hospital records, and inpatient claims. We excluded data with broad age ranges where it was impossible to obtain more granular data, as these data caused the known age pattern for increased risk of myocardial infarction to be masked in the estimates generated from DisMod.

We corrected inpatient hospital data outside of DisMod to account for the fact that these data sources do not capture the out-of-hospital cardiac arrest deaths which are part of the universal definition of MI.

Using as a model the adjustment factors developed to translate tobacco consumption prevalence to tobacco consumption frequency, we matched administrative hospital data to population-based literature data based on age group, sex, and super region. For the adjustment factor, we developed the following generalized additive model on matched data:

$$\ln(\mu_{ref,i}) = \beta_0 + \beta_1 \ln(\mu_{b,i}) + s(age_i) + \varepsilon_i$$

where i represents a given matched observation, s represents a penalized spline where the smoothing parameter is chosen through cross validation, and μ_{ref} and μ_b denote the mean of the data point from literature and the mean of the inpatient hospital data point, respectively. Predictions from the model were then taken as the adjusted data points. The standard error of each corrected data point was adjusted to account for the uncertainty due to the correction.

We included a study-level covariate to correct for the change in diagnostic criteria to include troponin measurements within DisMod. This adjustment was applied to data collected before 2000. We also included a study-level covariate to adjust data points within DisMod that captured only first-ever MI, using studies where all events were included as the reference. We also adjusted estimates within DisMod from studies that only included non-fatal cases using study-level covariates with sources that included fatal and non-fatal cases as reference.

Angina

We included survey data which included the RAQ items. Prevalence of angina was calculated using the standard algorithm to determine whether the RAQ was positive or negative.

We excluded data with broad age ranges where it was impossible to obtain more granular data, as these data caused the known age pattern for increased risk of angina to be masked in the estimates generated from DisMod.

We included sex- and age-group-specific covariates to adjust prevalence data points obtained from the RAQ using the claims data as the reference since the RAQ has been shown to be neither sensitive nor specific.

We also included claims data, but did not include inpatient hospital data. Stable angina (unstable angina is modelled as part of MI) is expected to be rare in inpatient, but common in outpatient data as it is a condition usually managed on an outpatient basis, except for specific surgical interventions. This discrepancy leads to implausible correction factors based on inpatient/outpatient information from claims data (~150X); thus adjusted data cannot be used. Including uncorrected data in the model is likely to lead to incorrect estimates as hospitalization and procedure rates are likely to vary between geographies based on access to and patterns of care. All outpatient data were excluded as it was implausibly low for all locations when compared with literature and claims data.

Modelling strategy

Myocardial infarction

We first calculated custom cause-specific mortality estimates using cause of death data prior to garbage code redistribution, generating age-sex-country-specific proportions of IHD deaths that were due to MI (acute IHD) vs those due to other causes of IHD (chronic IHD). Estimates of this proportion for all locations were then generated using a DisMod proportion-only model. Due to a high degree of variability in pre-redistribution coding practices by location, we used the global age-, sex-, and year-specific proportions of acute deaths in subsequent calculations. The global proportions were multiplied by post-CodCorrect (final GBD estimates) IHD deaths to generate cause-specific mortality rates (CSMR) estimates for MI, even though GBD reports only deaths for all IHD taken together. These data, along with incidence and excess mortality data, informed a DisMod model to estimate the prevalence and incidence of myocardial infarction due to ischaemic heart disease.

These estimates were split into prevalence and incidence estimates for days 1-2 and days 3-28 post-event. Disability weights were assigned to each of these two groupings.

We set a value prior of one month for remission (11/13) from the MI health state. We also set a value prior for the maximum excess mortality rate of 10 for all ages. We included lag-distributed income (LDI) as a fixed-effect country-level covariate on excess mortality, forcing an inverse relationship.

Covariate	Parameter	Beta	Exponentiated beta
Diagnostic blood sample (troponin)	Incidence	-0.45 (-0.47 – -0.44)	0.64 (0.62 - 0.64)
Healthcare access and quality index	Excess mortality rate	-0.1 (-0.1 – -0.1)	0.9 (0.9 – 0.9)
First ever MI	Incidence	-0.66 (-0.67 – -0.65)	0.52 (0.51 – 0.52)
Non-fatal MI	Incidence	-0.40 (-0.41 – -0.40)	0.67 (0.66 – 0.67)
Log-transformed age-standardised SEV scalar: IHD	Incidence	1.24 (1.21 – 1.25)	3.44 (3.34 – 3.49)

Acute myocardial infarction was split into two severity levels by length of time since the event – days 1 and 2 versus days 3 through 28. Disability weights were established for these two severities using the standardised GBD approach.

Severity level	Lay description	Disability weight (95% CI)
Acute myocardial infarction, days 1-2	Has severe chest pain that becomes worse with any physical activity. The person feels nauseated, short of breath, and very anxious.	0.432 (0.288–0.579)
Acute myocardial infarction, days 3-28	Gets short of breath after heavy physical activity, and tires easily, but has no problems when at rest. The person has to take medication every day and has some anxiety.	0.074 (0.049–0.105)

Asymptomatic ischaemic heart disease

Excess mortality estimates from the myocardial infarction model were used to generate data of the incidence of surviving 28 days post-event.

We used these data, along with the estimates of CSMR due to chronic IHD and excess mortality data in a DisMod model to estimate the prevalence of persons with IHD following myocardial infarction. This estimate included subjects with angina and heart failure; a proportion of this prevalence was removed in order to avoid double-counting based on evidence from the literature. The result of this step generates estimates of asymptomatic ischaemic heart disease following myocardial infarction.

We set a value prior of 0 for remission for all ages. No study- or country-level covariates were included for the model.

Angina

We used prevalence data from the literature and claims databases, along with data on mortality risk to estimate the prevalence and incidence of angina for all locations. The proportion of mild, moderate, and severe angina was determined by the standard approach for severity splitting.

We included: prior of 0 for remission for all ages, prior of 1 for excess mortality for all ages. We included age- and sex-specific study-level covariates to adjust data points based on RAQ, using data points from the claims database as the reference. We also included the log-transformed, age-standardised SEV scalar for IHD as a fixed effect country-level covariate.

Covariate	Parameter	Beta	Exponentiated beta
RAQ, female, less than 50	Prevalence	2.33 (2.30 – 2.40)	10.28 (9.98 – 11.06)
RAQ, male, less than 50	Prevalence	0.94 (0.92 – 0.95)	2.57 (2.51 – 2.59)
RAQ, female, 50 to 64	Prevalence	1.44 (1.35 – 1.50)	4.22 (3.87 – 4.47)
RAQ, male, 50 to 64	Prevalence	0.83 (0.80 – 0.90)	2.30 (2.23 – 2.46)
RAQ, female, 65 plus	Prevalence	0.29 (0.27 – 0.30)	1.34 (1.31 – 1.35)
RAQ, male, 65 plus	Prevalence	0.19 (0.11 – 0.29)	1.21 (1.11 – 1.33)
Log-transformed age-standardised SEV scalar: IHD	Prevalence	1.25 (1.23 – 1.25)	3.47 (3.43 – 3.49)
LDI (I$ per capita)	Excess mortality rate	-0.55 (-1 – -0.1)	0.58 (0.37 – 0.90)

Angina was split into mild, moderate, and severe groups using information from Medical Expenditure Panel Survey. Disability weights were established for these severities using the standardised GBD approach.

Severity level	Lay description	Disability weight (95% CI)
Mild angina	Has chest pain that occurs with strenuous physical activity, such as running or lifting heavy objects. After a brief rest, the pain goes away.	0.033 (0.02–0.052)
Moderate angina	Has chest pain that occurs with moderate physical activity, such as walking uphill or more than half a kilometer (around a quarter-mile) on level ground. After a brief rest, the pain goes away.	0.08 (0.052–0.113)
Severe angina	Has chest pain that occurs with minimal physical activity, such as walking only a short distance. After a brief rest, the pain goes away. The person avoids most physical activities because of the pain.	0.167 (0.11–0.24)

D.2. Stroke

The steps in the estimation of non-fatal stroke burden or morbidity are shown in the following flowchart:

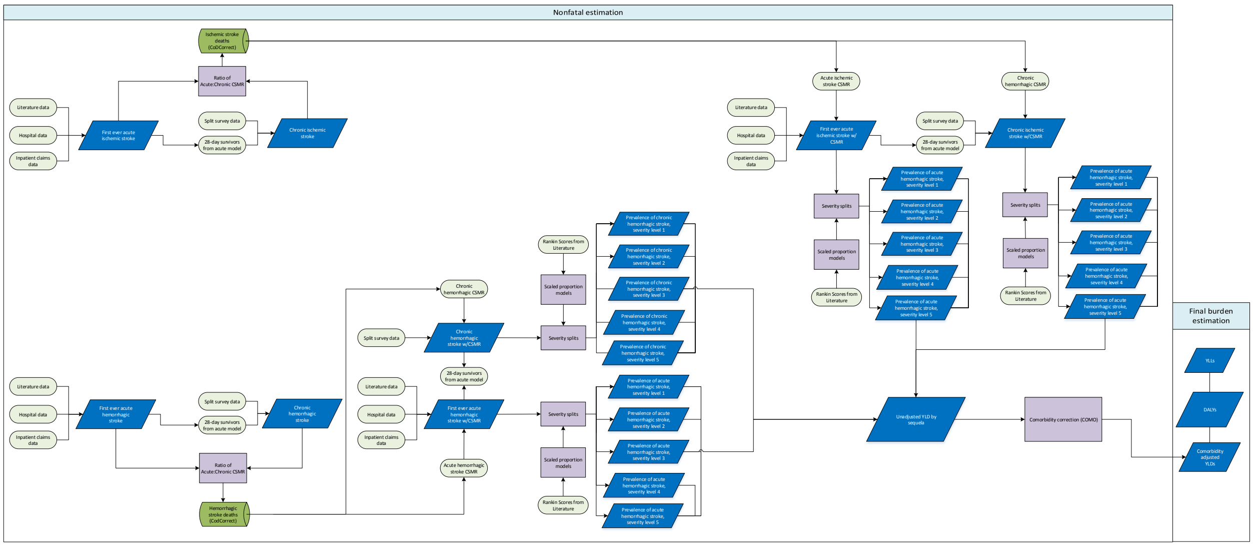

Data

We included inpatient hospital data, adjusted for readmission and primary to any diagnosis using correction factors estimated from claims data. In addition, we included unpublished stroke registry data for acute ischaemic and acute haemorrhagic strokes. We also included survey data for chronic stroke. These surveys were identified based on expert opinion and review of major survey series focused on world health that included questions regarding self-reported history of stroke.

As with many models in GBD, the diversity of data sources available means that we needed to adjust available data to our preferred or reference case definition. For the first ever acute stroke models we used DisMod to estimate the statistical association between measurements taken using different case definitions and then used these estimates to adjust the non-referent datapoints. We included study-level covariates to adjust data points for first and recurrent strokes combined, using data for first strokes only as reference. We also included study-level covariates to adjust ischaemic and haemorrhagic strokes combined (all stroke), using as reference studies with subtype-specific information.

Modelling strategy

We used DisMod MR 2.1, a Bayesian meta-regression tool, to model the six severity levels, with an independent proportion model for each. Reports which grouped mRS scores differently than our mapping (e.g. 0-2) were adjusted in DisMod by estimating the association between these alternate groupings and our preferred mappings. These statistical associations were used to adjust data points to the referent category as necessary. The six models were scaled such that the sum of the proportions for all levels equaled 1.

Data points were adjusted from nonstandard to standard case definitions using estimates from statistical models generated by DisMod for the acute models. The GBD summary exposure value, which is the relative risk-weighted prevalence of exposure, for ischaemic or haemorrhagic stroke as appropriate and a covariate for country income were used as country-level covariates for all models.

Two versions of each stroke model were run, referred to as step 1 and step 2 models. First, we ran the step 1 DisMod-MR models for acute and chronic subtype-specific stroke using only incidence, prevalence, and case fatality data as inputs. We then used the ratio of acute to chronic cause-specific mortality estimated by these models to divide GBD stroke deaths into acute and chronic stroke deaths, using the global average for the proportion of acute to chronic stroke mortality. The acute and chronic models were then run (step 2) using the same incidence, prevalence, and case fatality data as well as the custom CSMR as input data.

Step 1

We generated estimates for first-ever acute ischaemic and first-ever acute haemorrhagic stroke using DisMod-MR 2.1 with data collected on stroke incidence and excess mortality. We set value priors of 11 to 13 on remission for all ages to establish a one-month duration for these acute sequelae. We then calculated the rate of surviving until 28 days after an acute event for both ischaemic and haemorrhagic stroke using the modelled estimates of excess mortality and incidence. These survivor data were then used in the chronic ischaemic and chronic haemorrhagic stroke models as incidence inputs. We then ran the chronic stroke models, using the survivor incidence data and excess mortality data. Non-subtype-specific prevalence data were split into ischaemic and haemorrhagic components using the ratio of 28-day survivors from the first stage acute models. We set a value prior of 0 on remission for all ages. Implausible or extreme outliers in input data were dropped from these estimation results. From these four models, we generated the proportions of deaths due to acute ischaemic, chronic ischaemic, acute haemorrhagic, and chronic haemorrhagic stroke, and split the post-CoDCorrect stroke deaths generated from the GBD mortality estimates into these four parts, by multiplying the location-, sex-, age- and year-specific CSMR results by the global proportions estimated from the DisMod models. Thus, the mortality rates due to acute ischaemic, chronic ischaemic, acute haemorrhagic, and chronic haemorrhagic stroke are driven by all available data on incidence, prevalence, and excess mortality data for stroke. These CSMR estimates were then uploaded into the non-fatal database and used as inputs for models in Step 2.

Step 2

We re-ran the first-ever acute ischaemic and first-ever acute haemorrhagic models with CSMR as derived from CoDCorrect and epidemiologic data as described above. Twenty eight day survivorship was

recalculated from these models and uploaded into the chronic ischaemic and chronic haemorrhagic stroke with CSMR models. These chronic models also use CSMR as derived from CoDCorrect and epidemiologic data as described above. Implausible or extreme outliers were dropped from these estimation results.

Models were evaluated based on expert opinion, comparison with previous iterations, and model fit.

Coefficients for the covariates used in the modelling for fixed effects are listed below.

Step 1:

**Cause**	**Variable name**	**Measure**	**beta**	**Exponentiated beta**
Chronic ischaemic stroke	Log-transformed SEV scalar: Isch Stroke	Prevalence	0.83 (0.75 — 1.03)	2.29 (2.12 — 2.80)
Chronic ischaemic stroke	LDI (I$ per capita)	Excess mortality rate	-0.16 (-0.29 — -0.1)	0.85 (0.75 — 0.90)
Chronic haemorrhagic stroke	Log-transformed SEV scalar: Hem Stroke	Prevalence	0.79 (0.75 — 0.92)	2.21 (2.12 — 2.50)
Chronic haemorrhagic stroke	LDI (I$ per capita)	Excess mortality rate	-0.12 (-0.16 — -0.1)	0.89 (0.85 — 0.90)
First ever acute haemorrhagic stroke	Hospital data	Incidence	0.54 (0.54 – 0.54)	1.71 (1.71 – 1.72)
First ever acute haemorrhagic stroke	Any stroke	Incidence	1.27 (1.27 – 1.28)	3.57 (3.56 – 3.59)
First ever acute haemorrhagic stroke	First-ever acute stroke, ischaemic or haemorrhagic	Incidence	0.52 (0.52 – 0.53)	1.69 (1.68 – 1.71)
First ever acute haemorrhagic stroke	Log-transformed age-standardised SEV scalar: haemorrhagic stroke	Incidence	0.77 (0.75 – 0.82)	2.17 (2.12 – 2.27)
First ever acute haemorrhagic stroke	Any stroke	Excess mortality rate	-0.48 (-0.66 – -0.32)	0.62 (0.52 – 0.73)
First ever acute haemorrhagic stroke	First-ever acute stroke, ischaemic or haemorrhagic	Excess mortality rate	-0.081 (-0.3 – 0.16)	0.62 (0.52 – 0.73)
First ever acute ischaemic stroke	Hospital data	Incidence	0.38 (0.37 – 0.38)	1.46 (1.45 – 1.46)
First ever acute ischaemic stroke	Any stroke	Incidence	0.31 (0.29 – 0.33)	1.37 (1.34 – 1.39)
First ever acute ischaemic stroke	First-ever acute stroke, ischaemic or haemorrhagic	Incidence	0.37 (0.36 – 0.38)	1.44 (1.43 – 1.46)
First ever acute ischaemic stroke	Log-transformed age-standardised SEV scalar: ischaemic stroke	Incidence	1.16 (1.09 – 1.22)	3.21 (2.99 – 3.39)

Step 2

**Cause**	**Variable name**	**Measure**	**beta**	**Exponentiated beta**
Chronic ischaemic stroke with CSMR	Log-transformed SEV scalar: Ischaemic stroke	Prevalence	0.89 (0.75 – 1.19)	2.44 (2.13 – 3.27)
Chronic ischaemic stroke with CSMR	LDI (I$ per capita)	Excess mortality rate	-0.49 (-0.5 – -0.46)	0.61 (0.61 – 0.63)
Chronic haemorrhagic stroke with CSMR	Log-transformed SEV scalar: Haemorrhagic stroke	Prevalence	0.88 (0.75 – 1.15)	2.40 (2.13 – 3.17)
Chronic haemorrhagic stroke with CSMR	LDI (I$ per capita)	Excess mortality rate	-0.48 (-0.5 – -0.44)	0.62 (0.61 – 0.64)
First-ever acute haemorrhagic stroke with CSMR	Any stroke	Incidence	1.27 (1.27 – 1.29)	3.58 (3.56 – 3.62)

First-ever acute haemorrhagic stroke with CSMR	First-ever acute stroke, ischaemic or haemorrhagic	Incidence	0.52 (0.52 – 0.54)	1.69 (1.68 – 1.71)
First-ever acute haemorrhagic stroke with CSMR	Log-transformed SEV scalar: Hem stroke	Incidence	1.11 (1.01 – 1.20)	3.03 (2.74 – 3.33)
First-ever acute haemorrhagic stroke with CSMR	Any stroke	Excess mortality rate	-0.37 (-0.49 – -0.27)	0.69 (0.62 – 0.77)
First-ever acute haemorrhagic stroke with CSMR	First-ever acute stroke, ischaemic or haemorrhagic	Excess mortality rate	0.023 (-0.2 – 0.23)	1.02 (0.82 – 1.25)
First-ever acute ischaemic stroke with CSMR	Any stroke	Incidence	0.32 (0.30 – 0.33)	1.38 (1.35 – 1.39)
First-ever acute ischaemic stroke with CSMR	First-ever acute stroke, ischaemic or haemorrhagic	Incidence	0.37 (0.36 – 0.38)	1.44 (1.43 – 1.46)
First-ever acute ischaemic stroke with CSMR	Log-transformed age-standardised SEV scalar: Ischaemic stroke	Incidence	1.11 (1.05 – 1.18)	3.04 (2.86 – 3.26)
First-ever acute ischaemic stroke with CSMR	Any stroke	Excess mortality rate	-0.34 (-0.45 – -0.24)	0.71 (0.64 – 0.79)
First-ever acute ischaemic stroke with CSMR	First-ever acute stroke, ischaemic or haemorrhagic	Excess mortality rate	-0.69 (-0.82 – -0.56)	0.50 (0.44 – 0.57)

The table below illustrates the severity level, lay description, and disability weights for GBD. We undertook a review to identify epidemiologic literature which reported the degree of disability at 28 days (for acute stroke) or one year (for chronic stroke) using the modified Rankin scale (mRS) and the Mini-mental State Examination (MMSE) or the Montreal Cognitive Assessment (MoCA). The mRS assesses functional capabilities, while the MMSE and MoCA tests provide evaluations of cognitive functioning. We then mapped these measures to the existing GBD categories as indicated below.

Acute stroke severity splits

**Severity level**	**Lay description**	**Modified Rankin Score**	**Cognitive Status**	**Disability weight (95% CI)**
Stroke, mild	Has some difficulty in moving around and some weakness in one hand, but is able to walk without help.	1	N/A	0.019 (0.01–0.032)
Stroke, moderate	Has some difficulty in moving around, and in using the hands for lifting and holding things, dressing, and grooming.	2, 3	MoCA>=24 or MMSE>=26	0.07 (0.046–0.099)
Stroke, moderate plus cognition problems	Has some difficulty in moving around, in using the hands for lifting and holding things, dressing and grooming, and in speaking. The person is often forgetful and confused.	2, 3	MoCA<24 or MMSE<26	0.316 (0.206–0.437)
Stroke, severe	Is confined to bed or a wheelchair, has difficulty speaking, and depends on others for feeding, toileting, and dressing.	4, 5	MoCA>=24 or MMSE>=26	0.552 (0.377–0.707)
Stroke, severe plus cognition problems	Is confined to bed or a wheelchair, depends on others for feeding, toileting, and dressing, and has difficulty speaking, thinking clearly, and remembering things.		MoCA<24 or MMSE<26	0.588 (0.411–0.744)

Chronic stroke severity splits

**Severity level**	**Lay description**	**Modified Rankin Score**	**Cognitive Status**	**Disability weight (95% CI)**

Stroke, asymptomatic		0	N/A	N/A
Stroke, long-term consequences, mild	Has some difficulty in moving around and some weakness in one hand, but is able to walk without help.	1	N/A	0.019 (0.01–0.032)
Stroke, long-term Consequences, moderate	Has some difficulty in moving around, and in using the hands for lifting and holding things, dressing, and grooming.	2, 3	MoCA>=24 or MMSE>=26	0.07 (0.046–0.099)
Stroke, long-term consequences, moderate plus cognition problems	Has some difficulty in moving around, in using the hands for lifting and holding things, dressing and grooming, and in speaking. The person is often forgetful and confused.	2, 3	MoCA<24 or MMSE<26	0.316 (0.206–0.437)
Stroke, long-term consequences, severe	Is confined to bed or a wheelchair, has difficulty speaking, and depends on others for feeding, toileting, and dressing.	4, 5	MoCA>=24 or MMSE>=26	0.552 (0.377–0.707)
Stroke, long-term consequences, severe plus cognition problems	Is confined to bed or a wheelchair, depends on others for feeding, toileting, and dressing, and has difficulty speaking, thinking clearly, and remembering things.	4, 5	MoCA<24 or MMSE<26	0.588 (0.411–0.744)

D.3. Rheumatic heart disease

The steps in the estimation of non-fatal rheumatic heart disease burden or morbidity are shown in the following flowchart:

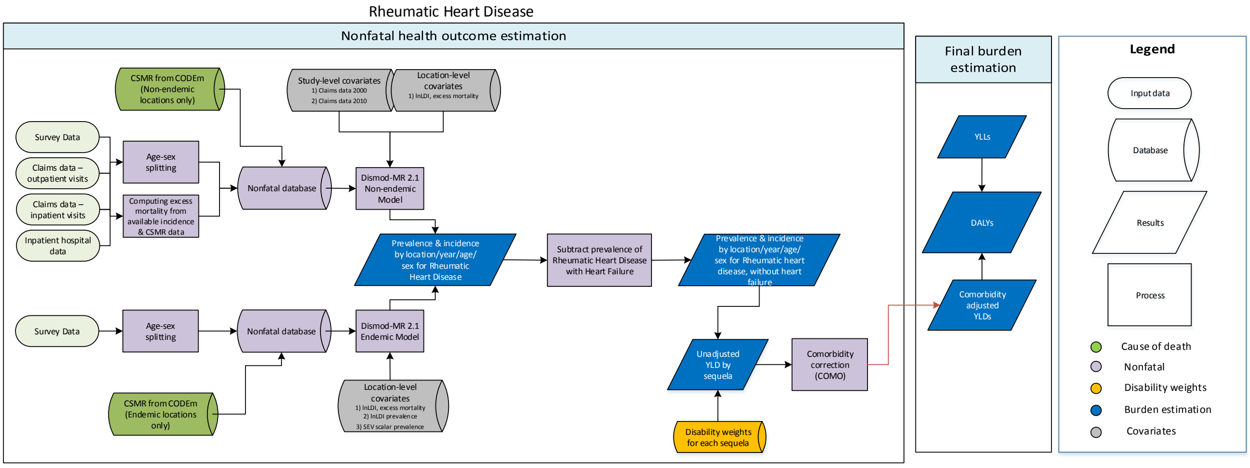

Data

We included data from sources in the literature for rheumatic heart disease. Hospital and claims records were also available for non-endemic countries.

For the endemic country model, we included study-level covariates to crosswalk studies that did not include echocardiographic confirmation of the disease diagnosis. For the non-endemic country model, we included study-level covariates for inpatient hospital data and claims data from 2000 and 2010 to adjust these data points, using as reference the data obtained from literature and claims data from 2012.

Modelling strategy

We ran two models – one for non-endemic countries and one for endemic countries. We defined endemicity based on RHD mortality estimates and the socio-demographic index (SDI) estimates for the year 2016. For mortality, we used a threshold of 0.15/100,000 deaths in children aged 5-9; for SDI, we used a cutpoint of 0.6. These thresholds were selected using prior expert opinion on locations where RHD is known to be endemic. Locations above the threshold for deaths or below the threshold for SDI were categorised as endemic; all other locations were categorised as non-endemic. These decisions were made at

the national level for countries which were previously modelled nationally, and at the subnational level for the subset of countries modelled subnationally.

Non-endemic model: We included hospital data, claims data, and limited literature data on prevalence. We also included CSMR from our mortality estimates of RHD for non-endemic locations only. A prior of no remission was set, and excess mortality was capped at 0.1 for all ages. We included study-level covariates for claims data from 2000 and 2010, cross-walking them to data from the literature and claims data in 2012. We also included the log-transformed age-standardised SEV scalar for RHD and the natural log of lagged distributed income (ln LDI, I$ per capita) as country-level covariates for prevalence and excess mortality, respectively.

Endemic model: We included prevalence data from surveys published in the literature. As with the high income model, we included CSMR from our mortality estimates of RHD for endemic locations only. A prior of no remission was set for all ages, and excess mortality was capped at 0.07, the highest observed mean excess mortality rate data point observed in this model. We also set priors of 0 on incidence for ages 0 to 1 and 50 to 100 to account for patterns of incidence in endemic countries. We used ln LDI as fixed-effect country-level covariates on prevalence and excess mortality, enforcing an inverse relationship for both. The log-transformed, age-standardised SEV scalar was also used as a fixed-effect country-level covariate on prevalence.

We combined estimates from the endemic and non-endemic models, selecting estimates for the locations identified as non-endemic from the non-endemic model and estimates for the locations identified as endemic from the endemic model. Estimates of heart failure due to RHD were then subtracted from the estimates for RHD, giving the overall prevalence of RHD without heart failure. A description of the modelling strategy for heart failure due to RHD can be found in the Heart Failure appendix. We evaluated models based on comparing estimates with input data as well as estimates from previous rounds of GBD.

The table below shows the country covariates, parameters, betas, and exponentiated betas:

**Covariate**	**Parameter**	**beta**	**Exponentiated beta**
Endemic model			
Log-transformed age-standardised SEV scalar: RHD	Prevalence	0.93 (0.76 – 1.22)	2.55 (2.13 – 3.37)
LDI (I$ per capita)	Excess mortality rate	-0.28 (-0.48 - -0.11)	0.76 (0.62 – 0.90)
Non-endemic model			
All Marketscan, year 2000	Study-level	0.19 (0.090 – 0.30)	1.21 (1.09 – 1.34)
All Marketscan, year 2010	Study-level	0.44 ((0.36 – 0.52)	1.55 (1.43 – 1.68)
Log-transformed age-standardised SEV scalar: RHD	Prevalence	0.78 (0.75 – 0.89)	2.19 (2.12 – 2.43)
LDI (I$ per capita)	Excess mortality rate	-0.54 (-0.57 - -0.5)	0.58 (0.57 – 0.61)

The disability weight used was:

**Severity level**	**Lay description**	**Disability weight (95% CI)**
Rheumatic heart disease, not including heart failure	Has a chronic disease that requires medication every day and causes some worry but minimal interference with daily activities.	0.049 (0.031–0.072)

E. Cardiovascular diseases mortality estimation

Mortality estimation and modelling methods for the major cardiovascular diseases presented in this paper (ischaemic heart disease, stroke and rheumatic heart disease) are described below. Details about the other cardiovascular diseases are available in the GBD 2016 cause of death capstone paper (Lancet 2017; 390: 1151–210).

Data

The major data inputs to determine cardiovascular diseases mortality in India were the Sample Registration System (SRS) cause of death data, Medical Certification of Cause of Death (MCCD) and some other studies.

In GBD 2016, SRS was the main data source used for the estimation of ischaemic heart disease and stroke deaths. Verbal autopsy data is most reliable for such broader cause categories. Expert opinion has suggested higher data quality for SRS compared with MCCD for these causes. SRS also has better geographic coverage. One the other hand, MCCD was the main data source used for rheumatic heart disease deaths. Such causes are generally not captured well with verbal autopsy approaches such as SRS due to their nonspecific symptoms or the need for advanced diagnostics, to make a definitive diagnosis.

SRS is operated by the Office of the Registrar General of India working under the Ministry of Home Affairs, Government of India. Cause of death data from SRS verbal autopsy included 455,460 deaths from the rural and urban populations of every state of India from 2004 to 2013 in which physicians assigned the cause of death based on the information provided in the verbal autopsy interview of a person close to each deceased person. Using the 2001 census, 7597 geographic units, 4433 (58·4%) of which were rural, were sampled for the 2004–13 SRS to represent the population of each state and union territory of India, ultimately with a sample of 6·7 million people that was equivalent to 0·7% of India's population. The SRS cause of death data for 2004–06, 2007–09, and 2010–13 were provided for each state and union territory by the Office of the Registrar General of India for use in the state-level disease burden estimation. We used 2005, 2008, and 2012 as midpoint years for these three time periods. The inclusion of SRS 2004–13 data in this analysis offers a comprehensive picture of causes of death in India. In the absence of a fully functional vital registration system, verbal autopsy can provide reasonable population level cause of death distribution (Lancet 2017; 390: 2437–2460).

The MCCD system under the Office of the Registrar General of India has data mostly for the urban parts of the states and union territories beginning in 1980. MCCD covered only 22% of the deaths in India in 2015, with the coverage less than 20% in 15 states, 20–50% in ten states and union territories, and more than 50% in some states and union territories. Deaths reported in this data source are medically certified and are considered vital registration data (Lancet 2017; 390: 2437–2460).

Modelling strategy

Mortality estimates for cardiovascular diseases were generated using the cause of death ensemble modelling (CODEm). CODEm is the framework used to model most cause-specific death rates in the GBD. It relies on four key components. First, all available data are identified and gathered to be used in the modelling process. Though the data may vary in quality, they all contain some signal of the true epidemiological process. Second, a diverse set of plausible models are developed to capture well-documented associations in the estimates. Using a wide variety of individual models to create an ensemble predictive model has been shown to outperform techniques using only a single model both in cause of death estimation and in more general prediction applications. Third, the out-of-sample predictive validity is assessed for all individual models, which are then ranked for use in the ensemble modelling stage. Finally, differently weighted combinations of individual models are evaluated to select the ensemble model with the highest out-of-sample predictive validity.

For some causes, separate models were run for different age ranges when there was reason to believe that the relation between covariates and death rates might be different in different age ranges, for example, in children compared with adults. Separate models are developed for countries with extensive, complete, and representative VR for every cause such that uncertainty can better reflect the more complete vital registration in these locations.

As many factors covary with a particular cause of death, a large range of plausible statistical models are developed for each cause. For the CODEm framework, four families of statistical models are developed using covariates. These are mixed effects linear models of the natural log of the death rate, mixed effects linear models of the logit of the cause fraction, spatiotemporal Gaussian process regression (ST-GPR) models of the log of the death rate, and ST-GPR of the logit of the cause fraction. All plausible relationships between covariates and relevant cause are identified, and all possible permutations of selected covariates are tested in linear models where the logit cause fraction or log death rate is the response variable. Because we test all permutations of covariates, multicollinearity between covariates may produce implausible signs on coefficients or unstable coefficients. All models where the sign on the coefficient is in the direction expected based on the literature and where the coefficient is statistically significant at $p < 0.05$ are retained. We run covariate selection for both cause fractions and death rates and then create both mixed effects only and ST models for each set of covariates.

The performance of all component models and ensembles is evaluated using out-of-sample predictive validity tests. Thirty percent of the data are excluded from the initial model fits, and half of that (15% of total) is used to evaluate and rank component models and then build ensembles. Data are held out from the analysis using the pattern of missingness for each cause in the cause of death database. Out-of-sample predictive validity testing is repeated until stable model results have been obtained. The out-of-sample performance tests include the root mean squared error of the log of the cause-specific death rate, the direction of the trend in the prediction compared to the data, and the validity of the 95% uncertainty interval (UI). For every model, we show the in-sample root mean squared error of the log death rates (RMSE) and the out-of-sample performance in the 15% of data not used in the model building process.

After component models are ranked on their out-of-sample predictive validity they are weighted based on their ranking and each component model contributes a portion to the final estimate. How much each submodel contributes is a function of its relative ranking as well as the value of psi chosen, which dictates that distribution of rankings.

Using the second half of the holdout data (15% of total), the differently weighted ensembles and different values of psi are tested using the same predictive validity metrics as the component models. For every model, we show the in-sample RMSE and the out-of-sample performance in the 15% of data not used in the model building process. The ensemble with the best average trend and RMSE is chosen as the final ensemble weighting scheme.

After a model weighting scheme has been chosen, each model contributes a number of draws proportional to its weight such that 1,000 draws are created. The mean of the draws is used as the final estimate for the CODEm process and 95% UI are created from the 0.025 and 0.975 quantiles of the draws. The final assessment of ensemble model performance is the validity of the UIs; ideally, the 95% UI for a model would capture 95% of the data out-of-sample. Higher coverage suggests that UIs are too large and lower than 95% suggest UIs are too narrow.

CODEm models estimate the individual cause-level mortality without taking into account the all-cause mortality. GBD uses the CodCorrect algorithm to ensure that all individual causes add up to the all-cause mortality. After generating underlying cause of death estimates and accompanying uncertainty, this algorithm combines these models into estimates that are consistent with the levels of all-cause mortality estimated for each age-sex-year-location group. Using 1000 draws from the posterior distribution of each cause and 1000 draws from the posterior distribution of the estimation of all-cause mortality, CoDCorrect rescales the sum of cause-specific estimates to equal the draws from the all cause distribution. Further details of CodCorrect algorithm can be found in the appendix to the GBD 2016 cause of death capstone paper (Lancet 2017; 390: 1151–210).

E.1. Ischaemic heart disease

The approach to cause of death estimation for ischaemic heart disease is shown in the following flowchart:

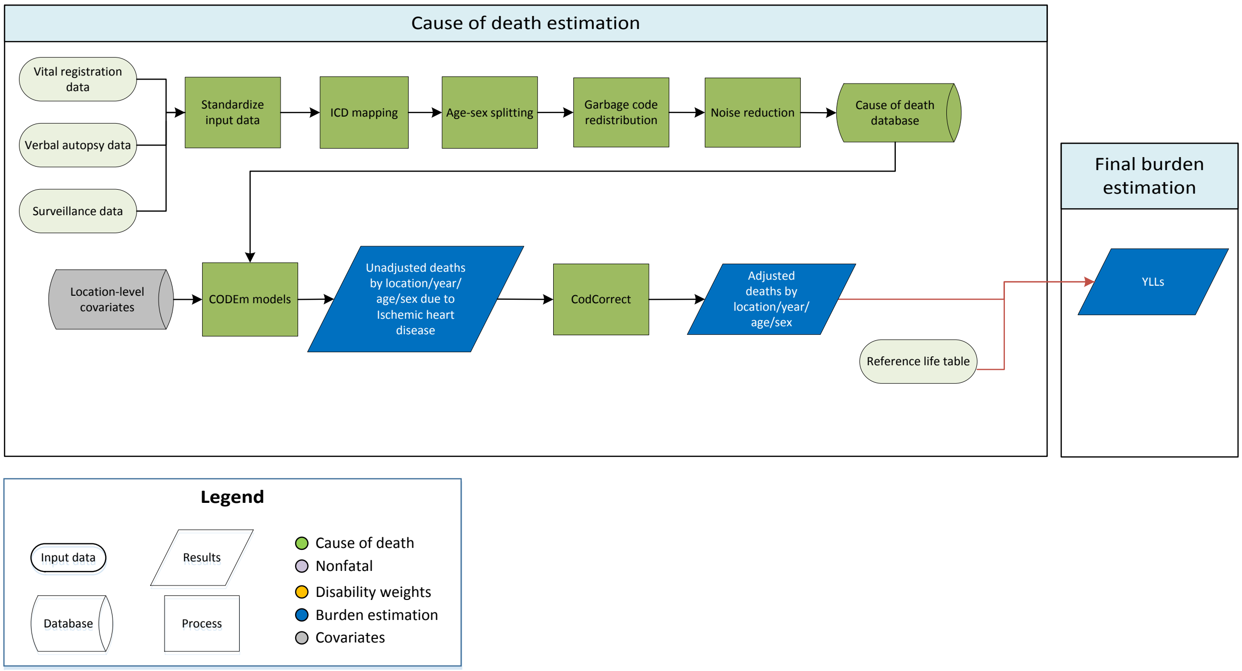

We used the standard CODEm approach to model deaths from ischaemic heart disease, which is described above. The covariates included in the model were:

Covariate	Transformation	Level	Direction
Summary exposure variable	None	1	1
Cholesterol (total, mean per capita)	None	1	2
Smoking prevalence	None	1	3
Systolic blood pressure (mmHg)	None	1	4
Trans fatty acid	None	1	5
Mean BMI	None	2	1
Elevation over 1500m (proportion)	None	2	-1
Fasting plasma glucose	None	2	1
Outdoor pollution (PM 2.5)	None	2	1
Indoor air pollution	None	2	1
Healthcare access and quality index	None	2	-1
Lag distributed income per capita (I$)	Log	3	-1
SDI	None	3	0
Omega-3 (kcal/capita, adjusted)	Log	3	-1
Fruits (kcal/capita, adjusted)	None	3	-1
Vegetables (kcal/capita, adjusted)	None	3	-1
Nuts and seeds (kcal/capita, adjusted)	None	3	-1
Whole grains (kcal/capita, adjusted)	None	3	-1
Pulses/legumes (kcal/capita, adjusted)	None	3	-1
PUFA adjusted (percent)	None	3	-1
Alcohol (litres per capita)	None	3	0

E.2. Stroke

The approach to cause of death estimation for stroke is shown in the following flowchart:

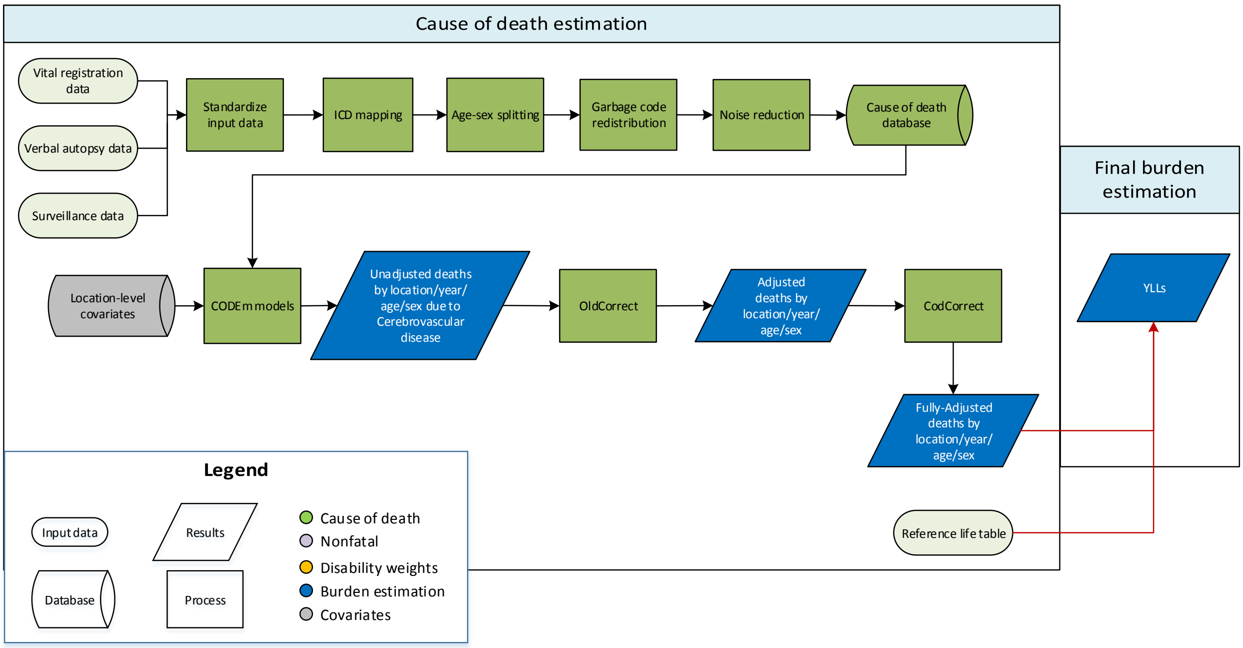

We used the standard CODEm approach to model deaths from stroke, which is described above. The covariates included in the model were:

Covariate	Transformation	Level	Direction
Summary exposure variable	None	1	1
Cholesterol (total, mean per capita)	None	1	1
Smoking prevalence	None	1	1
Systolic blood pressure (mmHg)	None	1	1
Trans fatty acid	None	1	1
Mean BMI	None	2	1
Elevation over 1500m (proportion)	None	2	-1
Fasting plasma glucose	None	2	1
Outdoor pollution (PM 2.5)	None	2	1
Indoor air pollution	None	2	1
Healthcare access and quality index	None	2	-1
Lag distributed income per capita (I$)	Log	3	-1
SDI	None	3	0
Omega-3 (kcal/capita, adjusted)	Log	3	-1
Fruits (kcal/capita, adjusted)	None	3	-1
Vegetables (kcal/capita, adjusted)	None	3	-1
Nuts and seeds (kcal/capita, adjusted)	None	3	-1
Whole grains (kcal/capita, adjusted)	None	3	-1
Pulses/legumes (kcal/capita, adjusted)	None	3	-1
PUFA adjusted (percent)	None	3	-1
Alcohol (litres per capita)	None	3	0

E.3. Rheumatic heart disease

The approach to cause of death estimation for rheumatic heart disease is shown in the following flowchart:

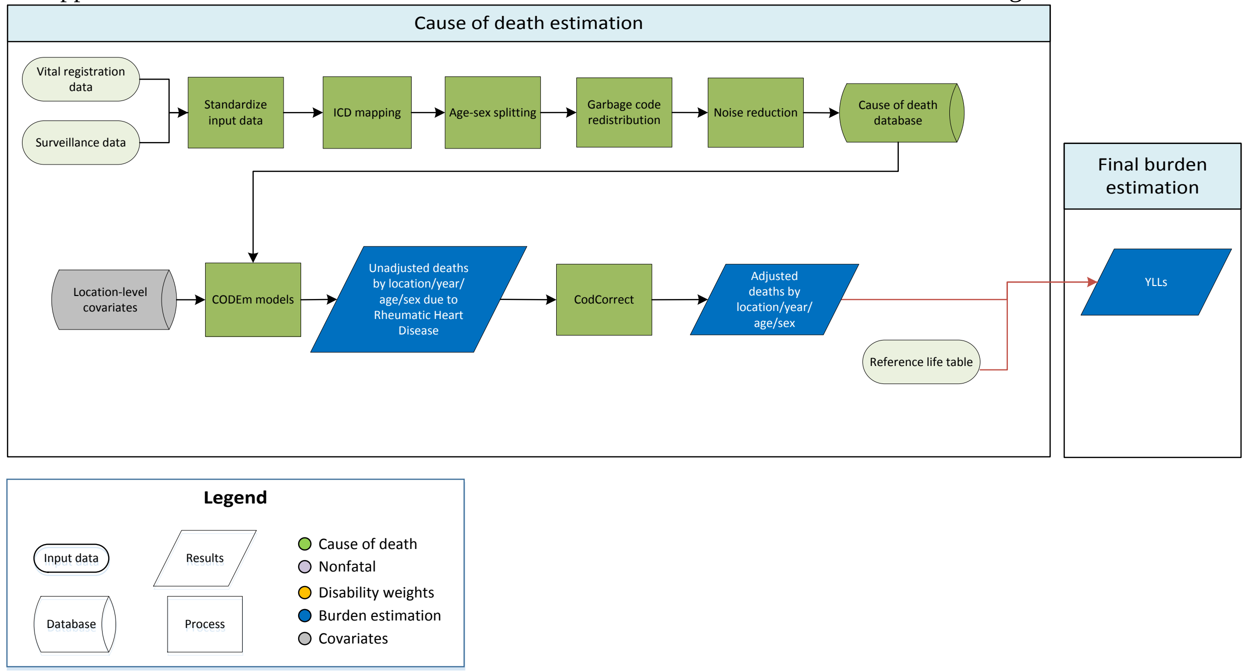

We used the standard CODEm approach to model deaths from rheumatic heart disease, which is described above. The covariates included in the model were:

Covariates	Transformation	Level	Direction
SEV	None	1	1
Improved water (proportion)	None	1	-1
Malnutrition	None	1	1
Sanitation (proportion with access)	None	1	-1
Health access and quality index	None	2	-1
LDI	Log	3	-1
SDI	None	3	-1
Education (years per capita)	None	3	-1

F. Estimation of major risk factors for cardiovascular diseases

The approach used in GBD 2016 for comparative risk assessment to estimate population attributable fractions for risk factors is shown in the following flowchart.

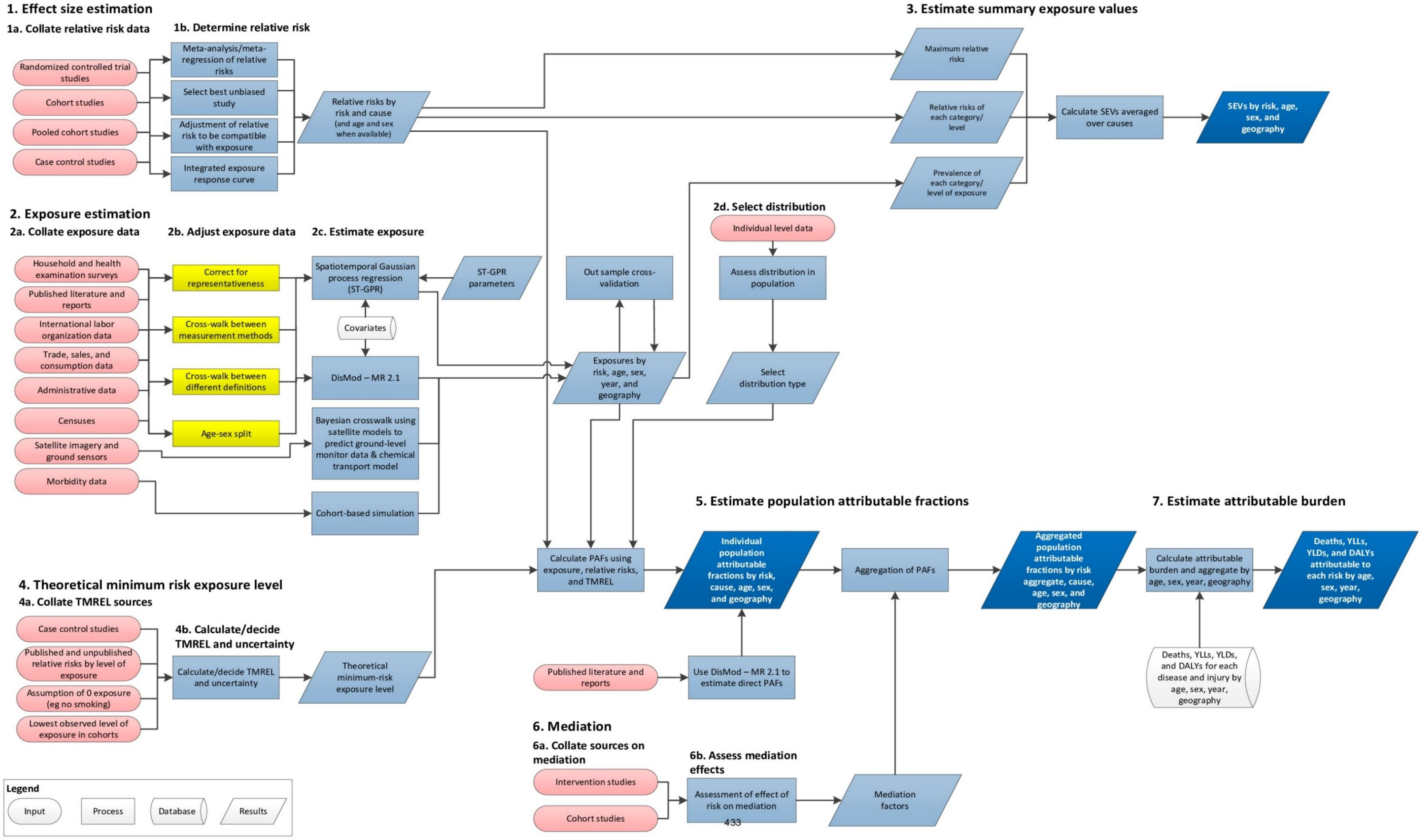

GBD is Global Burden of Disease. SEV is summary exposure value. TMREL is theoretical minimum-risk exposure level. PAF is population attributable fraction. YLL is years of life lost. YLD is years lived with disability. DALY is disability-adjusted life-year. Ovals represent data inputs, rectangular boxes represent analytical steps, cylinders represent databases, and parallelograms represent intermediate and final results.

We describe details of six major risk factors related to cardiovascular diseases, i.e. high systolic blood pressure, high total blood cholesterol, high fasting plasma glucose, smoking, dietary risks, and air pollution. Description of other risk factors is available in the GBD 2016 risk factors capstone paper (Lancet 2017; 390: 1345–422).

F.1. High systolic blood pressure

For the purpose of attributing disease burden to high systolic blood pressure (SBP), the theoretical minimum-risk exposure level (TMREL) for SBP was estimated to range from 110 to 115 mm Hg based on pooled prospective cohort studies that showed risk of mortality increases for SBP above this level. To include the uncertainty in the TMREL, we took a random draw from the uniform distribution of the interval between 110 mm and 115 mm Hg each time the population attributable burden was calculated.

The steps in the estimation of disease burden attributable to high systolic blood pressure are shown in the following flowchart:

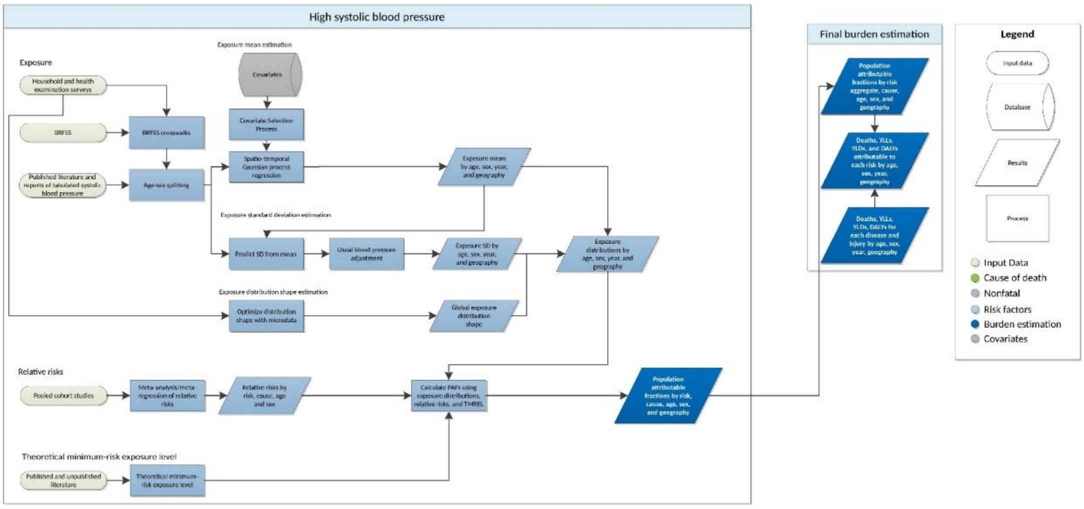

Data

We utilised data on mean systolic blood pressure from literature and from household survey microdata and reports. Studies were included if they were population-based and measured systolic blood pressure using a blood test. We assumed the data were representative of the location if the geography was not selected because it was related to the diseases associated with high blood pressure.

Data were utilised in the modelling process unless an assessment of data strongly suggested that the data were biased. A candidate source was excluded if the quality of study did not warrant a valid estimate because of selection (specific populations), different definitions, or if the study did not provide methodological details for evaluation. In a small number of cases, a data point was considered to be an outlier candidate if the level was widely inconsistent with other country data.

Where possible, individual level data on blood pressure estimates were extracted from survey microdata and these were collapsed across individuals and collapsed across demographic groupings to produce mean estimates in the standard GBD 5-year age-sex groups. If microdata were unavailable, information from survey reports or from literature were extracted along with any available measure of uncertainty including standard error, uncertainty intervals, and sample size. Standard deviations were also extracted.

Prior to modelling, data provided in age groups wider than the GBD 5-year age groups were split using a standard approach. Briefly, age-sex patterns were identified using sources of data with multiple age-sex groups and these patterns were applied to split aggregated report data.

Modelling strategy

Exposure estimates were produced from 1980 to 2016 for each national and subnational location, sex, and for each 5-year age group starting from 25+. We used a ST-GPR framework to model the mean systolic blood pressure at the location-, year-, age-, sex- level.

The first step of the ST-GPR framework requires the creation of a linear model for predicting systolic blood pressure at the location-, year-, age-, sex- level. Covariates for this model were selected in two stages. First, a list of variables with an expected causal relationship with systolic blood pressure was created based on significant association found within high-quality prospective cohort studies reported in the published scientific literature. These variables were: urinary sodium, liters per capita of alcohol, dietary availability of vegetables, dietary availability of fruits, dietary availability of omega-3 fatty acids, the prevalence of smoking, dietary availability of nuts and seeds, and the prevalence of overweight. We also used the SDI, and the health access quality index (HAQI) to represent the effect of proximal socioeconomic factors and access to health care on exposure levels. The second stage in covariate selection was to test the predictive validity of every possible combination of covariates in the linear model, given the covariates selected above. This was done separately for each sex. Predictive validity was measured with out of sample root-mean-squared error. The linear model with the lowest root-mean squared error for each sex was then used in the ST-GPR model.

For women, this linear model was:

$$\log\left(SBP_{l,a,t}\right) = \beta_0 + \beta_1 SDI_{l,t} + \beta_2 nuts + \beta_3 HAQI + \sum_{k=2}^{16} \beta_k I_{A[a]} + \alpha_s + \alpha_r + \alpha_c + \epsilon_{l,a,t}$$

For men, the linear model was:

$$\log\left(SBP_{l,a,t}\right) = \beta_0 + \beta_1 SDI_{l,t} + \beta_2 alcohol + \beta_3 HAQI + \sum_{k=2}^{16} \beta_k I_{A[a]} + \alpha_s + \alpha_r + \alpha_c + \epsilon_{l,a,t}$$

where $SDI_{c,t}$ *is* socio-demographic index, an index metric that includes a measure of education and income, $HAQI_{c,t}$ is the health access quality index, $nuts_{c,t}$ is the dietary availability of nuts and seeds, $alcohol_{c,t}$ is the liters per capita of alcohol consumed, $I_{A[a]}$ is a dummy variable for a fixed effect on a given 5-year age group, and α_s α_r α_c *are* random effects at the super-region, region, and country level, respectively. The table below contains the coefficients of the fixed effects used in the two regressions.

**Covariate**	**Coefficients from male model**	**Coefficients from female model**
Alcohol LPC	0.0003 (0.0002 to -00.0004)	NA
Nuts/seeds	-0.0024 (-0.0027 to -0.0022)	-0.0028 (-0.0031 to -0.0025)
SDI	0.088 (0.074 to 0.102)	0.121 (0.100 to 0.142)
HAQI	NA	-0.0016 (-0.0019 to -0.0014)

The estimates were then propagated through the ST-GPR framework to obtain 1000 draws for each location, year, age, and sex. The table below contains the out of sample RMSE of both the linear model and the final ST-GPR results for the male and female models.

	**Out of sample RMSEs for male model**	**Out of sample RMSEs for female model**
Linear model	6.469 mmHg	5.985 mmHg
Final ST-GPR model	3.56 mmHg	3.77 mmHg

The standard deviation of systolic blood pressure within a population was estimated for each national and subnational location, sex, and 5-year age group starting from age 25. Person-level microdata accounted for 10,375 of the total 12,570 rows of data on standard deviation. The remaining 2195 rows came from tabulated data. Tabulated data was only used to model standard deviation if it was sex and 5-year age group specific and reported a population standard deviation of systolic blood pressure. The systolic blood pressure standard deviation function was estimated using a linear regression:

$$\log(SD_{l,a,t,s}) = \beta_0 + \beta_1 SBP_{l,a,t,s} + \beta_3 (SBP_{l,a,t,s})^2 + \beta_4 sex + \sum_{k=2}^{16} \beta_k I_{A[a]} + \alpha_s + \epsilon_{l,a,t,s}$$

where $SBP_{l,a,t,s}$ is the location, age, time, and sex specific mean total cholesterol estimate from ST-GPR, $I_{A[a]}$ is a dummy variable for a fixed effect on a given 5-year age group, and α_s is a random effect at the super-region level.

To account for in-person variation in systolic blood pressure, a 'usual blood pressure' adjustment was done. Measurements of a risk factor taken at a single time point may not accurately capture an individual's true long-term exposure to that risk. Blood pressure readings are particularly susceptible to short-term variability including diurnal, seasonal, or biological variation. These sources of variation result in an over-estimation of the variation in cross-sectional risk exposure distributions.

To adjust for this overestimation, we applied a correction factor to each location-, age-, time-, and sex-specific standard deviation. These correction factors were age-specific, and represented the proportion of the variation in blood pressure within a population that would be observed if there were no within-person variation across time. Three longitudinal surveys were used to estimate these factors: the China Health and Retirement Longitudinal Survey (CHRLS), the Indonesia Family Life Survey (IFLS), and the South Africa National Income Dynamics Survey (NIDS). The sample size and number of blood pressure measurements at each measurement period for each survey is reported in the table below.

Source	Measurement period	Number of measurements	Sample size
CHRLS	2008	3	1967
	2012	3	1419
IFLS	1997	1	19418
	2000	1	16626
	2007	3	14136
NIDS	1997	2	14084
	2000	2	9612
	2007	2	9098

For each survey, the following regression was created for each age group:

$$SBP_{i,a} = \beta_0 + \beta_1 sex + \beta_3 age + +\upsilon_i$$

where $SBP_{i,a}$ is the systolic blood pressure of an individual i at age a, sex is a dummy variable for the sex of an individual, age is a continuous variable for the age of an individual, and υ_i is a random intercept for each individual. Then, a blood pressure value $\widehat{SBP}_{i,b}$ was predicted for each individual i for his/her age at baseline b. The correction factor cf for each age group within each survey was calculated as variation in these predicted blood pressures was divided by the variation in the observed blood pressures at baseline, $SBP_{i,b}$:

$$cf = \frac{var(\widehat{SBP}_{i,b})}{var(SBP_{i,b})}$$

The average of the correction factors was taken over the three surveys to get one set of age-specific correction factors, which were then multiplied by the square of the modelled standard deviations to estimate standard deviation of the 'usual blood pressure' of each age, sex, location, and year. Because of low sample sizes, the correction factors for the 75-79 age group was used for all terminal age groups. The final correction factors for each age group are reported in the table below.

Age group	Correction factor
25-29	.665
30-34	.713
35-39	.737
40-44	.733
45-49	.798
50-54	.771

55-59	.764
60-64	.753
65-69	.719
70-74	.689
75+	.678

Estimating the exposure distribution shape: the shape of the distribution of systolic blood pressure was estimated for each age group using all available person-level microdata sources, which was a subset of the input data into the modelling process.

For cardiovascular disease, epidemiological studies have shown that the RR associated with SBP declines with age, with the log (RR) having an approximately linear relationship with age and reaching a value of 1 between the ages of 100 and 120. RRs were reported per 10 mm Hg increase in SBP above TMREL value (115 mm Hg) as in the equation below:

$$RR_x = RR^{(x-TMREL)}$$

We used Dismod-MR 2.1 to pool effect sizes from included studies and generate a dose-response curve for each of the outcomes associated with high SBP. The tool enabled us to incorporate random effects across studies and include data with different age ranges. RRs were used universally for all countries and the meta-regression only helped to pool the three major sources and produce RRs with uncertainty and covariance across ages taking into account the uncertainty of the data points.

F.2. High total blood cholesterol

For the purpose of attributing disease burden to high total blood cholesterol, the TMREL for total cholesterol was estimated to range from 50 to 61 mg/dL or 2.8 to 3.4 mmol/L. A meta-analysis of randomized statin trials has shown that adverse health outcomes can be caused even at low levels of LDL-cholesterol, above 1.3 mmol/L (J Am Coll Cardiol 2014; 64: 485–94). A recent outcome study on new lipid-lowering biologic agents (N Engl J Med 2017; 376:1713-1722) also support these results. Since these studies reported LDL-cholesterol and not total cholesterol, we used the strong correlation between LDL-cholesterol and total cholesterol to map the proposed TMREL for LDL-cholesterol of 0.7-1.3 mmol/L to a TMREL for total cholesterol of 2.8-3.4 mmol/L. This TMREL is much lower than the clinical value of total cholesterol that is considered abnormal, but seems justified in light of the evidence that the total cholesterol levels above this TMREL are associated with adverse outcomes. To include the uncertainty in the TMREL, we took a random draw from the uniform distribution of the interval between 2.8 mmol/L and 3.4 mmol/L each time the population attributable burden was calculated.

The steps in the estimation of disease burden attributable to high total blood cholesterol are shown in the following flowchart:

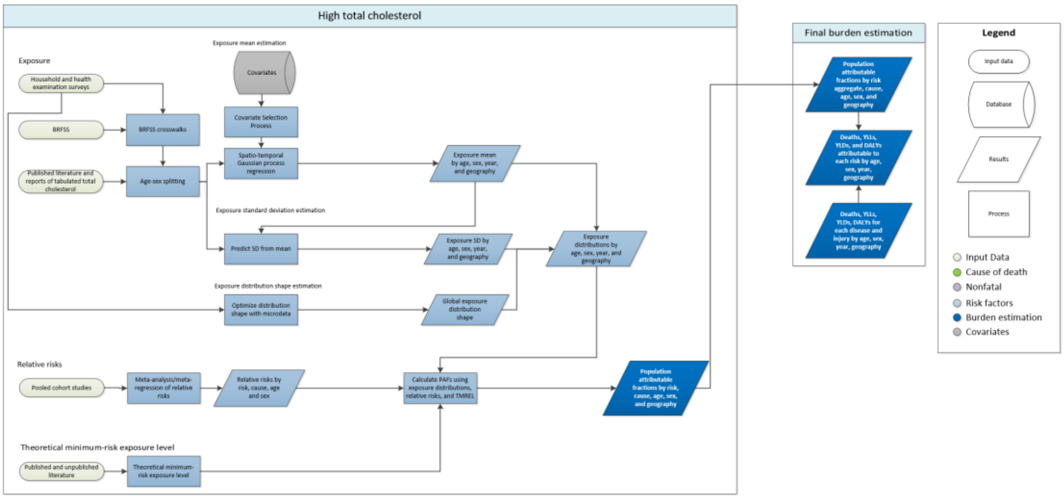

Data

We utilised data on blood mean total cholesterol from literature and from household survey microdata and reports. Studies were included if they were population-based and measured total blood cholesterol using a blood test. We assumed the data were representative of the location if the geography was not selected because it was related to the diseases associated with high cholesterol.

Data were utilised in the modelling process unless an assessment of data strongly suggested that the data were biased. A candidate source was excluded if the quality of study did not warrant a valid estimate because of selection (specific populations), different definitions, or if the study did not provide methodological details for evaluation. In a small number of cases, data point was considered to be an outlier candidate if the level was widely inconsistent with other country data.

Where possible, individual level data on total blood cholesterol estimates were extracted from survey microdata and these were collapsed across individuals and collapsed across demographic groupings to produce mean estimates in the standard GBD 5-year age-sex groups. If microdata were unavailable, information from survey reports or from literature were extracted along with any available measure of uncertainty including standard error, uncertainty intervals, and sample size. Standard deviations were also extracted.

Prior to modelling, data provided in age groups wider than the GBD 5-year age groups were split using a standard approach. Briefly, age-sex patterns were identified using sources of data with multiple age-sex groups and these patterns were applied to split aggregated report data.

Modelling strategy

Exposure estimates were produced from 1980 to 2016 for each national and subnational location, sex, and for each 5-year age group starting from 25+. We used a ST-GPR framework to model the mean total blood cholesterol at the location-, year-, age-, sex- level.

The first step of the ST-GPR framework requires the creation of a linear model for predicting total cholesterol at the location-, year-, age-, sex- level. Covariates for this model were selected in two stages. First, a list of variables with an expected causal relationship with total cholesterol was created based on significant association found within high-quality prospective cohort studies reported in the published scientific literature. These variables were: dietary fibre availability, dietary fruit availability, dietary polyunsaturated fatty acid (PUFA) availability, dietary nuts and seeds availability, and the prevalence of overweight persons in a population. We also used the SDI, and the health access quality index (HAQI) to represent the effect of proximal socioeconomic factors and access to health care on exposure levels. The second stage in covariate selection was to test the predictive validity of every possible combination of covariates in the linear model, given the covariates selected above. This was done separately for each sex. Predictive validity was measured with out of sample root-mean-squared error. The linear model with the lowest root-mean squared error for each sex was then used in the ST-GPR model. For women, this linear model was:

$$\log(\mathrm{TC}_{c,a,t}) = \beta_0 + \beta_1 \mathrm{SDI}_{c,t} + \beta_2 \mathrm{HAQI}_{c,a,t} + \beta_3 \mathrm{nuts}_{c,a,t} + \beta_4 \mathrm{fibre}_{c,a,t} + \sum_{k=2}^{16} \beta_k I_{A[a]} + \alpha_s + \alpha_r + \epsilon_{c,a,t}$$

For men, the linear model was:

$$\log(\mathrm{TC}_{c,a,t}) = \beta_0 + \beta_1 \mathrm{SDI}_{c,t} + \beta_2 \mathrm{HAQI}_{c,a,t} + \beta_4 \mathrm{prev\_overweight}_{c,a,t} + \beta_3 \mathrm{PUFA}_{c,a,t} + \beta_4 \mathrm{fibre}_{c,a,t} + \sum_{k=2}^{16} \beta_k I_{A[a]} + \alpha_s + \alpha_r + \epsilon_{c,a,t}$$

where $\mathrm{SDI}_{c,t}$ is socio-demographic index, an index metric that includes a measure of education, fertility, and income, HAQI is the health access quality index, $\mathrm{prev\_overweight}_{c,a,t}$ is the prevalence of overweight, $\mathrm{nuts}_{c,a,t}$ is the calorie adjusted food availability of nuts and seeds, $\mathrm{PUFA}_{c,a,t}$ is the calorie adjusted food availability of poly-unsaturated fatty acids per capita per day, $\mathrm{fibre}_{c,a,t}$ is the calorie adjusted food availability of fibre, $I_{A[a]}$ is a dummy variable for a fixed effect on a given 5-year age group, and α_s and α_r are random effects at the super-region and region level, respectively. The table below contains the coefficients of the fixed effects used in the two regressions.

Covariate	Male	Female
Fibre	-0.00287 (-0.0034 to -0.0022)	-0.00196 (-0.0025 to -0.0014)
Nuts/seeds	NA	-0.00364 (-0.004 to -0.003)
SDI	0.317 (0.278 to 0.356)	0.307 (0.271 to 0.343)
HAQI	-0.0016 (-0.0021 to -0.0012)	-0.0008 (-0.0011 to -0.0004)
PUFA	-0.533 (-0.719 to -0.347)	NA
Prevalence of overweight	0.0364 (0.0138 to 0.0591)	NA

The estimates were then propagated through the ST-GPR framework to obtain 1000 draws for each location, year, age, and sex.

The standard deviation of total cholesterol within a population was estimated for each national and subnational location, sex, and 5-year age group starting from age 25. The shape of the distribution of total cholesterol was estimated for each age group using all available person-level microdata sources, which was a subset of the input data into the modelling process.

We used Dismod-MR 2.1 to pool effect sizes from included studies and generate a dose-response curve for each of the outcomes associated with high total cholesterol. The tool enabled us to incorporate random effects across studies and include data with different age ranges. RRs were used universally for all countries and the meta-regression only helped to pool the three major sources and produce RRs with uncertainty and covariance across ages taking into account the uncertainty of the data points.

F.3. High fasting plasma glucose

Fasting plasma glucose (FPG) level is used to define diabetes (FPG more than 126 mg/dL or 7 mmol/L), and FPG is also a risk factor for other disease conditions.

For the purpose of attributing disease burden to FPG, the TMREL for FPG was estimated to range from 81 to 97 mg/dL or 4.5 to 5.4 mmol/L as a risk of chronic kidney disease, ischaemic heart disease, stroke, and peripheral vascular disease. Above this FPG level, the risk was considered continuous. Based on the relative risks obtained from meta-analysis, FPG level more than 126 mg/dL (7 mmol/L) was considered as a categorical risk for tuberculosis, liver cancer, pancreatic cancer, ovarian cancer, colorectal cancer, bladder cancer, lung cancer, breast cancer, glaucoma, cataract, and Alzheimer’s disease and other dementias. This was calculated by taking the person-year weighted average of the levels of FPG that were associated with the lowest risk of mortality in the pooled analyses of prospective cohort studies. To include the uncertainty in the TMREL, we took a random draw from the uniform distribution of the interval between 4.5 mmol/L and 5.4 mmol/L each time the population attributable burden was calculated.

Morbidity and mortality directly caused by diabetes was considered directly attributable to FPG.

The steps in the estimation of disease burden attributable to high FPG are shown in the following flowchart.

High fasting plasma glucose

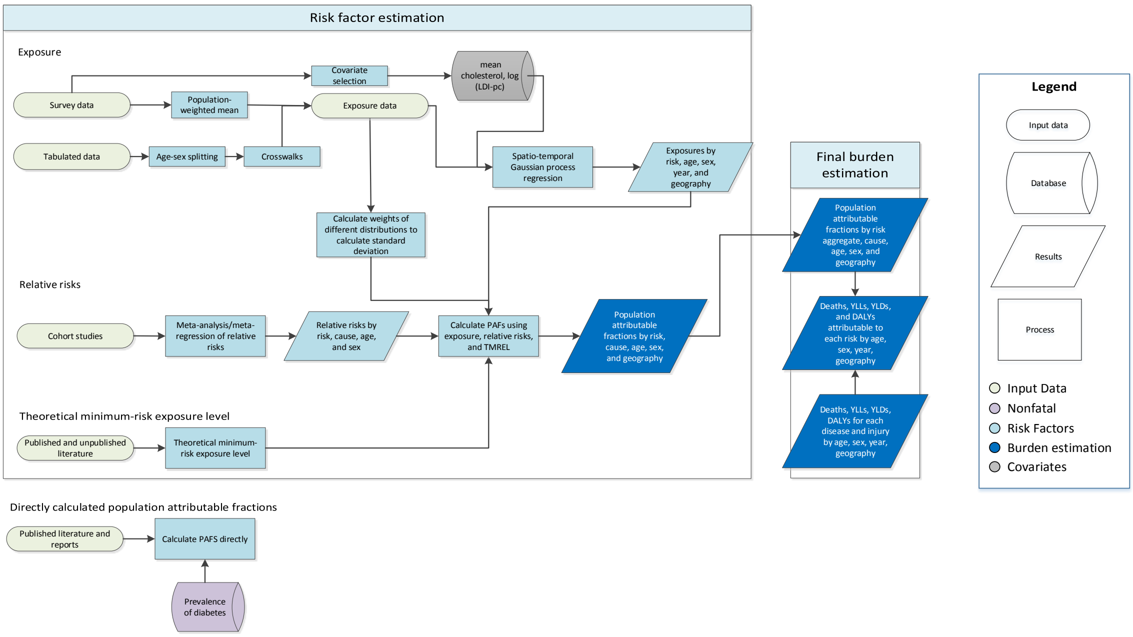

Data

The data inputs derived from estimates of mean FPG in a representative population, individual-level data of fasting plasma glucose measured from surveys, and estimates of diabetes prevalence in a representative population. Data sources that did not report mean FPG or prevalence of diabetes were excluded from analysis. When a study reported both mean fasting plasma glucose (FPG) and prevalence of diabetes, we used the mean FPG for exposure estimates. Where possible, individual-level data superseded any data described in a study. Individual-level data was collapsed and aggregated to produce estimates for each age group, sex, location, and year a survey is conducted.

We perform several processing steps to the data in order to address sampling and measurement inconsistencies that will ensure the data are comparable.

1. Small sample size: Estimates in a sex and age group with a sample size <30 persons was considered a small sample size. In order to avoid small sample size problems that may bias estimates, data were collapsed into the next age group in the same study till the sample size reached at least 30 persons. The intent of collapsing the data is to preserve as much granularity between age groups as possible which determined whether the collapse occurred with a younger or older age group. If the entire study sample consisted of <30 persons and did not include a population-weight, the study was excluded from the modelling process. The estimates were re-calculated if case count and sample size were available or the population-weighted estimate was calculated when only sample size was available.

2. Time, age, and sex splitting: More details on how data on mean FPG were processed are available in the GBD 2016 non-fatal capstone paper (Lancet 2017; 390: 1211–59).

3. Crosswalks: We predicted mean FPG from diabetes prevalence using an ensemble distribution. We characterised the distribution of FPG using individual-level data. For more details on the ensemble distribution, please see the GBD 2016 risk factors paper (Lancet 2017; 390: 1345–422). Before predicting mean FPG from prevalence of diabetes, we ensured that the prevalence of diabetes was based on the reference case definition: fasting plasma glucose (FPG) > 126 mg/dL (7 mmol/L) or on treatment.

Modelling strategy

Exposure estimates were produced from 1980 to 2016 for each national and subnational location, sex, and for each 5-year age group starting from 25+. We used a ST-GPR framework to model the mean fasting plasma glucose at the location-, year-, age-, sex- level.

Fasting plasma glucose is frequently tested or reported in surveys aiming at assessing the prevalence of diabetes mellitus. In these surveys, the case definition of diabetes may include both a glucose test and questions about treatment for diabetes; people with positive history of diabetes treatment are generally excluded from the FPG test. Thus, the mean FPG in these surveys may not represent the mean FPG in the entire population. To address this limitation, using the data from the surveys reporting mean FPG in the entire population, we estimated a regression-based correction factor and adjusted the mean FPG to account for diabetics in the population. We also used an ensemble distribution to characterize the distribution of FPG in the population and developed an optimization function to estimate the standard deviation based on mean FPG and prevalence of diabetics.

To inform our estimates in data-sparse countries, we systematically tested a range of covariates and selected two covariates based on AIC and adjusted R^2. These included prevalence of obesity and lag distributed income per capita (LDI).

Mean FPG was estimated using a mixed-effects linear regression, run separately by sex:

$$\log\left(\mathrm{FPG}_{c,a,t}\right) = \beta_0 + \beta_1 \log(\mathrm{LDI})_{c,t} + \beta_2 Poverweight_{c,a,t} + \sum_{k=2}^{16} \beta_k I_{A[a]} + \alpha_s + \alpha_r + \alpha_c + \epsilon_{c,a,t}$$

where log $(\mathrm{LDI})_{c,t}$ is the log of the lag-distributed income, P overweight $_{c,a,t}$ is the prevalence of overweight, IA[a] is an indicator variable for a fixed effect on a given 5-year age group, and αs αr αc are random effects at the super-region, region, country, and subnational level, respectively.

The estimates were then propagated through the ST-GPR framework to obtain 1000 draws for each location, year, age, and sex.

We used Dismod-MR 2.1 to pool effect sizes from included studies and generate a dose-response curve for each of the outcomes associated with high fasting plasma glucose. The tool enabled us to incorporate random effects across studies and include data with different age ranges. RRs were used universally for all countries and the meta-regression only helped to pool the three major sources and produce RRs with uncertainty and covariance across ages taking into account the uncertainty of the data points.

F.4. Smoking

For the purpose of attributing disease burden to smoking, the TMREL was all individuals who were lifelong non-smokers, above which there could be adverse health effects.

The steps in the estimation of disease burden attributable to smoking are shown in the following flowchart:

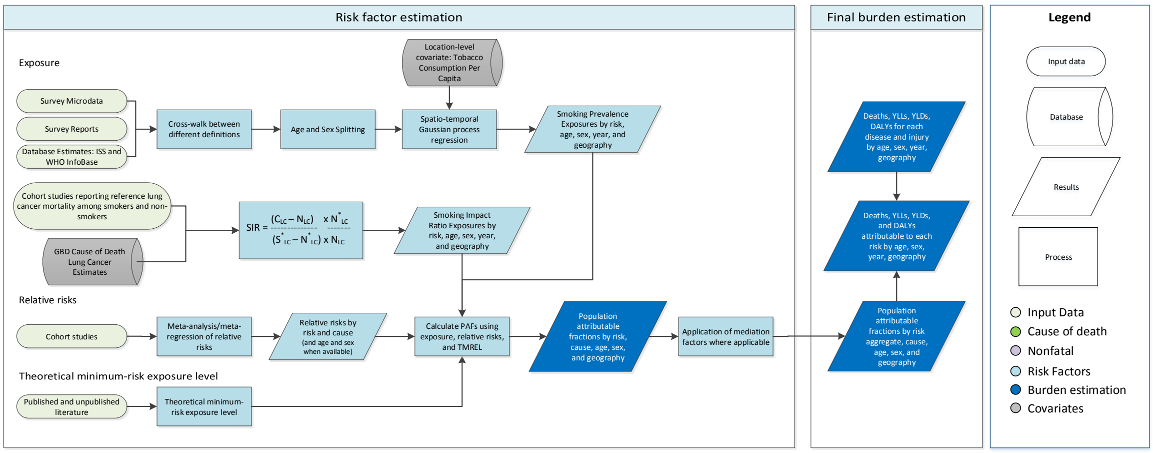

Data

We included representative survey data sources that captured information on primary tobacco use among individuals over age 10. We included only self-reported smoking data and excluded data from questions asking about others' smoking behaviours. In addition to the primary data sources, we used secondary database estimates from the WHO InfoBase Database and International Smoking Statistics Database for sources for which primary data are unavailable.

We extracted primary data from individual-level microdata and survey report tabulations. We extracted data on current smoked tobacco use reported as any combination of frequency of use (daily, occasional, and current, which includes both daily and occasional smokers), type of smoked tobacco used (all smoked tobacco, cigarettes, hookah, and other smoked tobacco products such as cigars or pipes), and whether the data included only current smokers, only former smokers, or both current and former smokers, resulting in 36 possible combinations.

For microdata, we extracted relevant demographic information, including age, sex, location, and year, as well as survey metadata, including survey weights, primary sampling units, and strata. This information allowed us to tabulate individual-level data in the standard GBD five-year age-sex groups and produce accurate estimates of uncertainty. For survey report tabulations, we extracted data at the most granular age-sex group provided.

Crosswalking: Our case-definition for smoking prevalence is current daily use of any smoked tobacco products. All other data points were adjusted to be consistent with this definition. Some sources contained information on more than one indicator and these sources were used to develop the adjustment coefficient to transform that alternative definitions to the GBD standard case-definition of daily use of smoked tobacco. The adjustment coefficient was the beta value derived from the following model:

$$p_{daily-smoked,k} = \beta p_{i,k} + \epsilon_k$$

where $p_{daily-smoked,k}$ is the prevalence of daily smoking reported in survey k and $p_{i,k}$ is the prevalence of an alternative frequency-type combination i also reported in survey k. Models with adjusted R-squared values > 0.8 were used in order of their R-squared value.

We propagated uncertainty at the survey (k*)* level from the crosswalk using the following equation:

$$PE_k = \sigma_\epsilon^2 + X_k^2 var(\hat{\beta})$$

where PE_k is the crosswalk prediction error that is added to the sampling variance of the data point, σ_ϵ^2 is the variance of the error, X_k^2 is the squared value of the data being adjusted, and $var(\hat{\beta})$ is the variance of the adjustment coefficient.

We split data reported in broader age groups than the GBD 5-year age groups or as both sexes combined using a standard approach.

Modelling strategy

We used ST-GPR to model smoking prevalence given the abundance of age and sex-specific data. Briefly, the mean function input to GPR is a complete time series of estimates generated from a mixed effects hierarchical linear model plus weighted residuals smoothed across time, space, and age. The linear model formula, fit separately by sex using restricted maximum likelihood in R, is:

$$logit(p_{g,a,t}) = \beta_0 + \beta_1 CPC_{g,t} + \sum_{k=2}^{19} \beta_k I_{A[a]} + \alpha_s + \alpha_r + \alpha_g + \epsilon_{g,a,t}$$

where $CPC_{c,t}$ is the tobacco consumption covariate, by geography g and time t, described above, $I_{A[a]}$ is a dummy variable indicating specific age group A that the prevalence point $p_{g,a,t}$ captures, and α_s, α_r, and α_g are super region, region, and geography random intercepts, respectively. Random effects were used in model fitting but were not used in prediction.

We used out-of-sample cross validation for hyperparameter selection for the space (zeta), age (omega), and time (lambda) weights used in spatiotemporal smoothing along with the scale used in Gaussian process regression (details on the effects of different parameters have been previously published). We used a space weight of 0.95 in data-dense countries (at least five years covered in a geography-age-sex group) and space weight of 0.7 in data-sparse countries. The other parameters were consistent across data-density levels: age weight = 1, time weight = 1, and scale = 10.

Relative risk estimates were derived from prospective cohort studies. Relative risk estimates and uncertainty for all outcomes associated with smoking were included in analysis, by age and sex as applicable.

F.5. Dietary risks

Dietary risks in GBD comprise of ten components that are protective which include fruits, nuts and seeds, seafood omega-3 fatty acids, vegetables, fibre, whole grains, legumes, polyunsaturated fatty acids, calcium, and milk intake; and five components that are harmful which include sodium, trans-fatty acids, processed meat, sugar sweetened beverages, and red meat intake.

Fruits intake includes consumption of fresh, frozen, cooked, canned, or dried fruits, excluding fruit juices and salted or pickled fruits. Seafood omega-3 fatty acids intake includes consumption of eicosapentaenoic acid (EPA) and docosahexaenoic acid (DHA). Vegetables intake includes consumption of fresh, frozen, cooked, canned or dried vegetables, excluding legumes and salted or pickled vegetables, juices, nuts and seeds, and starchy vegetables such as potatoes or corn. Dietary fibre intake includes consumption of fibre from all sources including fruits, vegetables, grains, legumes and pulses. Whole grains intake includes consumption of bran, germ, and endosperm in their natural proportion from breakfast cereals, bread, rice, pasta, biscuits, muffins, tortillas, pancakes and other sources. Polyunsaturated fatty acids intake was estimated as its average daily percent contribution to the total daily energy intake. Calcium intake includes consumption of calcium from all sources, including milk, yogurt, and cheese. Milk intake includes consumption of milk including non-fat, low-fat, and full-fat milk, excluding soy milk and other plant derivatives.

Trans fatty acids intake includes consumption of trans fat from all sources, mainly partially hydrogenated vegetable oils and ruminant products. Processed meat intake includes consumption of meat preserved by smoking, curing, salting, or addition of chemical preservatives. Sugar sweetened beverages intake includes consumption of carbonated beverages, sodas, energy drinks, fruit drinks, but excluding 100% fruit and vegetable juices. Red meat intake includes consumption of beef, pork, lamb, and goat but excluding poultry, fish, eggs, and all processed meats.

For the purpose of attributing disease burden for each dietary risk, the TMREL for each of these dietary risks was calculated as follows. We first calculated the level of intake associated with the lowest risk of mortality from each disease endpoint based on the studies included in the meta-analyses of the dietary relative risks. Then, we calculated the TMREL as the weighted average of these numbers using the global number of deaths from each of outcome as the weight. Using this approach, the TMREL for each dietary risk was estimated as follows:

**Dietary risks**	**TMREL range**
Fruits	200-300 gm/day
Nuts and seeds	16-25 gm/day
Seafood omega-3 fatty acids	200-300 mg/day
Vegetables	290-430 gm/day
Dietary fibre	19-28 gm/day
Whole grains	100-150 gm/day
Legumes	50-70 gm/day
Polyunsaturated fatty acids	9-13% of total daily energy
Dietary calcium	1.0-1.3 gm/day
Milk	350-520 gm/day

Trans fatty acids	0-1% of total daily energy
Processed meats	0-4 gm/day
Sugar sweetened beverages	0-5 gm/day
Red meats	18-27 gm/day

To include the uncertainty in the TMREL, we took a random draw from the uniform distribution of the interval between the ranges as given in the table above for each dietary risks, each time the population attributable burden was calculated.

The steps in the estimation of disease burden attributable to dietary risks are shown in the following flowchart:

Dietary risks

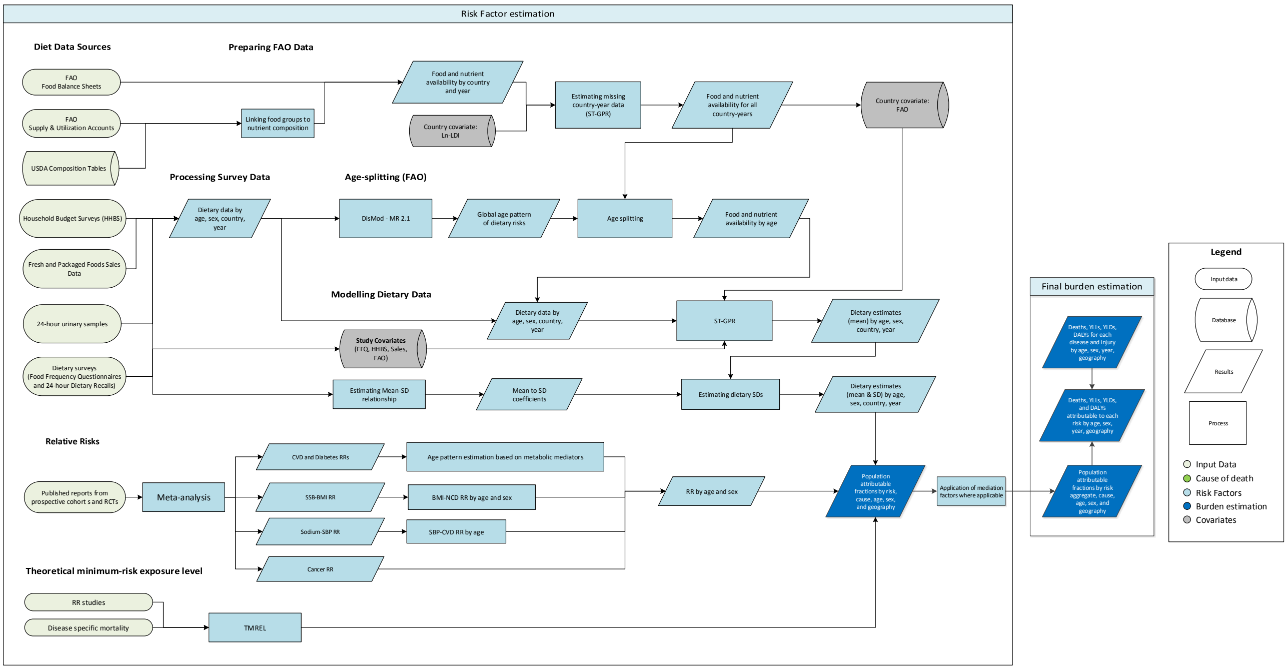

Data

We used dietary data from multiple sources including nationally and sub-nationally representative nutrition surveys, household budget surveys, accounts of national sales, and United Nations FAO Food Balance Sheets and Supply and Utilization Accounts. Additionally, for sodium and trans fatty acids, we used data on 24-hour urinary sodium and availability of partially hydrogenated vegetable oil in packaged foods, respectively. Saturated and trans fatty acids were modelled as a percent of total dietary energy. Data gathered through a systematic review of literature for each of the dietary risks were also used. For all dietary factors other than sodium, we considered data from 24-hour diet recall as the gold standard, and cross-walked other methods of assessment to the gold standard method. For sodium, the 24-hour urinary sodium was considered as the gold standard. To estimate the 24-hour urinary sodium based on dietary sodium, we performed a crosswalk adjustment between these two types of data.

Modelling strategy

Exposure estimates were produced from 1980 to 2016 for each national and subnational location, sex, and for each 5-year age group. We used a ST-GPR framework to estimate the intake of each dietary factor by age, sex, country, and year. We modelled missing country-year data from FAO using a space-time Gaussian process regression and lag-distributed country income as the covariate. For each dietary factor, we estimated the global age pattern of consumption based on nutrition surveys (i.e., 24-hour diet recall) and applied that age pattern to the FAO data. These data were included to account for sales of fruit, vegetables, legumes, processed meats, red meats, sugar-sweetened beverages, and milk.
Table below summarizes the study- and country-level covariates used in modelling of each dietary risk:

	Sex	Suboptimal metric	Nationally Representativeness	Data from FFQ[1]	Data from HBS[2]	Data from FAO	**Country level covariate**
Diet low in fruits	●	●	●	●	●	●	Lag-distributed income
Diet low in vegetables	●	●	●	●	●	●	Lag-distributed income
Diet low in legumes	●	●	●	●	●	●	Lag-distributed income
Diet low in whole grains	●	●	●	●	●	-	Energy adjusted fibre availability (grams/person/day)
Diet low in nuts and seeds	●	●	●	●	●	●	Lag-distributed income
Diet low in milk	●	●	●	●	●	●	Lag-distributed income
Diet high in red meat	●	●	●	●	●	●	Lag-distributed income
Diet high in processed meat	●	●	●	●	●	-	National availability of red meat (grams/person/day)<br>National availability of pig meat (% of energy/person/day)
Diet high in sugar-sweetened beverages	●	●	●	●	●	-	National availability of sugar (Kcal/person/day)
Diet low in fibre	●	●	●	●	●	●	Lag-distributed income
Diet suboptimal in calcium	●	●	●	●	●	●	Lag-distributed income
Diet low in seafood omega-3 fatty acids	●	●	●	●	●	●	Landlocked nation (Yes/No),<br>Lag-distributed income
Diet low in polyunsaturated fatty acids	●	●	●	●	●	●	Lag-distributed income
Diet high in trans fatty acids	●	●	●	●	●	-	National availability of hydrogenated oil (% of energy/person/day)
Diet high in sodium	●		●	-	-	-	-

[1] Food Frequency Questionnaire. [2] Household Budge Survey.

To characterize the distribution of each dietary factor at population level, we used the following equation to individually model the relationship between the standard deviation and mean of intake in nationally representative nutrition surveys using multiple 24-hour diet recalls:

$$ln\ (Standard\ deviation) = \beta_0 + \beta_1 \times ln\ (Mean_i)$$

Then we applied the coefficients of this regression to the outputs of our ST-GPR model to calculate the standard deviation of intake by age, sex, year, and country.

We obtained the relative risk of each disease endpoint per serving of the dietary components from the most recent dose-response meta-analyses of prospective observational studies, and available randomized controlled trials. The relative risks for the relationship between a diet low in legumes and ischaemic heart disease, is now being considered distinctly as opposed to being placed within the category of vegetables. For each dietary risk, we searched for randomized trials evaluating the effect of the diet component on metabolic risks and estimated the change in a given mediator per unit change in the diet component. Considering the well-established age trend of the relative risks of metabolic risks for cardiovascular disease and diabetes, we conducted a literature review to identify the most important metabolic mediators for each dietary factor and used the age trend of the relative risk of that mediator(s) and the disease endpoint to estimate the age-specific relative risk for each dietary factors which is presented in the table below.

	Body mass index	Total serum cholesterol	Fasting plasma glucose	Systolic blood pressure
Diet low in fruits	●	●	●	●
Diet low in vegetables	●	●	●	●
Diet low in legumes	●	●	●	●
Diet low in whole grains	●	●	●	-
Diet low in nuts and seeds	●	●	●	●
Diet high in red meats	●	-	●	-
Diet high in processed meats	●	-	●	●
Diet low in fibre	-	●	-	-
Diet low in seafood omega-3 fatty acids	●	-	-	●
Diet low in polyunsaturated fatty acids	-	●	●	-
Diet high in trans fatty acids	●	●	-	-

We used Dismod-MR 2.1 to pool effect sizes from included studies and generate a dose-response curve for each of the outcomes associated with high fasting plasma glucose. The tool enabled us to incorporate random effects across studies and include data with different age ranges. RRs were used universally for all countries and the meta-regression only helped to pool the three major sources and produce RRs with uncertainty and covariance across ages taking into account the uncertainty of the data points.

F.6. Air pollution

Air pollution in GBD consists mainly of ambient air pollution and household air pollution. The exposure to these and the disease burden caused by them are estimated separately in GBD.

F.6.1. Ambient particulate matter pollution

Exposure to ambient air pollution for this GBD analysis was defined as the population-weighted annual average mass concentration of particles with an aerodynamic diameter less than 2.5 micrometers ($PM_{2.5}$) in a cubic meter of air at a spatial resolution of approximately 11 x 11 km. This measurement was reported in $\mu g/m^3$.

For the purpose of attributing disease burden to ambient air pollution, the theoretical minimum-risk exposure level for ambient air pollution was defined as population-weighted mean between 2.4 and 5.9 $\mu g/m^3$, bounded by the minimum and fifth percentiles of exposure distributions from outdoor air pollution cohort studies. The uniform distribution represents the uncertainty regarding adverse effects of low-level exposure.

The steps in the estimation of disease burden attributable to ambient air pollution are shown in the following flowchart:

Ambient PM2.5

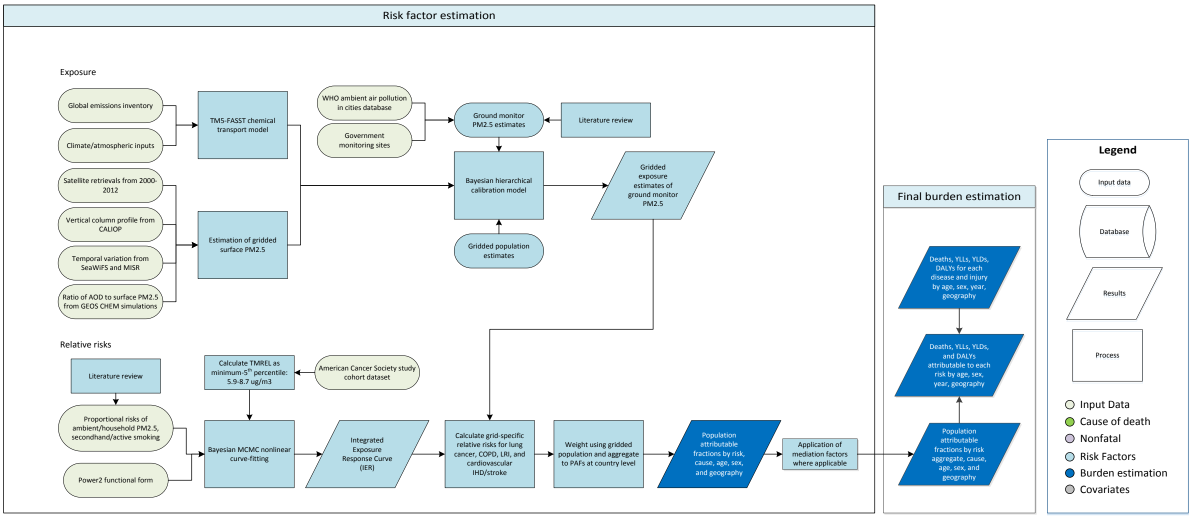

Data

The estimates of ambient $PM_{2.5}$ exposures in India were based on multiple satellite-based aerosol optical depth data combined with a chemical transport model, and calibration of these with $PM_{2.5}$ data from the ground-level monitoring stations.

$PM_{2.5}$ ground measurements: Monitor-specific measurements (rather than city averages as reported in the WHO Air Pollution in Cities database) were used, resulting in measurements of concentrations of PM_{10} and $PM_{2.5}$ from over 6,000 ground monitors from 117 countries. For locations measuring only PM_{10}, $PM_{2.5}$ measurements were estimated from PM_{10}. This was performed using a locally derived conversion factor ($PM_{2.5}/PM_{10}$ ratio, for stations where measurements are available for the same year) that was estimated using population-weighted averages of location-specific conversion factors for the country or state. If country-level conversion factors were not available, the average of country-level conversion factors within a region were used. Additional information related to the ground measurements was also included where available, including monitor geo coordinates and monitor site type.

Satellite-based estimates: These estimates were available at 0.1o×0.1o resolution (~11 x 11 km resolution at the equator) and combine aerosol optical depth retrievals from multiple satellites with the GEOS Chem chemical transport model and land use information.

Population data: A comprehensive set of population data on a high-resolution grid was obtained from the Gridded Population of the World (GPW) database. These data are provided on a 0.0417×0.0417° resolution. Aggregation to each 0.1×0.1° grid cell comprised of summing the central 3 × 3 population cells. As this resulted in a resolution higher than necessary, it was repeated four times, each offset by one cell in a North, South, East and West direction. The average of the resulting five quantities was used as the estimated population for each grid cell.

Chemical transport model simulations: Estimates of the sum of particulate sulfate, nitrate, ammonium and organic carbon and the compositional concentrations of mineral dust simulated using the GEOS Chem chemical transport model, and a measure combining elevation and the distance to the nearest urban land surface were available for 2000 to 2015 for each 0.1×0.1° grid cell.

Modelling Strategy

Global annual mean exposure to $PM_{2.5}$ was estimated in 5-year intervals from 1990 to 2015, at 0·1 × 0·1° (~11 km × 11 km at the equator) resolution using estimates from satellites combined with a chemical transport model, surface measurements, and geographical data. We aggregated gridded exposure concentrations to national-level population-weighted means using the corresponding grid cell population

value. National-level population-weighted mean concentrations and the 95% uncertainty interval (95% UI) around this mean were estimated by sampling 1000 draws of each grid cell value and its uncertainty distribution. We used a chemical transport model to calculate a running 3-month mean.

The Data Integration Model for Air Quality (DIMAQ) was used for ambient air pollution modelling. The coefficients in the calibration model were estimated for each country or state. Where data were insufficient within a country or state, information can be `borrowed' from a higher aggregation (region) and if enough information is still not available from an even higher level (super region). Individual country level estimates were therefore based on a combination of information from the country, its region and super-region. This was implemented within a Bayesian Hierarchical Modelling (BHM) framework. BHMs provide an extremely useful and flexible framework in which to model complex relationships and dependencies in data. Uncertainty can also be propagated through the model allowing uncertainty arising from different components, both data sources and models, to be incorporated within estimates of uncertainty associated with the final estimates. The results of the modelling comprise a posterior distribution for each grid cell, rather than just a single point estimate, allowing a variety of summaries to be calculated. The primary outputs here are the median and 95% credible intervals for each grid cell.

Due to both the complexity of the models and the size of the data, notably the number of spatial predictions that are required, recently developed techniques that perform 'approximate' Bayesian inference based on Integrated Nested Laplace Approximations (INLA) were used. Computation was performed using the R interface to the INLA computational engine (R-INLA). Fitting the models and performing predictions for each of the ca. 1.4 million grid cells required the use of a high performance computing cluster (HPC) making use of high memory nodes.

Model development and comparison was performed using within- and out-of-sample assessment. In the evaluation, cross validation was performed using 25 combinations of training (80%) and validation (20%) datasets. Validation sets were obtained by taking a stratified random sample, using sampling probabilities based on the cross-tabulation of $PM_{2.5}$ categories (0-24.9, 25-49.9, 50-74.9, 75-99.9, 100+ $\mu g/m^3$) and super-regions, resulting in them having the same distribution of $PM_{2.5}$ concentrations and super-regions as the overall set of sites. The following metrics were calculated for each training/evaluation set combination: for model fit - R^2 and deviance information criteria (DIC, a measure of model fit for Bayesian models); for predictive accuracy - root mean squared error and population weighted root mean squared error.

All modelling was performed on the log-scale. The choice of which variables were included in the model was made based on their contribution to model fit and predictive ability. The following is a list of variables and model structures that were considered in developing the model:

Variable	Model structure
Continuous explanatory variables	(SAT) Estimate of $PM_{2.5}$ (in µgm-3) for 2014 from satellite remote sensing on the log scale.
	(CTM) Estimate of $PM_{2.5}$ (in µgm-3) for 2010 from the TM5 chemical transport model on the log-scale.
	(POP) Estimate of population for 2014 on the log-scale.
	(SNAOC) Estimate of the sum of sulfate, nitrate, ammonium and organic carbon simulated using the GEOS Chem chemical transport model.
	(DST) Estimate of compositional concentrations of mineral dust simulated using the GEOS Chem chemical transport model.
	(EDxDU) The log of the elevation difference between the elevation at the ground measurement location and the mean elevation within the GEOS Chem simulation grid cell multiplied by the inverse distance to the nearest urban land surface.
Discrete explanatory variables	(LOC) Binary variable indicating whether exact location of ground measurement is known.
	(TYPE) Binary variable indicating whether exact type of ground monitor is known.
	(CONV) Binary variable indicating whether ground measurement is $PM_{2.5}$ or converted from PM_{10}.
Random Effects	Grid cell random effects on the intercept to allow for multiple ground monitors in a grid cell.
	Country-region-super-region hierarchical random effects for the intercept.
	Country-region-super-region hierarchical random effects for the coefficient associated with SAT.
	Country-region-super-region hierarchical random effects for the coefficient associated with the difference between estimates from CTM and SAT.
	Country-region-super-region hierarchical random effects for the coefficient associated with POP.
	Country level random effects for population uses a neighbourhood structure allowing specific borrowing of information from neighbouring countries.

	Within a region, country level effects of SAT and the difference between SAT AND CTM are assumed to be independent and identically distributed.
	Within a super-region, region level random effects are assumed to be independent and identically distributed.
	Super-region random effects are assumed to be independent and identically distributed.
Interactions	Interactions between the binary variables and the effects of SAT and CTM.

The final model contained the following variables: SAT, POP, SNAOC, DST, EDxDU, LOC, TYPE, and CONV, together with interactions between SAT and each of LOC, TYPE and CONV. The model structure contained grid cell random effects on the intercept to allow for multiple ground monitors in a grid cell, country-region-super-region hierarchical random effects for intercepts and SAT and country level random effects for population using a neighbourhood structure allowing specific borrowing of information from neighbouring countries together with region-super-region hierarchical random effects for POP.

DIMAQ showed improved predictions of ground measurements in all super regions. Using this model resulted in an improvement in both within-sample fit; with an increase in R^2 to 0.91, and out-of- sample predictive ability; with a global population-weighted RMSE of 12.1 µg/m3.

Satellite estimates, populations and quantities estimated using the GEOS-Chem model were available for 1990, 1995, 2000, 2005, 2010, 2011, 2012, 2013, 2014 and 2015. Population estimates for 2000, 2005, 2010, 2015 and 2020 were available from GPW version 4. For 1990 and 1995 data were extracted from GPW version 3. As with populations for 2015, values for each cell for 2011, 2012, 2012, 2013 and 2014 were obtained by interpolation using natural splines with knots placed at 2000, 2005, 2010, 2015 and 2020.

These were used as inputs to DIMAQ, enabling estimates of exposures to be obtained for each of these years respectively. For 2016, estimates of exposures were obtained from predictions from locally-varying regression models. For each cell a model was fit to the values within that cell over time, with a constraint placed on the rate of change between 2015 and 2016 to avoid unrealistic and/or unjustified extrapolation of trends. Measures of uncertainty were obtained by repeating the procedure for the limits of the 95% intervals, again on a cell-by-cell basis.

We estimated the burden attributable to $PM_{2.5}$ for ischaemic heart disease (IHD), stroke, lung cancer, COPD, and lower respiratory infections (LRI). Evidence linking these diseases with exposure to ambient air pollution was judged to be consistent with a causal relationship on the basis of criteria specified for GBD risk factors. We developed integrated exposure–response functions (IERs) for each cause of death to estimate the relative risk of mortality over the entire global range of ambient annual mean $PM_{2.5}$ concentrations using risk estimates from studies of ambient air pollution, household air pollution, and second-hand smoke exposure and active smoking. IERs assign concentrations of $PM_{2.5}$ to each type of exposure on an equivalent µg/m³ basis assuming that risk is determined by the 24-h $PM_{2.5}$ inhaled dose regardless of the exposure source. An alternative method to estimate exposure to second-hand smoke was used that incorporated estimates of $PM_{2.5}$ attributable to exposure per cigarette, breathing rate, and number of cigarettes smoked in the country where each study was done.

The IER has the mathematical form:

$$IER(\beta,z)=1+\alpha\times(1-e^{-\beta(z-z_{cf})_+^{\gamma}})$$

where z is the level of $PM_{2.5}$ and z_{cf} is the TMREL, below which no additional risk is assumed, with

$$(z-z_{cf})_+=(z-z_{cf})$$

if z is greater than z_{cf} and zero otherwise. Here, $1+\alpha$ is the maximum risk, β is the ratio of the IER at low to high concentrations, and γ is the power of $PM_{2.5}$ concentrations. Epidemiological evidence suggests that the relative risks for IHD and stroke decline with age. We modified the particulate matter source-specific relative risk for both IHD and stroke mortality and applied this age modification to the relative risks, fitting the IER model for each age group separately. Observed relative risks were related to the IER within a Bayesian framework using the STAN fitting algorithm. Given the true values of the four parameters (α, β, γ, z_{cf}), we assumed that the logarithm of each study's observed relative risk was normally distributed, with mean defined by the IER and variance given by the square of the observed SE of the study-specific log-

relative risk estimate plus an additional variance term for each of the four sources on $PM_{2.5}$ exposure (outdoor air pollution, second-hand smoke, household air pollution, and active smoking).

We calculated 1000 predicted values of the IER for each $PM_{2.5}$ concentration based on the posterior distributions of (α, β, γ) and the prespecified uniform distribution of TMREL to characterise uncertainty in the estimates of the IER. The mean of the 1000 IER predictions at each concentration was used as the central estimate, with uncertainty defined by 95% UIs.

F.6.2. Household air pollution

Exposure to household air pollution from solid fuels (HAP) for this GBD analysis was defined as the proportion of households using solid cooking fuels. The definition of solid fuel in this analysis includes coal, wood, charcoal, dung, and agricultural residues.

The steps in the estimation of disease burden attributable to household air pollution are shown in the following flowchart:

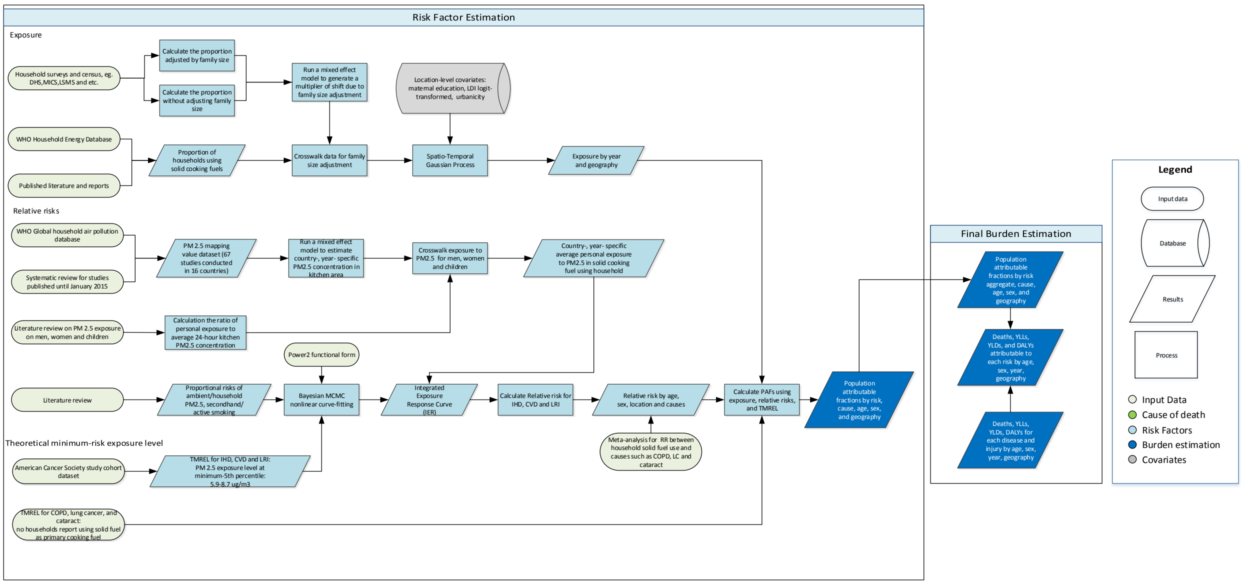

Data

The major data sources on household air pollution from solid fuel use in India include national health surveys such as the National Family Health Survey and the District Level Household Survey, nationwide surveys of the National Sample Survey Organisation, and the Census of India, as well as other published and unpublished epidemiological studies.

Data were extracted from the standard multi-country survey series such as Demographic and Health Surveys (DHS), Living Standards Measurement Surveys (LSMS), Multiple Indicator Cluster Surveys (MICS), and World Health Surveys (WHS), as well as country-specific survey series. To fill the gaps of data in surveys and censuses, we also downloaded and updated HAP estimates from WHO Energy Database and extracted from literature through systematic review. Each nationally or sub-nationally representative data point provided an estimate for the percentage of households using solid cooking fuels. Estimates for the usage of solid fuels for non-cooking purpose were excluded, i.e. primary fuels for lighting.

Modelling strategy

Household air pollution was modelled at household level using a three-step modelling strategy that uses linear regression, spatiotemporal regression and Gaussian Process Regression (GPR). The first step is a mixed-effect linear regression of logit-transformed proportion of households using solid cooking fuels. The linear model contains maternal education, proportion of population living in urban areas, and lagged-

distributed income as covariates and has nested random effect by GBD region, and GBD super region respectively.

A variety of combinations of socioeconomic and environmental covariates in different transformation format were tested by running mixed-effect models with exposure data. The final list of covariates included in the exposure model are maternal education, proportion of population living in urban area, and lagged-distributed income since they proved to be the strongest predictors.

The disease-outcomes paired with household air pollution include LRI, stroke, IHD, COPD, lung cancer and cataract. The relative risks of all outcomes, with the exception of cataracts, were generated by using the IER, as previously described in the section on ambient air pollution modelling. This is done by first estimating the crosswalk values that map household use of solid fuel to $PM_{2.5}$ exposure because the IER curve measures exposure using $PM_{2.5.}$ The average $PM_{2.5}$ exposures from solid fuel use for different household members were derived from studies measuring 24-hour kitchen and living area $PM_{2.5}$ concentrations in households, and estimating this for men, women and children. For outcomes that utilise evidence based on the Integrated Exposure Response, the TMREL was defined as uniform distribution of exposure to $PM_{2.5}$ from solid fuel use between 2.4 and 5.9 ug/m^3. The relative risks for cataracts were extracted from a meta-analysis paper (Annu Rev Public Health 2014; 35: 185–206).

G. Uncertainty intervals

Point estimates for each quantity of interest were derived from the mean of the draws, while 95% uncertainty intervals (UIs) were derived from the 2.5th and 97.5th percentiles of the 1000 draw level values. Uncertainty in the estimation is attributable to sample size variability within data sources, different availability of data by age, sex, year, or location, and cause specific model specifications. We determined UIs for components of cause-specific estimation based on 1000 draws from the posterior distribution of cause specific mortality by age, sex, and location for each year included in the GBD 2016 analysis. Similarly, for non-fatal estimates if there was a change in disease estimates between locations or over time that was in the same direction in more than 950 of the 1000 samples we report it as significant. With this approach, uncertainty could be quantified and propagated into the final quantities of interest.

2. GBD 2016 India data inputs for cardiovascular diseases mortality, morbidity, risk factors, and covariates

Aarhus University, Addiction Switzerland Research Institute, Alcohol Research Group, Public Health Institute, Centre for Addiction and Mental Health, Centre for Alcohol Policy Research, Turning Point Alcohol and Drug Centre, Kettil Bruun Society for Social and Epidemiological Research on Alcohol, University of North Dakota. India - Karnataka Gender, Alcohol and Culture: An International Study (GENACIS) 2003.
Agarwal AK, Yunus M, Ahmad J, Khan A. Rheumatic heart disease in India. J R Soc Promot Health. 1995; 115(5): 303-9.
Ali MK, Bhaskarapillai B, Shivashankar R, Mohan D, Fatmi ZA, Pradeepa R, Masood Kadir M, Mohan V, Tandon N, Narayan KM, Prabhakaran D, CARRS investigators. Socioeconomic status and cardiovascular risk in urban South Asia: The CARRS Study. Eur J Prev Cardiol. 2016; 23(4): 408-19.
All India Institute of Medical Sciences, Bangur Institute of Neurology, Indian Statistical Institute, National Neurosciences Center. Kolkata Study for Epidemiology of Neurological Disorders 2003-2004.
Anand S, Shivashankar R, Ali MK, Kondal D, Binukumar B, Montez-Rath ME, Ajay VS, Pradeepa R, Deepa M, Gupta R, Mohan V, Narayan KM, Tandon N, Chertow GM, Prabhakaran D. Prevalence of chronic kidney disease in two major Indian cities and projections for associated cardiovascular disease. Kidney Int. 2015; 88(1): 178-85.
Banerjee TK, Mukherjee CS, Sarkhel A. Stroke in the urban population of Calcutta – an epidemiological study. Neuroepidemiology. 2001; 20(3): 201-7.
Beegom R, Singh RB. Association of higher saturated fat intake with higher risk of hypertension in an urban population of Trivandrum in South India. Int J Cardiol. 1997; 58(1): 63-70.
Beegom R. Diet, central obesity and prevalence of hypertension in the urban population of South India. Int J Cardiol. 1995; 51(2): 183-91.
Bhagyalaxmi A, Atul T, Shikha J. Prevalence of risk factors of non-communicable diseases in a District of Gujarat, India. J Health Popul Nutr. 2013; 31(1): 78-85.
Bhansali A, Dhandania VK, Deepa M, Anjana RM, Joshi SR, Joshi PP, Madhu SV, Rao PV, Subashini R, Sudha V, Unnikrishnan R, Das AK, Shukla DK, Kaur T, Mohan V, Pradeepa R. Prevalence of and risk factors for hypertension in urban and rural India: the ICMR-INDIAB study. J Hum Hypertens. 2015; 29(3): 204–9.
Bharati DR, Pal R, Kar S, Rekha R, Yamuna TV, Basu M. Prevalence and determinants of diabetes mellitus in Puducherry, South India. J Pharm Bioallied Sci. 2011; 3(4): 513-8.
Bharati DR, Pal R, Rekha R, Yamuna TV. Evaluation of the burden of type 2 diabetes mellitus in population of Puducherry, South India. Diabetes Metab Syndr. 2011; 5(1): 12-6.
Bharati S, Pal M, Bhattacharya BN, Bharati P. Prevalence and causes of chronic energy deficiency and obesity in Indian women. Hum Biol. 2007; 79(4): 395-412.
Bhardwaj R, Kandoria A, Marwah R, Vaidya P, Singh B, Dhiman P, Sharma A. Prevalence of rheumatic fever and rheumatic heart disease in rural population of Himachal--a population based study. J Assoc Physicians India. 2012; 60:13–4.
Bhaya M, Panwar S, Beniwal R, Panwar RB. High prevalence of rheumatic heart disease detected by echocardiography in school children. Echocardiography. 2010; 27(4): 448-53.
Biswas M, Manna CK. Prevalence of hypertension and sociodemographic factors within the Scheduled Caste community of the District Nadia, West Bengal, India. High Blood Press Cardiovasc Prev. 2011; 18(4): 179–85.
Cardiological Society of India. India - Kerala Acute Coronary Syndrome Registry 2007-2009. [Data shared for this analysis]
Cardiological Society of India. India - Kerala Coronary Artery Disease Risk Factors Prevalence Study 2011. [Data shared for this analysis]
Center for Addiction Medicine, National Institute of Mental Health and Neurosciences, Government of India, World Health Organization Collaborative Programme. Report on Patterns and Consequences of Alcohol Misuse in India: An Epidemiological Survey 2011-2012. Bangalore, India: Government of India and World Health Organization; 2013.
Central Board of Secondary Education, Department of School Education and Literacy, Ministry of Human Resource Development, Government of India, Centers for Disease Control and Prevention, World Health Organization (WHO). India Global School-Based Student Health Survey 2007. Geneva, Switzerland: WHO; 2007.
Central Pollution Control Board (CPCB), Ministry of Environment, Forest and Climate Change, Government of India. India - National Air Quality Monitoring Programme Data 2012. New Delhi, India: CPCB.
Central Pollution Control Board (CPCB), Ministry of Environment, Forest and Climate Change, Government of India. India - National Air Quality Monitoring Programme Data 2013. New Delhi, India: CPCB.
Centre for Chronic Disease Control, All India Institute of Medical Sciences, Madras Diabetes Research Foundation, Public Health Foundation of India, Emory University, Aga Khan University. Center for Cardio-Metabolic Risk Reduction in South Asia Surveillance Baseline Survey 2010-2011. [Data shared for this analysis]
Centre for Chronic Disease Control, All India Institute of Medical Sciences, Madras Diabetes Research Foundation, Public Health Foundation of India, Emory University, Aga Khan University. Center for Cardio-Metabolic Risk Reduction in South Asia Surveillance Follow-up Survey 2013. [Data shared for this analysis]

Centre for Chronic Disease Control, Indian Council of Medical Research. India Diet and Lifestyle Interventions for Hypertension Risk reduction through Anganwandi Workers and Accredited Social Health Activists Baseline Study Dataset 2013-2014. [Data shared for this analysis]
Centre for Chronic Disease Control. India Prevalence of Coronary Heart Disease and its Risk Factors in Residents of Urban and Rural Areas of NCR Survey Dataset 2010-2012. [Data shared for this analysis]
Centre for Chronic Disease Control. India Prevalence of Coronary Heart Disease and its Risk Factors in Residents of Urban and Rural Areas of NCR Survey Report 2010-2012. New Delhi, India: Centre for Chronic Disease Control.
Chadha SL, Gopinath N, Shekhawat S. Urban-rural differences in the prevalence of coronary heart disease and its risk factors in Delhi. Bull World Health Organ. 1997; 75(1): 31-8.
Chow C, Cardona M, Raju PK, Iyengar S, Sukumar A, Raju R, Colman S, Madhav P, Raju R, Reddy KS, Celermajer D, Neal B. Cardiovascular disease and risk factors among 345 adults in rural India--the Andhra Pradesh Rural Health Initiative. Int J Cardiol. 2007; 116(2): 180-5.
Christian Medical College - Vellore, MRC Epidemiology Resource Center, University of Southampton. India - Vellore Birth Cohort Study 1998-2002. [Data shared for this analysis]
Christian Medical College - Vellore. India Prevalence of Risk Factors for Non-Communicable Diseases in Rural and Urban Tamil Nadu 2010-2012. [Data shared for this analysis]
Corsi DJ, Subramanian SV, Lear SA, Teo KK, Boyle MH, Raju PK, Joshi R, Neal B, Chow CK. Tobacco use, smoking quit rates, and socioeconomic patterning among men and women: a cross-sectional survey in rural Andhra Pradesh, India. Eur J Prev Cardiol. 2014; 21(10): 1308-18.
Dalal PM, Malik S, Bhattacharjee M, Trivedi ND, Vairale J, Bhat P, Deshmukh S, Khandelwal K, Mathur VD. Population-based stroke survey in Mumbai, India: incidence and 28-day case fatality. Neuroepidemiology. 2008; 31(4): 254-61.
Daniel CR, Prabhakaran D, Kapur K, Graubard BI, Devasenapathy N, Ramakrishnan L, George PS, Shetty H, Ferrucci LM, Yurgalevitch S, Chatterjee N, Reddy KS, Rastogi T, Gupta PC, Mathew A, Sinha R. A cross-sectional investigation of regional patterns of diet and cardio-metabolic risk in India. Open Nutr J. 2011; 10(1): 12.
Das S, Sanyal K, Moitra A. A pilot study on neuroepidemiology in urban Bengal. Indian J Public Health. 1998; 42(2): 34-6.
Das SK, Banerjee TK, Biswas A, Roy T, Raut DK, Mukherjee CS, Chaudhuri A, Hazra A, Roy J. A Prospective Community-Based Study of Stroke in Kolkata, India. Stroke. 2007; 38(3): 906-10.
Das SK, Biswas A, Roy T, Banerjee TK, Mukherjee CS, Raut DK, Chaudhuri A. A random sample survey for prevalence of major neurological disorders in Kolkata. Indian J Med Res. 2006; 124(2): 163-72.
Deedwania PC, Gupta R, Sharma KK, Achari V, Gupta B, Maheshwari A, Gupta A. High prevalence of metabolic syndrome among urban subjects in India: a multisite study. Diabetes Metab Syndr. 2014; 8(3): 156–61.
Deepa M, Anjana RM, Manjula D, Narayan KMV, Mohan V. Convergence of prevalence rates of diabetes and cardiometabolic risk factors in middle and low income groups in urban India: 10-year follow-up of the Chennai Urban Population Study. J Diabetes Sci Technol. 2011; 5(4): 918–27.
Deepa M, Grace M, Binukumar B, Pradeepa R, Roopa S, Khan HM, et al. High burden of prediabetes and diabetes in three large cities in South Asia: The Center for cArdio-metabolic Risk Reduction in South Asia (CARRS) Study. Diabetes Res Clin Pract. 2015 Nov; 110(2):172–82.
Department of Health and Family Welfare, Government of Punjab, Postgraduate Institute of Medical Education & Research Chandigarh, University of Michigan. India - Punjab Noncommunicable Disease Risk Factors Survey 2014-2015. [Data shared for this analysis]
Department of Mental Health and Substance Abuse, World Health Organization (WHO). Alcohol, Gender, and Drinking Problems: Perspectives from Low and Middle Income Countries 1999-2003. Geneva, Switzerland: WHO; 2005.
Department of Women and Child Development, Ministry of Human Resource Development, Government of India, United Nations Children's Fund (UNICEF) - India Country Office. India Summary Report on the Multiple Indicator Cluster Survey 2000. New Delhi, India: UNICEF India Country Office; 2000.
Department of Women and Child Development, Ministry of Human Resource Development, Government of India. India Nutrition Profile Survey 1995-1996. New Delhi, India: Ministry of Human Resource Development.
Desai, Sonalde, Reeve Vanneman, National Council of Applied Economic Research, University of Michigan. India Human Development Survey 2005. Ann Arbor, Michigan: Inter-University Consortium for Political and Social Research.
Dhamija RK, Dhamija SB. Prevalence of stroke in rural community – an overview of Indian experience. J Assoc Physicians India. 1998; 46(4): 351-4.
Directorate of Economics & Statistics and Office of Chief Registrar (Births & Deaths), Government of National Capital Territory of Delhi. Report on Medical Certification of Cause of Deaths in Delhi-2013. New Delhi, India: Directorate of Economics & Statistics and Office of Chief Registrar (Births & Deaths); 2014.
Directorate of Economics and Statistics and Office of Chief Registrar Births and Deaths (Delhi Territory, India). India - Delhi Medical Certification of Cause of Death Report 2012.
Directorate of Economics and Statistics and Office of Chief Registrar Births and Deaths (Delhi Territory, India). India - Delhi Medical Certification of Cause of Death Report 2013.

Directorate of Economics and Statistics and Office of Chief Registrar Births and Deaths (Delhi Territory, India). India - Delhi Report on Medical Certification of Cause of Deaths 2011.
Dyson PA, Anthony D, Fenton B, Matthews DR, Stevens DE, Community Interventions for Health Collaboration. High rates of child hypertension associated with obesity: a community survey in China, India and Mexico. Paediatr Int Child Health. 2014; 34(1): 43–9.
Embassy of the United States - New Delhi, United States Environmental Protection Agency. United States Mission India NowCast Air Quality Data 2014. New Delhi, India: Embassy of the United States.
Esser MB, Gururaj G, Rao GN, Jernigan DH, Murthy P, Jayarajan D, Lakshmanan S, Benegal V, Collaborators Group on Epidemiological Study of Patterns and Consequences of Alcohol Misuse in India. Harms to Adults from Others' Heavy Drinking in Five Indian States. Alcohol Alcohol. 2016; 51(2): 177–85.
Euromonitor International. Euromonitor Passport - Cigarette Statistics 1999-2016. London, United Kingdom: Euromonitor International.
Fall CHD, Sachdev HS, Osmond C, Lakshmy R, Biswas SD, Prabhakaran D, Tandon N, Ramji S, Reddy KS, Barker DJP, Bhargava SK. Adult metabolic syndrome and impaired glucose tolerance are associated with different patterns of BMI gain during infancy: Data from the New Delhi Birth Cohort. Diabetes Care. 2008; 31(12): 2349-56.
Ferri CP, Schoenborn C, Kalra L, Acosta D, Guerra M, Huang Y, Jacob KS, Rodriguez JJL, Salas A, Sosa AL, Williams JD, Liu Z, Moriyama T, Valhuerdi A, Prince MJ. Prevalence of stroke and related burden among older people living in Latin America, India and China. J Neurol Neurosurg Psychiatr. 2011; 82(10): 1074-82.
Food and Agriculture Organization of the United Nations (FAO). FAOSTAT Commodity Balances - Crops Primary Equivalent. Rome, Italy: FAO.
Food and Agriculture Organization of the United Nations (FAO). FAOSTAT Food Balance Sheets, October 2014. Rome, Italy: FAO.
Gajalakshmi V, Peto R, Kanimozhi VC, Whitlock G, Veeramani D. Cohort Profile: the Chennai prospective study of mortality among 500,000 adults in Tamil Nadu, South India. Int J Epidemiol. 2007; 36(6): 1190-5.
Geetha L, Deepa M, Anjana RM, Mohan V. Prevalence and clinical profile of metabolic obesity and phenotypic obesity in Asian Indians. J Diabetes Sci Technol. 2011; 5(2): 439–46.
Gemmy Cheung CM, Li X, Cheng C-Y, Zheng Y, Mitchell P, Wang JJ, Jonas JB, Nangia V, Wong TY. Prevalence and risk factors for age-related macular degeneration in Indians: a comparative study in Singapore and India. Am J Ophthalmol. 2013; 155(4): 764–73.
Ghosh A, Bala SK. Anthropometric, body composition, and blood pressure measures among rural elderly adults of Asian Indian origin: the Santiniketan aging study. J Nutr Gerontol Geriatr. 2011; 30(3): 305–13.
Ghosh S, Samanta A, Mukherjee S. Patterns of alcohol consumption among male adults at a slum in Kolkata, India. J Health Popul Nutr. 2012; 30(1): 73–81.
Global Burden of Disease Health Financing Collaborator Network, Institute for Health Metrics and Evaluation (IHME). Global Development Assistance for Health, Government, Prepaid Private, and Out-of-Pocket Health Spending 1995-2014. Seattle, United States: IHME; 2017.
Goswami AK, Gupta SK, Kalaivani M, Nongkynrih B, Pandav CS. Burden of Hypertension and Diabetes among Urban Population Aged ≥ 60 years in South Delhi: A Community Based Study. J Clin Diagn Res. 2016; 10(3): LC01-05.
Goudar SS, Goco N, Somannavar MS, Vernekar SS, Mallapur AA, Moore JL, Wallace DD, Sloan NL, Patel A, Hibberd PL, Koso-Thomas M, McClure EM, Goldenberg RL. Institutional deliveries and perinatal and neonatal mortality in Southern and Central India. Reprod Health. 2015; 12(Suppl 2): S13.
Government Medical College - Thiruvananthapuram, Ananthapuri Hospitals and Research Institute, Cosmopolitan Hospital, Jubilee Memorial Hospital. India - Trivandrum Heart Failure Registry 2013. [Data shared for this analysis]
Gupta A, Gupta R, Sarna M, Rastogi S, Gupta VP, Kothari K. Prevalence of diabetes, impaired fasting glucose and insulin resistance syndrome in an urban Indian population. Diabetes Res Clin Pract. 2003; 61(1): 69-76.
Gupta I, Gupta ML, Parihar A, Gupta CD. Epidemiology of rheumatic and congenital heart diseases in school children. J Indian Med Assoc. 1992; 90(3): 57-9.
Gupta R, Deedwania PC, Achari V, Asirvatham AJ, Bhansali A, Gupta A, Gupta B, Gupta S, Jali MV, Mahanta TG, Maheshwari A, Saboo B, Singh J. India Heart Watch Study 2005-2009. [Data shared for this analysis]
Gupta R, Gupta VP, Ahluwalia NS. Educational status, coronary heart disease, and coronary risk factor prevalence in a rural population of India. BMJ. 1994 Nov 19; 309(6965):1332–6.
Gupta R, Gupta VP, Sarna M, Bhatnagar S, Thanvi J, Sharma V, et al. Prevalence of coronary heart disease and risk factors in an urban Indian population: Jaipur Heart Watch-2. Indian Heart J. 2002 Feb;54(1):59–66.
Gupta R, Gupta VP, Sarna M, Prakash H, Rastogi S, Gupta KD. Serial epidemiological surveys in an urban Indian population demonstrate increasing coronary risk factors among the lower socioeconomic strata. J Assoc Physicians India. 2003 May; 51:470–7.
Gupta R, Lodha S, Sharma KK, Sharma SK, Gupta S, Asirvatham AJ, et al. Evaluation of statin prescriptions in type 2 diabetes: India Heart Watch-2. BMJ Open Diabetes Res Care. 2016;4(1): e000275.

Gupta R, Misra A, Vikram NK, Kondal D, Gupta SS, Agrawal A, Pandey RM. Younger age of escalation of cardiovascular risk factors in Asian Indian subjects. BMC Cardiovasc Disord. 2009; 9:28.
Gupta R, Pandey RM, Misra A, Agrawal A, Misra P, Dey S, Rao S, Menon VU, Kamalamma N, Vasantha Devi KP, Revathi K, Vikram NK, Sharma V, Guptha S. High prevalence and low awareness, treatment and control of hypertension in Asian Indian women. J Hum Hypertens. 2012; 26(10): 585–93.
Gupta R, Prakash H, Majumdar S, Sharma S, Gupta VP. Prevalence of coronary heart disease and coronary risk factors in an urban population of Rajasthan. Indian Heart J. 1995 Aug; 47(4):331–8.
Gupta R, Sarna M, Thanvi J, Rastogi P, Kaul V, Gupta VP. High prevalence of multiple coronary risk factors in Punjabi Bhatia community: Jaipur Heart Watch-3. Indian Heart J. 2004; 56(6): 646-52.
Gupta R, Sharma AK, Gupta VP, Bhatnagar S, Rastogi S, Deedwania PC. Increased variance in blood pressure distribution and changing hypertension prevalence in an urban Indian population. J Hum Hypertens. 2003; 17(8): 535-40.
Gupta R, Sharma KK, Gupta BK, Gupta A, Saboo B, Maheshwari A, et al. Geographic epidemiology of cardiometabolic risk factors in middle class urban residents in India: cross-sectional study. J Glob Health. 2015 Jun;5(1):010411.
Gupta R. India - Jaipur Heart Watch Study Data on Blood Pressure, Cholesterol, BMI, and Fasting Blood Glucose 1993-2001. [Data shared for this analysis]
Harikrishnan S, Sanjay G, Anees T, Viswanathan S, Vijayaraghavan G, Bahuleyan CG, et al. Clinical presentation, management, in-hospital and 90-day outcomes of heart failure patients in Trivandrum, Kerala, India: the Trivandrum Heart Failure Registry. Eur J Heart Fail. 2015; 17(8):794–800.
Healis-Sekhsaria Institute for Public Health, Madhya Pradesh Voluntary Health Association, Cancer Foundation of India, School of Preventive Oncology, University of Waterloo. International Tobacco Control Policy Evaluation Project: India Tobacco Control Survey 2010-2011. Navi Mumbai, India and Waterloo, Canada: Healis-Sekhsaria Institute for Public Health and University of Waterloo.
Huffman MD, Prabhakaran D, Osmond C, Fall CHD, Tandon N, Lakshmy R, Ramji S, Khalil A, Gera T, Prabhakaran P, Biswas SKD, Reddy KS, Bhargava SK, Sachdev HS, New Delhi Birth Cohort. Incidence of cardiovascular risk factors in an Indian urban cohort results from the New Delhi birth cohort. J Am Coll Cardiol. 2011; 57(17): 1765-74.
Indian Council of Medical Research (ICMR), Madras Diabetes Research Foundation. Indian Council of Medical Research India Diabetes Study (ICMR-INDIAB) - North East 2012-2015. [Data shared for this analysis]
Indian Council of Medical Research (ICMR), Madras Diabetes Research Foundation. Indian Council of Medical Research India Diabetes Study (ICMR-INDIAB) 2008-2010. [Data shared for this analysis]
Indian Council of Medical Research (ICMR), Madras Diabetes Research Foundation. Indian Council of Medical Research India Diabetes Study (ICMR-INDIAB) 2012-2013. [Data shared for this analysis]
Indian Council of Medical Research (ICMR). India Jai Vigyan Mission Mode Project: Community Control of Rheumatic Fever/Rheumatic Heart Disease in India Comprehensive Project Report 2001-2010. New Delhi, India: ICMR.
Indian Council of Medical Research (ICMR). India Study on Causes of Death by Verbal Autopsy 2003. New Delhi, India: ICMR. [Data shared for this analysis]
Indian Council of Medical Research, World Health Organization. India STEPS Noncommunicable Disease Risk Factors Survey 2003-2005. [Data shared for this analysis]
Institute of Health Systems, World Health Organization (WHO). WHO Multi-country Survey Study Report on Health and Health System Responsiveness, Andhra Pradesh 2000-2001.
International Institute for Population Sciences (IIPS), Ministry of Health and Family Welfare, Government of India, Harvard T. H. Chan School of Public Health, RAND Corporation, Monash University, University of California Los Angeles, Indian Academy of Geriatrics, National AIDS Research Institute, Columbia University. Longitudinal Aging Study in India, Pilot Data 2010. Mumbai, India: IIPS.
International Institute for Population Sciences (IIPS), Ministry of Health and Family Welfare, Government of India, Macro International. India National Family Health Survey Data (NFHS-1) 1992-1993. Mumbai, India: IIPS.
International Institute for Population Sciences (IIPS), Ministry of Health and Family Welfare, Government of India, Macro International. India National Family Health Survey (NFHS-1) 1992-1993: National Report. Mumbai, India: IIPS.
International Institute for Population Sciences (IIPS), Ministry of Health and Family Welfare, Government of India, ORC Macro. India National Family Health Survey Data (NFHS-2) 1998-1999. Mumbai, India: IIPS.
International Institute for Population Sciences (IIPS), Ministry of Health and Family Welfare, Government of India, ORC Macro. India National Family Health Survey (NFHS-2) 1998-1999: National and State Reports. Mumbai, India: IIPS.
International Institute for Population Sciences (IIPS), Ministry of Health and Family Welfare, Government of India, Macro International. India National Family Health Survey Data (NFHS-3) 2005-2006. Mumbai, India: IIPS.
International Institute for Population Sciences (IIPS), Ministry of Health and Family Welfare, Government of India, Macro International. India National Family Health Survey (NFHS-3) 2005-2006: National and State Reports. Mumbai, India: IIPS.
International Institute for Population Sciences (IIPS), Ministry of Health and Family Welfare, Government of India, ICF. India National Family Health Survey (NFHS-4) 2015-2016: National and State-level Factsheets. Mumbai, India: IIPS.

International Institute for Population Sciences (IIPS), Ministry of Health and Family Welfare, Government of India. India District Level Household Survey Data (DLHS-1) 1998-1999. Mumbai, India: IIPS.
International Institute for Population Sciences (IIPS), Ministry of Health and Family Welfare, Government of India. India District Level Household Survey (DLHS-1) 1998-1999: National Report. Mumbai, India: IIPS.
International Institute for Population Sciences (IIPS), Ministry of Health and Family Welfare, Government of India. India District Level Household Survey Data (DLHS-2) 2002-2004. Mumbai, India: IIPS.
International Institute for Population Sciences (IIPS), Ministry of Health and Family Welfare, Government of India. India District Level Household Survey (DLHS-2) 2002-2004: National and State Reports. Mumbai, India: IIPS.
International Institute for Population Sciences (IIPS), Ministry of Health and Family Welfare, Government of India. India District Level Household and Facility Survey Data (DLHS-3) 2007-2008. Mumbai, India: IIPS.
International Institute for Population Sciences (IIPS), Ministry of Health and Family Welfare, Government of India. India District Level Household and Facility Survey (DLHS-3) 2007-2008: National and State Reports. Mumbai, India: IIPS.
International Institute for Population Sciences (IIPS), Ministry of Health and Family Welfare, Government of India. India District Level Household and Facility Survey Data (DLHS-4) 2012-2013: State Reports. Mumbai, India: IIPS.
International Institute for Population Sciences, World Health Organization (WHO). India WHO Study on Global Ageing and Adult Health 2007-2008.
International Institute for Population Sciences, World Health Organization. India World Health Survey 2003.
Johnson C, Mohan S, Rogers K, Shivashankar R, Thout SR, Gupta P, He FJ, MacGregor GA, Webster J, Krishnan A, Maulik PK, Reddy KS, Prabhakaran D, Neal B. Mean Dietary Salt Intake in Urban and Rural Areas in India: A Population Survey of 1395 Persons. J Am Heart Assoc. 2017; 6(1):e004547.
Jose VJ, Gomathi M. Declining prevalence of rheumatic heart disease in rural schoolchildren in India: 2001-2002. Indian Heart J. 2003; 55(2): 158-60.
Joshi PP, Mohanan CJ, Sengupta SP, Salkar RG. Factors precipitating congestive heart failure – role of patient non-compliance. J Assoc Physicians India. 1999; 47(3): 294-5.
Joshi R, Cardona M, Iyengar S, Sukumar A, Raju CR, Raju KR, Raju K, Reddy KS, Lopez A, Neal B. Chronic diseases now a leading cause of death in rural India – mortality data from the Andhra Pradesh Rural Health Initiative. Int J Epidemiol. 2006; 35(6): 1522-9.
Kalkonde YV, Sahane V, Deshmukh MD, Nila S, Mandava P, Bang A. High Prevalence of Stroke in Rural Gadchiroli, India: A Community-Based Study. Neuroepidemiology. 2016; 46(4): 235-9.
Kamble P, Deshmukh PR, Garg N. Metabolic syndrome in adult population of rural Wardha, central India. Indian J Med Res. 2010; 132: 701–5.
Katyal R, Bansal R, Agrawal V, Goel K, Chaudhary V. Cross-sectional Study to Acknowledge the Independent Association of the Socio-demographic Determinants of Alcohol Use in an Urban Slum of North India. Int J Prev Med. 2014; 5(6): 749–57.
Kaushal SS, DasGupta DJ, Prashar BS, Bhardwaj AK. Electrocardiographic manifestations of healthy residents of a tribal Himalayan village. J Assoc Physicians India. 1995; 43(1): 15-6.
Kayina KP, Sharma AK, Agrawal K. Implementation of ICD 10: a study on the doctors' knowledge and coding practices in Delhi. Indian J Public Health. 2015; 59(1): 68–9.
KEM Hospital Research Center. India - Pimpale Cardiovascular Risk Factors Study Blood Pressure, Cholesterol, BMI, Blood Glucose, and Diabetes Incidence Measurements 1994-1999. [Data shared for this analysis]
Kim S, Rifkin S, John SM, Jacob KS. Nature, prevalence and risk factors of alcohol use in an urban slum of Southern India. Natl Med J India. 2013; 26(4): 203–9.
Koul R, Motta A, Razdan S. Epidemiology of young strokes in rural Kashmir, India. Acta Neurol Scand. 1990; 82(1): 1-3.
Krishnan MN, Zachariah G, Venugopal K, Mohanan PP, Harikrishnan S, Sanjay G, Jeyaseelan L, Thankappan KR. Prevalence of coronary artery disease and its risk factors in Kerala, South India: a community-based cross-sectional study. BMC Cardiovasc Disord. 2013; 16: 12.
Kulshrestha M, Vidyanand. An analysis of the risk factors and the outcomes of cerebrovascular diseases in northern India. J Clin Diagn Res. 2013; 7(1): 127–31.
Kumar P, Garhwal S, Chaudhary V. Rheumatic heart disease: a school survey in a rural area of Rajasthan. Indian Heart J. 1992; 44(4): 245-6.
Kumar P, Krishna P, Reddy SC, Gurappa M, Aravind SR, Munichoodappa C. Incidence of type 1 diabetes mellitus and associated complications among children and young adults: results from Karnataka Diabetes Registry 1995-2008. J Indian Med Assoc. 2008; 106(11): 708–11.
Kumar SG, Premarajan KC, Subitha L, Suguna E, Vinayagamoorthy, Kumar V. Prevalence and Pattern of Alcohol Consumption using Alcohol Use Disorders Identification Test (AUDIT) in Rural Tamil Nadu, India. J Clin Diagn Res. 2013; 7(8): 1637-9.

Kusuma Y, Das P. Hypertension in Orissa, India: a cross-sectional study among some tribal, rural and urban populations. Public Health. 2008; 122(10): 1120-3.

Kusuma Y, Gupta S, Pandav C. Migration and hypertension: a cross-sectional study among neo-migrants and settled-migrants in Delhi, India. Asia Pac J Public Health. 2009; 21(4): 497-507.

Kusuma YS, Babu BV, Naidu JM. Blood pressure levels among cross-cultural populations of Visakhapatnam district, Andhra Pradesh, India. Ann Hum Biol. 2002; 29(5): 502-12.

Madras Diabetes Research Foundation & M. V. Diabetes Specialities Centre. India - Chennai Urban Population Study Blood Glucose, Cholesterol, BMI, and Diabetes Incidence Measurements, 1996-2006. [Data shared for this analysis]

Madras Diabetes Research Foundation & M. V. Diabetes Specialities Centre. India - Chennai Urban Rural Epidemiology Study Blood Glucose, Cholesterol, BMI, and Diabetes Incidence Measurements, 2001-2013. [Data shared for this analysis]

Mahanta TG, Joshi R, Mahanta BN, Xavier D. Prevalence of modifiable cardiovascular risk factors among tea garden and general population in Dibrugarh, Assam, India. J Epidemiol Glob Health. 2013; 3(3): 147–56.

Malhotra P, Kumari S, Kumar R, Jain S, Sharma BK. Prevalence and determinants of hypertension in an un-industrialised rural population of North India. J Hum Hypertens. 1999; 13(7): 467-72.

Midha T, Idris MZ, Saran RK, Srivastav AK, Singh SK. Prevalence and determinants of hypertension in the urban and rural population of a north Indian district. East Afr J Public Health. 2009; 6(3): 268–73.

Ministry of Health and Family Welfare, Government of India, International Institute for Population Sciences, Centers for Disease Control and Prevention (CDC), Johns Hopkins Bloomberg School of Public Health, Research Triangle Institute, Inc., World Health Organization. India Global Adult Tobacco Survey 2009-2010. Atlanta, United States: CDC.

Ministry of Health and Family Welfare, Government of India, National Institute of Mental Health and Neurosciences, World Health Organization. India National Mental Health Survey Data Tables 2015-2016. [Data shared for this analysis]

Ministry of Health and Family Welfare, Government of India, World Health Organization - Regional Office for South-East Asia and Country Office for India, Centers for Disease Control and Prevention. India-Kerala Global Youth Tobacco Survey 2004. New Delhi, India: Ministry of Health and Family Welfare.

Ministry of Health and Family Welfare, Government of India, World Health Organization - Regional Office for South-East Asia and Country Office for India, Centers for Disease Control and Prevention. India-Tripura Global Youth Tobacco Survey 2000. New Delhi, India: Ministry of Health and Family Welfare.

Ministry of Health and Family Welfare, Government of India, World Health Organization - Regional Office for South-East Asia and Country Office for India, Centers for Disease Control and Prevention. India-Uttar Pradesh Global Youth Tobacco Survey 2002. New Delhi, India: Ministry of Health and Family Welfare.

Ministry of Health and Family Welfare, Government of India, World Health Organization - Regional Office for South-East Asia and Country Office for India, Centers for Disease Control and Prevention. India-Uttaranchal Global Youth Tobacco Survey 2002. New Delhi, India: Ministry of Health and Family Welfare.

Ministry of Health and Family Welfare, Government of India, World Health Organization - Regional Office for South-East Asia and Country Office for India, Centers for Disease Control and Prevention. India-West Bengal Global Youth Tobacco Survey 2000. New Delhi, India: Ministry of Health and Family Welfare.

Ministry of Health and Family Welfare, Government of India, World Health Organization - Regional Office for South-East Asia and Country Office for India, Centers for Disease Control and Prevention. India Global Youth Tobacco Survey 2006. New Delhi, India: Ministry of Health and Family Welfare.

Ministry of Health and Family Welfare, Government of India, World Health Organization - Regional Office for South-East Asia and Country Office for India, Centers for Disease Control and Prevention. India Global Youth Tobacco Survey 2009. New Delhi, India: Ministry of Health and Family Welfare.

Ministry of Health and Family Welfare, Government of India, World Health Organization - Regional Office for South-East Asia and Country Office for India, Centers for Disease Control and Prevention. India-Arunachal Pradesh Global Youth Tobacco Survey 2000. New Delhi, India: Ministry of Health and Family Welfare.

Ministry of Health and Family Welfare, Government of India, World Health Organization - Regional Office for South-East Asia and Country Office for India, Centers for Disease Control and Prevention. India-Assam Global Youth Tobacco Survey 2000. New Delhi, India: Ministry of Health and Family Welfare.

Ministry of Health and Family Welfare, Government of India, World Health Organization - Regional Office for South-East Asia and Country Office for India, Centers for Disease Control and Prevention. India-Bihar Global Youth Tobacco Survey 2000. New Delhi, India: Ministry of Health and Family Welfare.

Ministry of Health and Family Welfare, Government of India, World Health Organization - Regional Office for South-East Asia and Country Office for India, Centers for Disease Control and Prevention. India-Goa Global Youth Tobacco Survey 2000. New Delhi, India: Ministry of Health and Family Welfare.

Ministry of Health and Family Welfare, Government of India, World Health Organization - Regional Office for South-East Asia and Country Office for India, Centers for Disease Control and Prevention. India-Gujarat Global Youth Tobacco Survey 2004. New Delhi, India: Ministry of Health and Family Welfare.

Ministry of Health and Family Welfare, Government of India, World Health Organization - Regional Office for South-East Asia and Country Office for India, Centers for Disease Control and Prevention. India-Gujarat with Ahmedabad Global Youth Tobacco Survey 2004. New Delhi, India: Ministry of Health and Family Welfare.
Ministry of Health and Family Welfare, Government of India, World Health Organization - Regional Office for South-East Asia and Country Office for India, Centers for Disease Control and Prevention. India-Haryana Global Youth Tobacco Survey 2004. New Delhi, India: Ministry of Health and Family Welfare.
Ministry of Health and Family Welfare, Government of India, World Health Organization - Regional Office for South-East Asia and Country Office for India, Centers for Disease Control and Prevention. India-Jammu and Kashmir Global Youth Tobacco Survey 2004. New Delhi, India: Ministry of Health and Family Welfare.
Ministry of Health and Family Welfare, Government of India, World Health Organization - Regional Office for South-East Asia and Country Office for India, Centers for Disease Control and Prevention. India-Karnataka Global Youth Tobacco Survey 2003. New Delhi, India: Ministry of Health and Family Welfare.
Ministry of Health and Family Welfare, Government of India, World Health Organization - Regional Office for South-East Asia and Country Office for India, Centers for Disease Control and Prevention. India-Madhya Pradesh Global Youth Tobacco Survey 2004. New Delhi, India: Ministry of Health and Family Welfare.
Ministry of Health and Family Welfare, Government of India, World Health Organization - Regional Office for South-East Asia and Country Office for India, Centers for Disease Control and Prevention. India-Maharashtra Global Youth Tobacco Survey 2000. New Delhi, India: Ministry of Health and Family Welfare.
Ministry of Health and Family Welfare, Government of India, World Health Organization - Regional Office for South-East Asia and Country Office for India, Centers for Disease Control and Prevention. India-Manipur Global Youth Tobacco Survey 2000. New Delhi, India: Ministry of Health and Family Welfare.
Ministry of Health and Family Welfare, Government of India, World Health Organization - Regional Office for South-East Asia and Country Office for India, Centers for Disease Control and Prevention. India-Meghalaya Global Youth Tobacco Survey 2000. New Delhi, India: Ministry of Health and Family Welfare.
Ministry of Health and Family Welfare, Government of India, World Health Organization - Regional Office for South-East Asia and Country Office for India, Centers for Disease Control and Prevention. India-Mizoram Global Youth Tobacco Survey 2000. New Delhi, India: Ministry of Health and Family Welfare.
Ministry of Health and Family Welfare, Government of India, World Health Organization - Regional Office for South-East Asia and Country Office for India, Centers for Disease Control and Prevention. India-Nagaland Global Youth Tobacco Survey 2000. New Delhi, India: Ministry of Health and Family Welfare.
Ministry of Health and Family Welfare, Government of India, World Health Organization - Regional Office for South-East Asia and Country Office for India, Centers for Disease Control and Prevention. India-Punjab Global Youth Tobacco Survey 2004. New Delhi, India: Ministry of Health and Family Welfare.
Ministry of Health and Family Welfare, Government of India, World Health Organization - Regional Office for South-East Asia and Country Office for India, Centers for Disease Control and Prevention. India-Rajasthan Global Youth Tobacco Survey 2003. New Delhi, India: Ministry of Health and Family Welfare.
Ministry of Health and Family Welfare, Government of India, World Health Organization - Regional Office for South-East Asia and Country Office for India, Centers for Disease Control and Prevention. India-Sikkim Global Youth Tobacco Survey 2000. New Delhi, India: Ministry of Health and Family Welfare.
Ministry of Health and Family Welfare, Government of India, World Health Organization - Regional Office for South-East Asia and Country Office for India, Centers for Disease Control and Prevention. India-Tamil Nadu Global Youth Tobacco Survey 2000. New Delhi, India: Ministry of Health and Family Welfare.
Ministry of Social Justice and Empowerment, Government of India, United Nations Office on Drugs and Crime. Extent, Pattern and Trends of Drug Abuse in India. New Delhi, India: Ministry of Social Justice and Empowerment; 2004.
Ministry of Statistics and Programme Implementation, Government of India. Consumer Expenditure Survey, National Sample Survey Round 38, January-December 1983. New Delhi, India: Ministry of Statistics and Programme Implementation.
Ministry of Statistics and Programme Implementation, Government of India. Employment and Unemployment Survey, National Sample Survey Round 38, January-December 1983. New Delhi, India: Ministry of Statistics and Programme Implementation.
Ministry of Statistics and Programme Implementation, Government of India. Survey on Participation in Education, National Sample Survey Round 42, July 1986- June 1987. New Delhi, India: Ministry of Statistics and Programme Implementation.
Ministry of Statistics and Programme Implementation, Government of India. Consumer Expenditure Survey, National Sample Survey Round 43, July 1987-June 1988. New Delhi, India: Ministry of Statistics and Programme Implementation.
Ministry of Statistics and Programme Implementation, Government of India. Employment and Unemployment Survey, National Sample Survey Round 43, July 1987- June 1988. New Delhi, India: Ministry of Statistics and Programme Implementation.
Ministry of Statistics and Programme Implementation, Government of India. Consumer Expenditure Survey, National Sample Survey Round 47, July - December 1991. New Delhi, India: Ministry of Statistics and Programme Implementation.
Ministry of Statistics and Programme Implementation, Government of India. Survey on Literacy and Culture, National Sample Survey Round 47, July - December 1991. New Delhi, India: Ministry of Statistics and Programme Implementation.

Ministry of Statistics and Programme Implementation, Government of India. Consumer Expenditure Survey, National Sample Survey Round 49, January-June 1993. New Delhi, India: Ministry of Statistics and Programme Implementation.

Ministry of Statistics and Programme Implementation, Government of India. Consumer Expenditure Survey, National Sample Survey Round 50, July 1993-June 1994. New Delhi, India: Ministry of Statistics and Programme Implementation.

Ministry of Statistics and Programme Implementation, Government of India. Employment and Unemployment Survey, National Sample Survey Round 50, July 1993- June 1994. New Delhi, India: Ministry of Statistics and Programme Implementation.

Ministry of Statistics and Programme Implementation, Government of India. Consumer Expenditure Survey, National Sample Survey Round 52, July 1995-June 1996. New Delhi, India: Ministry of Statistics and Programme Implementation.

Ministry of Statistics and Programme Implementation, Government of India. Participation in Education Survey, National Sample Survey Round 52, July 1995-June 1996. New Delhi, India: Ministry of Statistics and Programme Implementation.

Ministry of Statistics and Programme Implementation, Government of India. Survey on Healthcare, National Sample Survey Round 52, July 1995-June 1996. New Delhi, India: Ministry of Statistics and Programme Implementation.

Ministry of Statistics and Programme Implementation, Government of India. Common Property Resources, Sanitation & Hygiene Services, National Sample Survey Round 54, January - June 1998. New Delhi, India: Ministry of Statistics and Programme Implementation.

Ministry of Statistics and Programme Implementation, Government of India. Consumer Expenditure Survey, National Sample Survey Round 54, January - June 1998. New Delhi, India: Ministry of Statistics and Programme Implementation.

Ministry of Statistics and Programme Implementation, Government of India. Consumer Expenditure Survey, India National Sample Survey Round 55, July 1999-June 2000. New Delhi, India: Ministry of Statistics and Programme Implementation.

Ministry of Statistics and Programme Implementation, Government of India. Employment and Unemployment Survey, India National Sample Survey Round 55, July 1999-June 2000. New Delhi, India: Ministry of Statistics and Programme Implementation.

Ministry of Statistics and Programme Implementation, Government of India. Consumer Expenditure Survey, National Sample Survey Round 56, July 2000 - June 2001. New Delhi, India: Ministry of Statistics and Programme Implementation.

Ministry of Statistics and Programme Implementation, Government of India. Consumer Expenditure Survey, National Sample Survey Round 60, January-June 2004. New Delhi, India: Ministry of Statistics and Programme Implementation.

Ministry of Statistics and Programme Implementation, Government of India. Employment and Unemployment surveys, National Sample Survey Round 60, January-June 2004. New Delhi, India: Ministry of Statistics and Programme Implementation.

Ministry of Statistics and Programme Implementation, Government of India. Survey on Morbidity and Healthcare, National Sample Survey Round 60, January-June 2004. New Delhi, India: Ministry of Statistics and Programme Implementation.

Ministry of Statistics and Programme Implementation, Government of India. Consumer Expenditure Survey, National Sample Survey Round 61, July 2004 - June 2005. New Delhi, India: Ministry of Statistics and Programme Implementation.

Ministry of Statistics and Programme Implementation, Government of India. Employment and Unemployment Survey, National Sample Survey Round 61, July 2004- June 2005. New Delhi, India: Ministry of Statistics and Programme Implementation.

Ministry of Statistics and Programme Implementation, Government of India. Consumer Expenditure Survey, National Sample Survey Round 63, July 2006- June 2007. New Delhi, India: Ministry of Statistics and Programme Implementation.

Ministry of Statistics and Programme Implementation, Government of India. Consumer Expenditure Survey, National Sample Survey Round 64, July 2007- June 2008. New Delhi, India: Ministry of Statistics and Programme Implementation.

Ministry of Statistics and Programme Implementation, Government of India. Employment and Unemployment Survey, National Sample Survey Round 64, July 2007- June 2008. New Delhi, India: Ministry of Statistics and Programme Implementation.

Ministry of Statistics and Programme Implementation, Government of India. Participation & Expenditure in Education Survey, National Sample Survey Round 64, July 2007- June 2008. New Delhi, India: Ministry of Statistics and Programme Implementation.

Ministry of Statistics and Programme Implementation, Government of India. Social Consumption Survey on Health and Education, National Sample Survey Round 71, January 2014 - June 2014. New Delhi, India: Ministry of Statistics and Programme Implementation.

Ministry of Statistics and Programme Implementation, Government of India. Household expenditure on services and durable goods survey, National Sample Survey Round 72, July 2014 - June 2015. New Delhi, India: Ministry of Statistics and Programme Implementation.

Ministry of Statistics and Programme Implementation, Government of India. India State Series: Gross State Domestic Product at Factor Cost by Industry of Origin Tables 1980-81 to 1993-94. New Delhi, India: Ministry of Statistics and Programme Implementation.

Ministry of Statistics and Programme Implementation, Government of India. India State Series: Gross State Domestic Product at Factor Cost by Industry of Origin Tables 1993-94 to 2004-05. New Delhi, India: Ministry of Statistics and Programme Implementation.
Ministry of Statistics and Programme Implementation, Government of India. India State Series: Gross State Domestic Product at Factor Cost by Industry of Origin Tables 1999-2000 to 2009-10. New Delhi, India: Ministry of Statistics and Programme Implementation.
Ministry of Statistics and Programme Implementation, Government of India. India State Series: Gross State Domestic Product at Factor Cost by Industry of Origin Tables 2004-05 to 2013-14. New Delhi, India: Ministry of Statistics and Programme Implementation.
Mir MA, Mir F, Khosla T, Newcombe R. The relationship of salt intake and arterial blood pressure in salted-tea drinking Kashmiris. Int J Cardiol. 1986; 13(3): 279-88.
Misra M, Mittal M, Singh R, Verma A, Rai R, Chandra G, Singh D, Chauhan R, Chowdhary V, Singh R, Mall A, Khan MJ, Khare S, Yadav K. Prevalence of rheumatic heart disease in school-going children of Eastern Uttar Pradesh. Indian Heart J. 2007; 59(1): 42-3.
Mohan V, Deepa M, Anjana RM, Lanthorn H, Deepa R. Incidence of diabetes and pre-diabetes in a selected urban south Indian population (CUPS - 19). J Assoc Physicians India. 2008; 56: 152-7.
Mohan V, Deepa M, Farooq S, Datta M, Deepa R. Prevalence, awareness and control of hypertension in Chennai--The Chennai Urban Rural Epidemiology Study (CURES-52). J Assoc Physicians India. 2007; 55: 326-32.
Mohan V, Mathur P, Deepa R, Deepa M, Shukla DK, Menon GR, Anand K, Desai NG, Joshi PP, Mahanta J, Thankappan KR, Shah B. Urban rural differences in prevalence of self-reported diabetes in India--the WHO-ICMR Indian NCD risk factor surveillance. Diabetes Res Clin Pract. 2008; 80(1): 159-68.
Mohanan PP, Mathew R, Harikrishnan S, Krishnan MN, Zachariah G, Joseph J, Eapen K, Abraham M, Menon J, Thomas M, Jacob S, Huffman MD, Prabhakaran D, Kerala ACS Registry Investigators. Presentation, management, and outcomes of 25 748 acute coronary syndrome admissions in Kerala, India: results from the Kerala ACS Registry. Eur Heart J. 2013; 34(2): 121–9.
Nadkarni A, Weiss HA, Naik A, Bhat B, Patel V. The six-year outcome of alcohol use disorders in men: A population based study from India. Drug Alcohol Depend. 2016; 162: 107–15.
National Institute of Medical Statistics, Indian Council of Medical Research (ICMR), Integrated Disease Surveillance Programme. Non-Communicable Disease Risk Factors Survey Data 2007-2008. New Delhi, India: ICMR. [Data shared for this analysis]
National Institute of Medical Statistics, Indian Council of Medical Research (ICMR), Integrated Disease Surveillance Programme. Non-Communicable Disease Risk Factors Survey Report 2007-2008. New Delhi, India: ICMR.
National Nutrition Monitoring Bureau, National Institute of Nutrition (NIN), Indian Council of Medical Research. India Rural First Repeat Survey of Diet and Nutritional Status Data 1988-1990. [Data shared for this analysis]
National Nutrition Monitoring Bureau, National Institute of Nutrition (NIN), Indian Council of Medical Research. India Rural First Repeat Survey of Diet and Nutritional Status Report 1988-1990. Hyderabad, India: NIN.
National Nutrition Monitoring Bureau, National Institute of Nutrition (NIN), Indian Council of Medical Research. India Rural Survey of Diet and Nutritional Status Data 1994-1995. [Data shared for this analysis]
National Nutrition Monitoring Bureau, National Institute of Nutrition (NIN), Indian Council of Medical Research. India Rural Survey of Diet and Nutritional Status Report 1994-1995. Hyderabad, India: NIN.
National Nutrition Monitoring Bureau, National Institute of Nutrition (NIN), Indian Council of Medical Research. India Rural Second Repeat Survey of Diet and Nutritional Status Data 1996-1997. [Data shared for this analysis]
National Nutrition Monitoring Bureau, National Institute of Nutrition (NIN), Indian Council of Medical Research. India Rural Second Repeat Survey of Diet and Nutritional Status Report 1996-1997. Hyderabad, India: NIN.
National Nutrition Monitoring Bureau, National Institute of Nutrition (NIN), Indian Council of Medical Research. India Rural Survey of Diet and Nutritional Status Data 2000-2001. [Data shared for this analysis]
National Nutrition Monitoring Bureau, National Institute of Nutrition (NIN), Indian Council of Medical Research. India Rural Survey of Diet and Nutritional Status Report 2000-2001. Hyderabad, India: NIN.
National Nutrition Monitoring Bureau, National Institute of Nutrition (NIN), Indian Council of Medical Research. India Rural Survey of Diet and Nutritional Status Data 2004-2006. [Data shared for this analysis]
National Nutrition Monitoring Bureau, National Institute of Nutrition (NIN), Indian Council of Medical Research. India Rural Survey of Diet and Nutritional Status Report 2004-2006. Hyderabad, India: NIN.
National Nutrition Monitoring Bureau, National Institute of Nutrition (NIN), Indian Council of Medical Research. India Rural Third Repeat Survey of Diet and Nutritional Status Data 2011-2012. [Data shared for this analysis]
National Nutrition Monitoring Bureau, National Institute of Nutrition (NIN), Indian Council of Medical Research. India Rural Third Repeat Survey of Diet and Nutritional Status Report 2011-2012. Hyderabad, India: NIN.
National Nutrition Monitoring Bureau, National Institute of Nutrition (NIN), Indian Council of Medical Research. India Survey of Diet and Nutritional Status Data 1990-1992. [Data shared for this analysis]

National Nutrition Monitoring Bureau, National Institute of Nutrition (NIN), Indian Council of Medical Research. India Survey of Diet and Nutritional Status Report 1990-1992. Hyderabad, India: NIN.
National Nutrition Monitoring Bureau, National Institute of Nutrition (NIN), Indian Council of Medical Research. India Tribal First Repeat Survey of Diet and Nutritional Status Data 1998-1999. [Data shared for this analysis]
National Nutrition Monitoring Bureau, National Institute of Nutrition (NIN), Indian Council of Medical Research. India Tribal First Repeat Survey of Diet and Nutritional Status Report 1998-1999. Hyderabad, India: NIN.
National Nutrition Monitoring Bureau, National Institute of Nutrition (NIN), Indian Council of Medical Research. India Tribal Second Repeat Survey of Diet and Nutritional Status Data 2007-2008. [Data shared for this analysis]
National Nutrition Monitoring Bureau, National Institute of Nutrition (NIN), Indian Council of Medical Research. India Tribal Second Repeat Survey of Diet and Nutritional Status Report 2007-2008. Hyderabad, India: NIN.
National Nutrition Monitoring Bureau, National Institute of Nutrition (NIN), Indian Council of Medical Research. India Urban Slums Survey of Diet and Nutritional Status Data 1993-1994. [Data shared for this analysis]
National Nutrition Monitoring Bureau, National Institute of Nutrition (NIN), Indian Council of Medical Research. India Urban Slums Survey of Diet and Nutritional Status Report 1993-1994. Hyderabad, India: NIN.
National Nutrition Monitoring Bureau, National Institute of Nutrition, Indian Council of Medical Research. India National Nutrition Monitoring Bureau Eight States Pooled Data 1991-1992. [Data shared for this analysis]
Nazareth Hospital. Inpatient and Discharge Data 2014. Shillong, Meghalaya: Nazareth Hospital. [Data shared for this analysis]
Nazir A, Papita R, Anbalagan VP, Anjana RM, Deepa M, Mohan V. Prevalence of diabetes in Asian Indians based on glycated hemoglobin and fasting and 2-H post-load (75-g) plasma glucose (CURES-120). Diabetes Technol Ther. 2012; 14(8): 665–8.
Negi PC, Kanwar A, Chauhan R, Asotra S, Thakur JS, Bhardwaj AK. Epidemiological trends of RF/RHD in school children of Shimla in north India. Indian J Med Res. 2013; 137(6.0): 1121-7.
Norboo T, Stobdan T, Tsering N, Angchuk N, Tsering P, Ahmed I, Chorol T, Kumar Sharma V, Reddy P, Singh SB, Kimura Y, Sakamoto R, Fukutomi E, Ishikawa M, Suwa K, Kosaka Y, Nose M, Yamaguchi T, Tsukihara T, Matsubayashi K, Otsuka K, Okumiya K. Prevalence of hypertension at high altitude: cross-sectional survey in Ladakh, Northern India 2007-2011. BMJ Open. 2015; 5(4): e007026.
Office of the Registrar General & Census Commissioner, Centre for Global Health Research - University of Toronto. India SRS Special Fertility and Mortality Survey 1998. New Delhi, India: Office of the Registrar General & Census Commissioner; 2005.
Office of the Registrar General & Census Commissioner, Ministry of Home Affairs, Government of India. India Annual Health Survey Data 2010-2011. New Delhi, India: Office of the Registrar General & Census Commissioner.
Office of the Registrar General & Census Commissioner, Ministry of Home Affairs, Government of India. India Annual Health Survey Report 2010-2011. New Delhi, India: Office of the Registrar General & Census Commissioner.
Office of the Registrar General & Census Commissioner, Ministry of Home Affairs, Government of India. India Annual Health Survey Data 2011-2012. New Delhi, India: Office of the Registrar General & Census Commissioner.
Office of the Registrar General & Census Commissioner, Ministry of Home Affairs, Government of India. India Annual Health Survey Report 2011-2012. New Delhi, India: Office of the Registrar General & Census Commissioner.
Office of the Registrar General & Census Commissioner, Ministry of Home Affairs, Government of India. India Annual Health Survey Data 2012-2013. New Delhi, India: Office of the Registrar General & Census Commissioner.
Office of the Registrar General & Census Commissioner, Ministry of Home Affairs, Government of India. India Annual Health Survey Report 2012-2013. New Delhi, India: Office of the Registrar General & Census Commissioner.
Office of the Registrar General & Census Commissioner, Ministry of Home Affairs, Government of India. India Annual Health Survey Data 2010-2013. New Delhi, India: Office of the Registrar General & Census Commissioner.
Office of the Registrar General & Census Commissioner, Ministry of Home Affairs, Government of India. India Census 1981. New Delhi, India: Office of the Registrar General & Census Commissioner.
Office of the Registrar General & Census Commissioner, Ministry of Home Affairs, Government of India. India Medical Certification of Cause of Death Report 1997. New Delhi, India: Office of the Registrar General & Census Commissioner.
Office of the Registrar General & Census Commissioner, Ministry of Home Affairs, Government of India. India Medical Certification of Cause of Death Report 2005. New Delhi, India: Office of the Registrar General & Census Commissioner.
Office of the Registrar General & Census Commissioner, Ministry of Home Affairs, Government of India. India Medical Certification of Cause of Death Report 2006. New Delhi, India: Office of the Registrar General & Census Commissioner.
Office of the Registrar General & Census Commissioner, Ministry of Home Affairs, Government of India. India Medical Certification of Cause of Death Report 2008. New Delhi, India: Office of the Registrar General & Census Commissioner.

Office of the Registrar General & Census Commissioner, Ministry of Home Affairs, Government of India. India Medical Certification of Cause of Death Report 2009. New Delhi, India: Office of the Registrar General & Census Commissioner; 2014.
Office of the Registrar General & Census Commissioner, Ministry of Home Affairs, Government of India. India Medical Certification of Cause of Death Report 2010. New Delhi, India: Office of the Registrar General & Census Commissioner; 2014.
Office of the Registrar General & Census Commissioner, Ministry of Home Affairs, Government of India. India Medical Certification of Cause of Death State-Level Tabulations 1990. New Delhi, India: Office of the Registrar General & Census Commissioner.
Office of the Registrar General & Census Commissioner, Ministry of Home Affairs, Government of India. India Medical Certification of Cause of Death State-Level Tabulations 1991. New Delhi, India: Office of the Registrar General & Census Commissioner.
Office of the Registrar General & Census Commissioner, Ministry of Home Affairs, Government of India. India Medical Certification of Cause of Death State-Level Tabulations 1992. New Delhi, India: Office of the Registrar General & Census Commissioner.
Office of the Registrar General & Census Commissioner, Ministry of Home Affairs, Government of India. India Medical Certification of Cause of Death State-Level Tabulations 1993. New Delhi, India: Office of the Registrar General & Census Commissioner.
Office of the Registrar General & Census Commissioner, Ministry of Home Affairs, Government of India. India Medical Certification of Cause of Death State-Level Tabulations 1994. New Delhi, India: Office of the Registrar General & Census Commissioner.
Office of the Registrar General & Census Commissioner, Ministry of Home Affairs, Government of India. India Medical Certification of Cause of Death State-Level Tabulations 1995. New Delhi, India: Office of the Registrar General & Census Commissioner.
Office of the Registrar General & Census Commissioner, Ministry of Home Affairs, Government of India. India Medical Certification of Cause of Death State-Level Tabulations 1996. New Delhi, India: Office of the Registrar General & Census Commissioner.
Office of the Registrar General & Census Commissioner, Ministry of Home Affairs, Government of India. India Medical Certification of Cause of Death State-Level Tabulations 1998. New Delhi, India: Office of the Registrar General & Census Commissioner.
Office of the Registrar General & Census Commissioner, Ministry of Home Affairs, Government of India. India Medical Certification of Cause of Death State-Level Tabulations 1999. New Delhi, India: Office of the Registrar General & Census Commissioner.
Office of the Registrar General & Census Commissioner, Ministry of Home Affairs, Government of India. India Medical Certification of Cause of Death State-Level Tabulations 2000. New Delhi, India: Office of the Registrar General & Census Commissioner.
Office of the Registrar General & Census Commissioner, Ministry of Home Affairs, Government of India. India Medical Certification of Cause of Death State-Level Tabulations 2001. New Delhi, India: Office of the Registrar General & Census Commissioner.
Office of the Registrar General & Census Commissioner, Ministry of Home Affairs, Government of India. India Medical Certification of Cause of Death State-Level Tabulations 2002. New Delhi, India: Office of the Registrar General & Census Commissioner.
Office of the Registrar General & Census Commissioner, Ministry of Home Affairs, Government of India. India Medical Certification of Cause of Death State-Level Tabulations 2003. New Delhi, India: Office of the Registrar General & Census Commissioner.
Office of the Registrar General & Census Commissioner, Ministry of Home Affairs, Government of India. India Medical Certification of Cause of Death State-Level Tabulations 2004. New Delhi, India: Office of the Registrar General & Census Commissioner.
Office of the Registrar General & Census Commissioner, Ministry of Home Affairs, Government of India. India Population and Housing Census 1991. New Delhi, India: Office of the Registrar General & Census Commissioner.
Office of the Registrar General & Census Commissioner, Ministry of Home Affairs, Government of India. India Population and Housing Census 2001. New Delhi, India: Office of the Registrar General & Census Commissioner.
Office of the Registrar General & Census Commissioner, Ministry of Home Affairs, Government of India. India Population and Housing Census 2011. New Delhi, India: Office of the Registrar General & Census Commissioner; 2012.
Office of the Registrar General & Census Commissioner, Ministry of Home Affairs, Government of India. India Sample Registration System Baseline Survey 2014. New Delhi, India: Office of the Registrar General & Census Commissioner.
Office of the Registrar General & Census Commissioner, Ministry of Home Affairs, Government of India. India SRS Statistical Report 1999. New Delhi, India: Office of the Registrar General & Census Commissioner.

Office of the Registrar General & Census Commissioner, Ministry of Home Affairs, Government of India. India SRS Statistical Report 2000. New Delhi, India: Office of the Registrar General & Census Commissioner.
Office of the Registrar General & Census Commissioner, Ministry of Home Affairs, Government of India. India SRS Statistical Report 2001. New Delhi, India: Office of the Registrar General & Census Commissioner; 2005.
Office of the Registrar General & Census Commissioner, Ministry of Home Affairs, Government of India. India SRS Statistical Report 2002. New Delhi, India: Office of the Registrar General & Census Commissioner; 2005.
Office of the Registrar General & Census Commissioner, Ministry of Home Affairs, Government of India. India SRS Statistical Report 2003. New Delhi, India: Office of the Registrar General and Census Commissioner; 2005.
Office of the Registrar General & Census Commissioner, Ministry of Home Affairs, Government of India. India SRS Statistical Report 2004. New Delhi, India: Office of the Registrar General and Census Commissioner; 2006.
Office of the Registrar General & Census Commissioner, Ministry of Home Affairs, Government of India. India SRS Statistical Report 2005. New Delhi, India: Office of the Registrar General and Census Commissioner; 2006.
Office of the Registrar General & Census Commissioner, Ministry of Home Affairs, Government of India. India SRS Statistical Report 2006. New Delhi, India: Office of the Registrar General and Census Commissioner; 2008.
Office of the Registrar General & Census Commissioner, Ministry of Home Affairs, Government of India. India SRS Statistical Report 2007. New Delhi, India: Office of the Registrar General and Census Commissioner; 2008.
Office of the Registrar General & Census Commissioner, Ministry of Home Affairs, Government of India. India SRS Statistical Report 2008. New Delhi, India: Office of the Registrar General and Census Commissioner; 2010.
Office of the Registrar General & Census Commissioner, Ministry of Home Affairs, Government of India. India SRS Statistical Report 2009. New Delhi, India: Office of the Registrar General and Census Commissioner.
Office of the Registrar General & Census Commissioner, Ministry of Home Affairs, Government of India. India SRS Statistical Report 2010. New Delhi, India: Office of the Registrar General and Census Commissioner; 2012.
Office of the Registrar General & Census Commissioner, Ministry of Home Affairs, Government of India. India SRS Statistical Report 2011. New Delhi, India: Office of the Registrar General and Census Commissioner; 2013.
Office of the Registrar General & Census Commissioner, Ministry of Home Affairs, Government of India. India SRS Statistical Report 2012. New Delhi, India: Office of the Registrar General and Census Commissioner; 2013.
Office of the Registrar General & Census Commissioner, Ministry of Home Affairs, Government of India. India SRS Statistical Report 2013. New Delhi, India: Office of the Registrar General and Census Commissioner; 2014.
Office of the Registrar General & Census Commissioner, Ministry of Home Affairs, Government of India. India SRS Statistical Report 2014. New Delhi, India: Office of the Registrar General and Census Commissioner.
Office of the Registrar General & Census Commissioner, Ministry of Home Affairs, Government of India. India SRS Verbal Autopsy 2004-2006. New Delhi, India: Office of the Registrar General and Census Commissioner. [Data shared for this analysis]
Office of the Registrar General & Census Commissioner, Ministry of Home Affairs, Government of India. India SRS Verbal Autopsy Report 2004-2006. New Delhi, India: Office of the Registrar General and Census Commissioner.
Office of the Registrar General & Census Commissioner, Ministry of Home Affairs, Government of India. India SRS Verbal Autopsy 2007-2009. New Delhi, India: Office of the Registrar General and Census Commissioner. [Data shared for this analysis]
Office of the Registrar General & Census Commissioner, Ministry of Home Affairs, Government of India. India SRS Verbal Autopsy Report 2007-2009. New Delhi, India: Office of the Registrar General and Census Commissioner.
Office of the Registrar General & Census Commissioner, Ministry of Home Affairs, Government of India. India SRS Verbal Autopsy 2010-2013. New Delhi, India: Office of the Registrar General and Census Commissioner. [Data shared for this analysis]
Office of the Registrar General & Census Commissioner, Ministry of Home Affairs, Government of India. India SRS Verbal Autopsy Report 2010-2013. New Delhi, India: Office of the Registrar General and Census Commissioner.
Office of the Registrar General & Census Commissioner, Ministry of Home Affairs, Government of India. India Vital Statistics 1980. New Delhi, India: Office of the Registrar General and Census Commissioner.
Office of the Registrar General & Census Commissioner, Ministry of Home Affairs, Government of India. India Vital Statistics 1981. New Delhi, India: Office of the Registrar General and Census Commissioner.
Office of the Registrar General & Census Commissioner, Ministry of Home Affairs, Government of India. India Vital Statistics 1982. New Delhi, India: Office of the Registrar General and Census Commissioner.
Office of the Registrar General & Census Commissioner, Ministry of Home Affairs, Government of India. India Vital Statistics 1983. New Delhi, India: Office of the Registrar General and Census Commissioner.
Office of the Registrar General & Census Commissioner, Ministry of Home Affairs, Government of India. India Vital Statistics 1984. New Delhi, India: Office of the Registrar General and Census Commissioner.
Office of the Registrar General & Census Commissioner, Ministry of Home Affairs, Government of India. India Vital Statistics 1986. New Delhi, India: Office of the Registrar General and Census Commissioner.
Office of the Registrar General & Census Commissioner, Ministry of Home Affairs, Government of India. India Vital Statistics 1987. New Delhi, India: Office of the Registrar General and Census Commissioner.
Office of the Registrar General & Census Commissioner, Ministry of Home Affairs, Government of India. India Vital Statistics 1989. New Delhi, India: Office of the Registrar General and Census Commissioner.

Office of the Registrar General and Census Commissioner, Ministry of Health and Family Welfare, Government of India, National Institute of Health and Family Welfare, Nutrition Foundation of India, National Institute of Nutrition, Indian Council of Medical Research. India Clinical, Anthropometric and Bio-chemical (CAB) Survey Data 2014 [Biomarker Component of Annual Health Survey]. New Delhi, India: Office of the Registrar General and Census Commissioner.

Oommen AM, Abraham VJ, George K, Jose VJ. Prevalence of coronary heart disease in rural and urban Vellore: A repeat cross-sectional survey. Indian Heart J. 2016; 68(4): 473-9.

Pagidipati NJ, Huffman MD, Jeemon P, Gupta R, Negi P, Jaison TM, Sharma S, Sinha N, Mohanan P, Muralidhara BG, Bijulal S, Sivasankaran S, Puri VK, Jose J, Reddy KS, Prabhakaran D. Association between gender, process of care measures, and outcomes in ACS in India: results from the detection and management of coronary heart disease (DEMAT) registry. PLoS One. 2013; 8(4): e62061.

Pandian JD, Singh G, Kaur P, Bansal R, Paul BS, Singla M, Singh S, Samuel CJ, Verma SJ, Moodbidri P, Mehmi G, Sharma A, Arora OP, Dhanuka AK, Sobti MK, Sehgal H, Kaur M, Grewal SS, Jhawar SS, Shadangi TN, Arora T, Saxena A, Sachdeva G, Gill JS, Brar RS, Gill A, Bakshi SS, Pawar SS, Singh G, Sikka P, Litoria PK, Sharma M. Incidence, short-term outcome, and spatial distribution of stroke patients in Ludhiana, India. Neurology. 2016; 86(5): 425-33.

Pandian JD. Ludhiana Population Based Stroke Registry 2012-2013 [Data shared for this analysis]

Pazhanivel M, Jayanthi V. Diabetes mellitus and cirrhosis liver. Minerva Gastroenterol Dietol. 2010; 56(1): 7-11.

Pemminati S, Prabha Adhikari MR, Pathak R, Pai MRSM. Prevalence of metabolic syndrome (METS) using IDF 2005 guidelines in a semi urban south Indian (Boloor Diabetes Study) population of Mangalore. J Assoc Physicians India. 2010; 58: 674–7.

Periwal KL, Gupta BK, Panwar RB, Khatri PC, Raja S, Gupta R. Prevalence of rheumatic heart disease in school children in Bikaner: an echocardiographic study. J Assoc Physicians India. 2006; 54: 279-82.

Pillai A, Nayak MB, Greenfield TK, Bond JC, Hasin DS, Patel V. Adolescent drinking onset and its adult consequences among men: A population based study from India. J Epidemiol Community Health. 2014; 68(10): 922–7.

Pillai A, Nayak MB, Greenfield TK, Bond JC, Nadkarni A, Patel V. Patterns of alcohol use, their correlates, and impact in male drinkers: a population-based survey from Goa, India. Soc Psychiatry Psychiatr Epidemiol. 2013; 48(2): 275–82.

Prasad DS, Kabir Z, Dash AK, Das BC. Prevalence and risk factors for diabetes and impaired glucose tolerance in Asian Indians: a community survey from urban eastern India. Diabetes Metab Syndr. 2012; 6(2): 96-101.

Public Health Foundation of India. India Cause of Death Estimation Study in Bihar 2011-2014. New Delhi, India: Public Health Foundation of India. [Data shared for this analysis]

Radhakrishnan S, Balamurugan S. Prevalence of diabetes and hypertension among geriatric population in a rural community of Tamilnadu. Indian J Med Sci. 2013; 67(5-6): 130-8.

Rajput R, Rajput M, Singh J, Bairwa M. Prevalence of diabetes mellitus among the adult population in rural blocks of Haryana, India: a community-based study. Metab Syndr Relat Disord. 2012; 10(6): 443-6.

Rama Kumari N, Bhaskara Raju I, Patnaik AN, Barik R, Singh A, Pushpanjali A, Laxmi V, Satya Ramakrishna L. Prevalence of rheumatic and congenital heart disease in school children of Andhra Pradesh, South India. J Cardiovasc Dis Res. 2013; 4(1): 11-4.

Ramachandran A, Mary S, Yamuna A, Murugesan N, Snehalatha C. High prevalence of diabetes and cardiovascular risk factors associated with urbanization in India. Diabetes Care. 2008; 31(5): 893-8.

Ramachandran A, Snehalatha C, Abdul Khader OM, Joseph TA, Viswanathan M. Prevalence of childhood diabetes in an urban population in south India. Diabetes Res Clin Pract. 1992; 17(3): 227-31.

Ramachandran A, Snehalatha C, Kapur A, Vijay V, Mohan V, Das AK, Rao PV, Yajnik CS, Prasanna Kumar KM, Nair JD. High prevalence of diabetes and impaired glucose tolerance in India: National Urban Diabetes Survey. Diabetologia. 2001; 44(9): 1094-101.

Raman Kutty V, Joseph A, Soman CR. High prevalence of type 2 diabetes in an urban settlement in Kerala, India. Ethn Health. 1999; 4(4): 231-9.

Rao CR, Kamath VG, Shetty A, Kamath A. A study on the prevalence of type 2 diabetes in coastal Karnataka. Int J Diabetes Dev Ctries. 2010; 30(2): 80-5.

Rathod SD, Nadkarni A, Bhana A, Shidhaye R. Epidemiological features of alcohol use in rural India: a population-based cross-sectional study. BMJ Open. 2015; 5(12): e009802.

Ravikumar P, Bhansali A, Ravikiran M, Bhansali S, Walia R, Shanmugasundar G, Thakur JS, Kumar Bhadada S, Dutta P. Prevalence and risk factors of diabetes in a community-based study in North India: the Chandigarh Urban Diabetes Study (CUDS). Diabetes Metab. 2011; 37(3): 216-21.

Razdan S, Koul RL, Motta A, Kaul S. Cerebrovascular disease in rural Kashmir, India. Stroke. 1989; 20(12): 1691–3.

Reddy KK, Rao AP, Reddy TP. Socioeconomic status and the prevalence of coronary heart disease risk factors. Asia Pac J Clin Nutr. 2002; 11(2): 98-103.

Ritchie GE, Kengne AP, Joshi R, Chow C, Neal B, Patel A, Zoungas S. Comparison of near-patient capillary glucose measurement and a risk assessment questionnaire in screening for type 2 diabetes in a high-risk population in rural India. Diabetes Care. 2011; 34(1): 44–9.

Sachdev HPS, Osmond C, Fall CHD, Lakshmy R, Ramji S, Dey Biswas SK, Prabhakaran D, Tandon N, Reddy KS, Barker DJP, Bhargava SK. Predicting adult metabolic syndrome from childhood body mass index: follow-up of the New Delhi birth cohort. Arch Dis Child. 2009; 94(10): 768-74.

Sadikot SM, Nigam A, Das S, Bajaj S, Zargar AH, Prasannakumar KM, Sosale A, Munichoodappa C, Seshiah V, Singh SK, Jamal A, Sai K, Sadasivrao Y, Murthy SS, Hazra DK, Jain S, Mukherjee S, Bandyopadhay S, Sinha NK, Mishra R, Dora M, Jena B, Patra P, Goenka K. The burden of diabetes and impaired glucose tolerance in India using the WHO 1999 criteria: prevalence of diabetes in India study (PODIS). Diabetes Res Clin Pract. 2004; 66(3): 301-7.

Saha SP, Bhattacharya S, Das SK, Maity B, Roy T, Raut DK. Epidemiological study of neurological disorders in a rural population of Eastern India. J Indian Med Assoc. 2003; 101(5): 299-304.

Samuel P, Antonisamy B, Raghupathy P, Richard J, Fall CHD. Socio-economic status and cardiovascular risk factors in rural and urban areas of Vellore, Tamilnadu, South India. Int J Epidemiol. 2012; 41(5): 1315-27.

Sangath, Columbia University, Alcohol Research Group, Public Health Institute, University of California San Francisco, London School of Hygiene and Tropical Medicine. Soryacher Asar Ani Hacher Amcho Shodh (SAAHAS) Goa Alcohol Use Study 2004-2008. Goa, India: Sangath.

Sauvaget C, Ramadas K, Thomas G, Thara S, Sankaranarayanan R. Prognosis criteria of casual systolic and diastolic blood pressure values in a prospective study in India. J Epidemiol Community Health. 2010; 64(4): 366–72.

Seby K, Chaudhury S, Chakraborty R. Prevalence of psychiatric and physical morbidity in an urban geriatric population. Indian J Psychiatry. 2011; 53(2): 121-7.

Shah A, Afzal M. Prevalence of diabetes and hypertension and association with various risk factors among different Muslim populations of Manipur, India. J Diabetes Metab Disord. 2013; 12(1): 52.

Shah C, Sheth NR, Solanki B, Shah N. To assess the prevalence of impaired glucose tolerance and impaired fasting glucose in Western Indian population. J Assoc Physicians India. 2013; 61(3): 179–84.

Shrivastava SR, Ghorpade AG. High prevalence of type 2 diabetes melitus and its risk factors among the rural population of Pondicherry, South India. J Res Health Sci. 2014; 14(4): 258-63.

Singh AK, Mani K, Krishnan A, Aggarwal P, Gupta SK. Prevalence, awareness, treatment and control of diabetes among elderly persons in an urban slum of Delhi. Indian J Community Med. 2012; 37(4): 236-9.

Singh RB, Bajaj S, Niaz MA, Rastogi SS, Moshiri M. Prevalence of type 2 diabetes mellitus and risk of hypertension and coronary artery disease in rural and urban population with low rates of obesity. Int J Cardiol. 1998; 66(1): 65-72.

Singh RB, Fedacko J, Pella D, Macejova Z, Ghosh S, de Amit K, Begom R, Tumbis ZA, Haque M, Vajpeyee SK, de Meester F, Sergey C, Agarwalo R, Muthusamy VV, Five City Study Group, Gupta AK. Prevalence and risk factors for prehypertension and hypertension in five Indian cities. Acta Cardiol. 2011; 66(1): 29-37.

Singh RB, Fedacko J, Pella D, Macejova Z, Ghosh S, de Amit K, Begom R, Tumbis ZA, Haque M, Vajpeyee SK, de Meester F, Sergey C, Agarwalo R, Muthusamy VV, Five City Study Group, Gupta AK. Prevalence and risk factors for prehypertension and hypertension in five Indian cities. Acta Cardiol. 2011; 66(1): 29-37.

Sree Chitra Tirunal Institute for Medical Sciences and Technology. India Sree Chitra Tirunal Institute for Medical Sciences and Technology Heart Failure Registry 2001-16. [Data shared for this analysis]

Sridharan SE, Unnikrishnan JP, Sukumaran S, Sylaja PN, Nayak SD, Sarma PS, Radhakrishnan K. Incidence, Types, Risk Factors, and Outcome of Stroke in a Developing Country. Stroke. 2009; 40(4): 1212-8.

Sriharibabu M, Himabindu Y, Kabir Z. Rheumatic heart disease in rural south India: A clinico-observational study. J Cardiovasc Dis Res. 2013; 4(1.0): 25-9.

St. John's Medical College and Research Institute, Population Health Research Institute, All India Institute of Health Sciences, Monilek Hospital and Research Centre, Government Medical College-Nagpur, KEM Hospital-Mumbai, Sri Ramachandra Medical College-Chennai, Amrita Institute of Medical Sciences, Christian Medical College-Ludhiana, Mediciti Hospital-Hyderabad, SSKM Hospital-Kolkata. India CREATE Registry Data 2001-2005. [Data shared for this analysis]

St. John's Medical College and Research Institute, Population Health Research Institute, Mahatma Gandhi Institute of Medical Sciences, Fortis Escort Hospital. INdian Stroke ProspectIve REgistry (INSPIRE) Data 2009-2014. [Data shared for this analysis]

State Bureau of Health Intelligence & Vital Statistics, Directorate of Health Services, Department of Health & Family Welfare, Government of Odisha. India - Odisha Medical Certification of Cause of Death Data 2009. Odisha, India: Department of Health & Family Welfare, Government of Odisha.

State Bureau of Health Intelligence & Vital Statistics, Directorate of Health Services, Department of Health & Family Welfare, Government of Odisha. India - Odisha Medical Certification of Cause of Death Data 2010. Odisha, India: Department of Health & Family Welfare, Government of Odisha

State Bureau of Health Intelligence & Vital Statistics, Directorate of Health Services, Department of Health & Family Welfare, Government of Odisha. India - Odisha Medical Certification of Cause of Death Data 2011. Odisha, India: Department of Health & Family Welfare, Government of Odisha.

State Bureau of Health Intelligence & Vital Statistics, Directorate of Health Services, Department of Health & Family Welfare, Government of Odisha. India - Odisha Medical Certification of Cause of Death Data 2012. Odisha, India: Department of Health & Family Welfare, Government of Odisha.

State Bureau of Health Intelligence & Vital Statistics, Directorate of Health Services, Department of Health & Family Welfare, Government of Odisha. India - Odisha Medical Certification of Cause of Death Data 2013. Odisha, India: Department of Health & Family Welfare, Government of Odisha.

Subramanian SV, Smith GD. Patterns, distribution, and determinants of under- and overnutrition: a population-based study of women in India. Am J Clin Nutr. 2006; 84(3): 633-40.

Thakur JS, Jeet G, Pal A, Singh S, Singh A, Deepti SS, et al. Profile of risk factors for non-communicable diseases in Punjab, Northern India: Results of a state-wide STEPS Survey. PloS one. 2016 11(7):e0157705.

Thakur JS, Negi PC, Ahluwalia SK, Vaidya NK. Epidemiological survey of rheumatic heart disease among school children in the Shimla Hills of northern India: prevalence and risk factors. J Epidemiol Community Health. 1996; 50(1): 62-7.
The INTERSALT Co-operative Research Group. Appendix tables. Centre-specific results by age and sex. J Hum Hypertens 1989; 3(5):331-407.
Tiwari S, Hopke PK, Pipal AS, Srivastava AK, Bishta DS, Tiwari S, Singh AK, Sonie VK, Attrie SD. Intra-urban variability of particulate matter (PM2.5 and PM10) and its relationship with optical properties of aerosols over Delhi, India. Atmos Res. 2015; 166: 223-32.
Tobacco Sales Data. Personal Correspondence with Christopher Tan, 1997-2012. [Data shared for this analysis]
Tyrovolas S, Koyanagi A, Garin N, Olaya B, Ayuso-Mateos JL, Miret M, Chatterji S, Tobiasz-Adamczyk B, Koskinen S, Leonardi M, Haro JM. Determinants of the components of arterial pressure among older adults--the role of anthropometric and clinical factors: a multi-continent study. Atherosclerosis. 2015; 238(2): 240–9.
U.S. Department of Agriculture (USDA). USDA Global Tobacco Database 1960-2005. Washington DC, United States: USDA.
Unnikrishnan JP, Sylaja S, Nayak SD, Radhakrishnan K. India - Trivandrum Stroke Registry 2005. [Data shared for this analysis]
Vaz NC, Ferreira AM, Kulkarni MS, Vaz FS. Prevalence of diabetes mellitus in a rural population of Goa, India. Natl Med J India. 2011; 24(1): 16-8.
Vijayakumar G, Arun R, Kutty VR. High prevalence of type 2 diabetes mellitus and other metabolic disorders in rural Central Kerala. J Assoc Physicians India. 2009; 57: 563-7.
Vijayaraghavan K, Rao DH. Diet & nutrition situation in rural India. Indian J Med Res. 1998; 108:243-53.
Walia R, Bhansali A, Ravikiran M, Ravikumar P, Bhadada SK, Shanmugasundar G, Dutta P, Sachdeva N. High prevalence of cardiovascular risk factors in Asian Indians: a community survey - Chandigarh Urban Diabetes Study (CUDS). Indian J Med Res. 2014; 139(2): 252–9.
World Bank. Survey of Living Conditions 1997-1998, Uttar Pradesh and Bihar. Washington D.C., United States: World Bank.
World Health Organization (WHO). WHO Urban Ambient Air Pollution Database Draft 2016. Geneva, Switzerland: WHO.
World Health Organization. Study on Global Ageing and Adult Health (SAGE) Pilot Study 2005 Data from the Data Archive of Social Research on Aging. Los Altos, United States: Sociometrics Corporation. Available from: http://home.socio.com/age2728.php
Xavier D, Pais P, Devereaux PJ, Xie C, Prabhakaran D, Reddy KS, et al. Treatment and outcomes of acute coronary syndromes in India (CREATE): a prospective analysis of registry data. The Lancet. 2008;371(9622):1435–1442.
Zaman FA, Borang A. Prevalence of diabetes mellitus amongst rural hilly population of North Eastern India and its relationship with associated risk factors and related co-morbidities. J Nat Sci Biol Med. 2014; 5(2): 383–8.
Zaman FA, Pal R, Zaman GS, Swati IA, Kayyum A. Glucose indices, frank and undetected diabetes in relation to hypertension and anthropometry in a South Indian rural population. Indian J Public Health. 2011; 55(1): 34–7.

3. Change in death and DALY rates due to cardiovascular diseases by sex in the states of India grouped by epidemiological transition level, 1990 to 2016

Sex	State group (population in 2016)	Crude deaths per 100,000 (95% uncertainty interval)			Age-standardised, percent change 1990 to 2016 (95% uncertainty interval)	Crude DALYs per 100,000 (95% uncertainty interval)			Age-standardised percent change 1990 to 2016 (95% uncertainty interval)
		1990	2016	Percent change 1990 to 2016		1990	2016	Percent change 1990 to 2016	
Both sexes	Low ETL (626 million)	127 (113 to 138)	169 (153 to 181)	33.6 (24.0 to 44.9)	6.6 (-1.7 to 15.9)	3,555 (3,220 to 3,827)	4,166 (3,851 to 4,447)	17.2 (9.1 to 26.6)	-1.6 (-8.5 to 6.2)
	Lower-middle ETL (92 million)	144 (128 to 157)	200 (185 to 216)	39.5 (25.1 to 56.5)	-0.5 (-10.4 to 12.3)	3,942 (3,556 to 4,286)	4,833 (4,435 to 5,270)	22.6 (9.5 to 37.2)	-7.8 (-17.2 to 2.9)
	Higher-middle ETL (446 million)	178 (161 to 192)	244 (229 to 259)	36.8 (26.7 to 49.2)	-7.3 (-14.8 to 1.9)	4,882 (4,475 to 5,230)	5,765 (5,423 to 6,104)	18.1 (9.5 to 28.4)	-14.8 (-20.9 to -7.1)
	High ETL (152 million)	198 (179 to 216)	276 (255 to 295)	39.3 (26.7 to 55.1)	-13.1 (-20.5 to -3.5)	5,258 (4,814 to 5,698)	6,183 (5,717 to 6,629)	17.6 (6.6 to 30.6)	-19.9 (-27.2 to -11.3)
	India (1,316 million)	156 (140 to 166)	209 (197 to 219)	34.3 (26.6 to 43.7)	-2.5 (-8.8 to 5.5)	4,278 (3,929 to 4,515)	4,987 (4,726 to 5,207)	16.6 (10.7 to 23.8)	-10.2 (-15.0 to -4.1)
Men	Low ETL	134 (118 to 148)	186 (167 to 202)	38.9 (26.7 to 52.8)	13.2 (3.2 to 24.9)	3,799 (3,430 to 4144)	4,728 (4,330 to 5,092)	24.5 (13.4 to 36.7)	5.1 (-3.9 to 15.3)
	Lower-middle ETL	154 (136 to 172)	221 (198 to 245)	43.6 (23.7 to 67.2)	6.1 (-7.6 to 22.8)	4,411 (3,924 to 4,921)	5,740 (5,101 to 6,400)	30.1 (11.9 to 52.8)	-1.3 (-14.7 to 14.7)
	Higher-middle ETL	191 (170 to 208)	272 (252 to 290)	42.1 28.9 to 57.2)	-0.1 (-9.3 to 11.8)	5,381 (4,903 to 5,834)	6,736 (6,268 to 7,196)	25.2 (13.6 to 38.6)	-7.9 (-16.2 to 1.8)
	High ETL	229 (204 to 254)	315 (288 to 344)	37.7 (21.1 to 56.0)	-8.9 (-19.0 to 3.1)	6,212 (5,579 to 6,887)	7,487 (6,810 to 8,253)	20.5 (4.9 to 37.0)	-15.9 (-26.0 to -4.7)
	India	168 (151 to 180)	232 (217 to 243)	38.1 (28.2 to 49.1)	3.9 (-3.7 to 13.2)	4,715 (4,335 to 5,020)	5,787 (5,445 to 6,068)	22. 7 (14.1 to 32.1)	-4.1 (-10.7 to 3.3)
Women	Low ETL	119 (96 to 139)	151 (127 to 167)	27.0 (12.3 to 43.8)	-0.7 (-13.4 to 13.1)	3,288 (2,777 to 3,705)	3,552 (3,114 to 3,908)	8.0 (-3.0 to 20.9)	-9.6 (-19.4 to 1.9)
	Lower-middle ETL	133 (110 to 154)	178 (155 to 202)	34.2 (12.8 to 61.9)	-7.3 (-22.1 to 11.7)	3,441 (2,928 to 3,921)	3,847 (3,386 to 4,359)	11.8 (-4.8 to 33.1)	-16.2 (-29.1 to 0.2)
	Higher-middle ETL	165 (138 to 187)	214 (192 to 234)	30.2 (14.4 to 49.5)	-14.5 (-25.4 to -1.4)	4,349 (3,713 to 4,854)	4,728 (4,304 to 5,119)	8.7 (-3.0 to 23.6)	-22.8 (-31.6 to -11.7)
	High ETL	167 (139 to 190)	237 (208 to 262)	41.8 (22.9 to 66.6)	-16.7 (-27.4 to -2.2)	4,278 (3,645 to 4,828)	4,859 (4,333 to 5,366)	13.6 (-1.1 to 32.2)	-24.4 (-34.0 to -11.7)
	India	142 (118 to 161)	184 (164 to 198)	29.5 (17.0 to 45.0)	-9.0 (-18.7 to 2.7)	3,809 (3,266 to 4,202)	4,128 (3,754 to 4,397)	8.4 (-0.8 to 20.2)	-17.5 (-24.8 to -8.1)

DALY is disability-adjusted life-year. ETL is epidemiological transition level.

4. Change in prevalence of IHD, stroke, and RHD in the states of India grouped by epidemiological transition level, 1990 to 2016

	State group	Crude prevalence per 100,000 (95% uncertainty interval)			Age-standardised percent change 1990 to 2016 (95% uncertainty interval)
		1990	2016	Percent change 1990 to 2016	
**Ischaemic heart disease**	Low ETL	1,028<br>(983 to 1,073)	1,508<br>(1,429 to 1,582)	46.7<br>(65.2 to 72.2)	14.1<br>(11.9 to 16.2)
	Lower-middle ETL	1,068<br>(1,022 to 1,113)	1,693<br>(1,607 to 1,773)	58.5<br>(55.3 to 61.8)	11.4<br>(8.9 to 13.7)
	Higher-middle ETL	1,290<br>(1,240 to 1,337)	2,029<br>(1,931 to 2,122)	57.3<br>(54.0 to 60.0)	5.3<br>(3.2 to 7.3)
	High ETL	1,459<br>(1,397 to 1,522)	2,462<br>(2,338 to 2,580)	68.7<br>(65.2 to 72.2)	6.2<br>(3.9 to 8.4)
	India	1,181<br>(1,133 to 1,229)	1,807<br>(1,717 to 1,893)	53.0<br>(50.2 to 55.4)	9.4<br>(7.3 to 11.2)
**Stroke**	Low ETL	291<br>(274 to 312)	420<br>(387 to 459)	44.3<br>(37.4 to 50.9)	14.3<br>(8.4 to 19.7)
	Lower-middle ETL	296<br>(279 to 317)	477<br>(438 to 518)	61.3<br>(53.4 to 68.4)	15.2<br>(9.6 to 20.2)
	Higher-middle ETL	355<br>(333 to 381)	574<br>(530 to 624)	61.7<br>(54.6 to 68.7)	10.7<br>(5.7 to 15.5)
	High ETL	372<br>(351 to 398)	625<br>(578 to 678)	67.9<br>(60.2 to 75.1)	8.2<br>(3.1 to 12.9)
	India	325<br>(306 to 348)	500<br>(461 to 543)	53.8<br>(47.2 to 60.3)	12.2<br>(7.1 to 17.0)
**Rheumatic heart disease**	Low ETL	603<br>(538 to 674)	577<br>(510 to 648)	-4.4<br>(-1.1 to -7.8)	-11.1<br>(-14.1 to -8.0)
	Lower-middle ETL	596<br>(537 to 660)	603<br>(539 to 672)	1.1<br>(-3.1 to 5.7)	-10.9<br>(-14.3 to -6.8)
	Higher-middle ETL	641<br>(581 to 705)	662<br>(598 to 732)	3.2<br>(0.5 to 5.8)	-9.4<br>(-11.9 to -7.0)
	High ETL	620<br>(551 to 694)	614<br>(542 to 689)	-1.1<br>(-5.2 to 3.1)	-14.4<br>(-17.9 to -10.9)
	India	618<br>(557 to 686)	612<br>(680 to 546)	-1.1<br>(-3.4 to 1.3)	-10.8<br>(-13.0 to -8.7)

IHD is ischaemic heart disease. RHD is rheumatic heart disease. ETLis epidemiological transition level.

5. Change in prevalence of IHD and stroke in the states of India, 1990 to 2016

States of India*	IHD				Stroke			
	Crude prevalence per 100,000 (95% uncertainty interval)			Age-standardised percent change 1990 to 2016 (95% uncertainty interval)	Crude prevalence per 100,000 (95% uncertainty interval)			Age-standardised percent change 1990 to 2016 (95% uncertainty interval)
	1990	2016	Percent change 1990 to 2016		1990	2016	Percent change 1990 to 2016	
Bihar	1,023 (978 to 1,070)	1,501 (1,421 to 1,578)	46.7 (42.8 to 50.5)	16.2 (13.1 to 19.2)	275 (258 to 296)	411 (379 to 451)	49.4 (41.4 to 58.8)	20.8 (14.5 to 28.5)
Jharkhand	969 (924 to 1,014)	1,477 (1,398 to 1,560)	52.5 (48.0 to 56.9)	15.2 (11.7 to 18.3)	296 (277 to 318)	424 (391 to 463)	43.1 (34.9 to 51.0)	10.6 (4.3 to 17.0)
Uttar Pradesh	1,062 (1,015 to 1,109)	1,434 (1,354 to 1,509)	65.6 (61.2 to 70.2)	9.1 (6.1 to 12.0)	244 (226 to 264)	325 (293 to 359)	69.6 (60.7 to 79.2)	13.9 (7.9 to 20.2)
Rajasthan	1,053 (1,007 to 1,098)	1,475 (1,403 to 1,548)	40.1 (36.6 to 43.6)	6.7 (4.0 to 9.4)	245 (227 to 267)	355 (316 to 396)	44.8 (33.3 to 55.6)	12.4 (3.1 to 21.1)
Meghalaya	828 (787 to 869)	1,180 (1,117 to 1,245)	42.5 (38.5 to 46.7)	15.0 (12.0 to 18.5)	249 (234 to 268)	365 (338 to 396)	46.7 (39.4 to 53.9)	20.2 (13.9 to 26.5)
Assam	788 (746 to 830)	1,295 (1,220 to 1,374)	64.4 (59.7 to 69.6)	14.2 (11.0 to 17.8)	353 (329 to 383)	548 (502 to 603)	55.2 (46.5 to 63.7)	9.2 (3.1 to 15.2)
Chhattisgarh	906 (861 to 952)	1,581 (1,494 to 1,664)	74.4 (70.1 to 79.0)	22.3 (19.1 to 25.5)	361 (339 to 388)	585 (542 to 634)	61.7 (53.2 to 70.8)	15.4 (9.4 to 21.9)
Madhya Pradesh	1,132 (1,081 to 1,185)	1,718 (1,633 to 1,805)	51.8 (48.2 to 55.4)	15.5 (12.6 to 18.2)	353 (332 to 378)	512 (474 to 555)	44.8 (37.3 to 52.9)	11.8 (6.0 to 18.2)
Odisha	999 (952 to 1,051)	1,726 (1,630 to 1,823)	72.8 (68.3 to 77.6)	20.5 (17.4 to 23.9)	399 (373 to 429)	647 (598 to 703)	62.2 (53.2 to 71.8)	16.1 (9.5 to 23.1)
Arunachal Pradesh	770 (733 to 812)	1,017 (961 to 1,076)	32.0 (28.4 to 36.1)	10.4 (7.3 to 14.0)	248 (232 to 267)	343 (315 to 375)	38.4 (31.0 to 46.0)	16.4 (10.1 to 22.8)
Mizoram	863 (823 to 906)	1,343 (1,271 to 1,417)	55.6 (51.9 to 59.9)	6.0 (3.5 to 9.1)	227 (213 to 244)	377 (347 to 409)	66.1 (57.7 to 74.1)	14.8 (9.2 to 20.4)
Nagaland	955 (906 to 1,002)	1,191 (1,126 to 1,256)	24.7 (21.5 to 28.4)	10.4 (7.2 to 13.9)	338 (319 to 361)	431 (402 to 467)	27.3 (21.2 to 33.6)	12.3 (6.3 to 18.2)
Uttarakhand	1,095 (1,041 to 1,147)	1,813 (1,711 to 1,908)	66.0 (62.1 to 69.9)	6.6 (3.9 to 9.2)	299 (281 to 320)	507 (468 to 551)	68.8 (61.1 to 77.3)	10.8 (5.5 to 16.2)
Gujarat	1,084 (1,038 to 1,131)	1,740 (1,652 to 1,821)	60.5 (56.5 to 64.5)	12.0 (9.1 to 14.9)	287 (269 to 307)	467 (428 to 508)	63.1 (54.4 to 71.0)	15.9 (9.5 to 21.8)
Tripura	1,036 (985 to 1,086)	1,539 (1,458 to 1,623)	48.6 (45.0 to 52.8)	11.7 (9.0 to 14.8)	418 (393 to 449)	623 (578 to 677)	48.8 (41.7 to 56.2)	13.2 (7.9 to 18.8)
Sikkim	986 (939 to 1,035)	1,472 (1,391 to 1,551)	49.3 (45.1 to 53.7)	14.5 (11.3 to 18.0)	263 (247 to 283)	395 (363 to 428)	49.9 (41.6 to 58.5)	16.8 (9.8 to 23.9)
Manipur	940 (892 to 985)	1,414 (1,338 to 1,495)	50.5 (46.5 to 54.8)	9.1 (6.4 to 12.2)	334 (314 to 357)	506 (470 to 552)	51.8 (44.2 to 59.3)	11.3 (5.8 to 17.2)
Haryana	1,274 (1,215 to 1,332)	1,864 (1,771 to 1,957)	46.4 (42.9 to 49.8)	13.3 (10.6 to 16.0)	289 (269 to 312)	454 (411 to 500)	57.3 (46.9 to 67.2)	19.9 (11.5 to 27.5)
Delhi	1,105 (1,057 to 1,151)	1,591 (1,517 to 1,665)	44.0 (40.2 to 48.0)	1.0 (-1.6 to 3.9)	279 (260 to 300)	427 (393 to 464)	53.2 (44.6 to 62.2)	8.2 (1.8 to 14.9)
Telangana	1,129 (1,080 to 1,181)	1,779 (1,685 to 1,873)	57.6 (53.2 to 61.7)	2.6 (-0.3 to 5.3)	332 (311 to 357)	536 (494 to 584)	61.4 (52.8 to 70.3)	8.7 (2.7 to 14.6)
Andhra Pradesh	1,146 (1,088 to 1,203)	1,924 (1,814 to 2,030)	67.9 (63.6 to 72.6)	10.9 (8.0 to 13.9)	351 (329 to 378)	580 (536 to 630)	65.4 (56.8 to 74.8)	13.0 (7.0 to 19.3)
Jammu and Kashmir	1,013 (966 to 1,062)	1,570 (1,487 to 1,654)	54.9 (50.8 to 58.9)	9.3 (6.4 to 12.1)	283 (265 to 303)	415 (377 to 454)	46.8 (37.4 to 55.4)	6.8 (-0.3 to 13.1)
Karnataka	1,215 (1,167 to 1,264)	1,931 (1,828 to 2,026)	58.9 (54.3 to 63.1)	6.8 (3.7 to 9.7)	320 (297 to 346)	521 (475 to 572)	62.7 (52.8 to 72.9)	10.9 (4.0 to 17.7)
West Bengal	1,250 (1,201 to 1,299)	2,075 (1,978 to 2,175)	42.7 (39.4 to 46.2)	9.6 (7.1 to 12.3)	438 (410 to 471)	739 (685 to 806)	37.0 (29.5 to 44.5)	4.5 (-1.6 to 10.4)
Maharashtra	1,555 (1,498 to 1,607)	2,343 (2,233 to 2,448)	50.7 (46.7 to 54.5)	-0.3 (-2.9 to 2.3)	344 (321 to 371)	547 (501 to 598)	59.0 (49.9 to 67.7)	7.9 (1.7 to 13.7)
Union territories other than Delhi	1,014 (965 to 1,063)	1,447 (1,371 to 1,526)	35.0 (31.2 to 38.9)	12.4 (9.1 to 15.6)	322 (303 to 345)	442 (406 to 481)	33.2 (23.6 to 43.4)	12.0 (3.2 to 20.3)
Himachal Pradesh	1,290 (1,229 to 1,355)	2,112 (1,996 to 2,226)	63.7 (59.3 to 68.4)	11.8 (8.7 to 14.9)	354 (331 to 379)	567 (519 to 619)	60.3 (50.2 to 70.4)	10.8 (3.8 to 17.9)
Punjab	1,538 (1,473 to 1,607)	2,411 (2,290 to 2,527)	56.8 (52.6 to 60.7)	3.9 (1.0 to 6.6)	383 (360 to 410)	623 (577 to 673)	62.8 (53.6 to 71.0)	9.9 (3.9 to 15.5)
Tamil Nadu	1,411 (1,347 to 1,474)	2,364 (2,240 to 2,483)	67.5 (63.2 to 71.9)	6.0 (3.0 to 9.0)	353 (332 to 379)	579 (533 to 630)	63.9 (55.2 to 72.3)	7.8 (1.7 to 13.3)
Goa	965 (917 to 1,013)	1,942 (1,842 to 2,047)	101.4 (96.6 to 106.8)	14.0 (11.6 to 17.0)	372 (350 to 398)	688 (634 to 747)	85.0 (75.5 to 95.3)	6.5 (1.0 to 12.1)
Kerala	1,548 (1,479 to 1,617)	2,793 (2,651 to 2,933)	80.4 (75.9 to 85.0)	7.3 (4.6 to 10.0)	405 (379 to 435)	730 (672 to 795)	80.3 (71.0 to 89.9)	7.9 (2.2 to 13.5)

*****The states are listed in increasing order of epidemiological transition level in 2016. IHD is ischaemic heart disease.

6. Percent contribution of major risk factors to cardiovascular diseases DALYs in India by sex, 2016

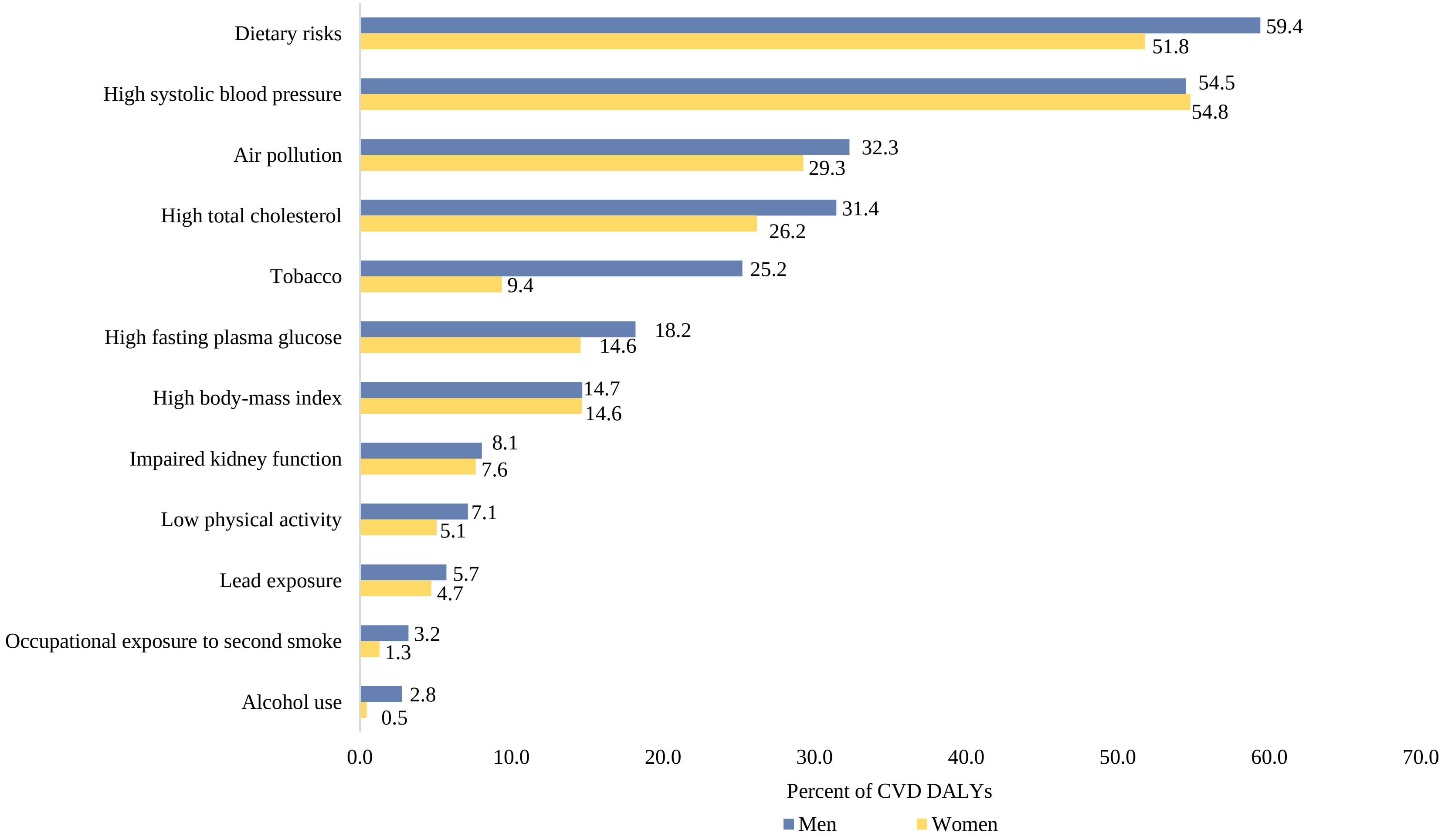

The cumulative impact of risk factors is not the simple addition of their individual contributions as the risk factors overlap, and also because the population attributable fractions from components can add up to more than their sum even if they are independent. DALY is disability-adjusted life-year. CVD is cardiovascular diseases.
